# Supplementary material for: The E3 ubiquitin ligase adaptor KLHL8 targets ZAR1 to regulate maternal mRNA degradation in oocytes
Source: EMBO Rep. 2025 Jul 28;26(17):4364–87. doi: 10.1038/s44319-025-00537-y (PMC12420792; doi:10.1038/s44319-025-00537-y)
Supplement: Supplementary file 1 — Appendix [file 44319_2025_537_MOESM1_ESM.pdf]

Appendix for: "The E3 ubiquitin ligase adaptor KLHL8 targets ZAR1 to regulate maternal mRNA degradation in oocytes"

Appendix Figure S1……………………………………………………………………………2

Appendix Figure S2……………………………………………………………………………3

Appendix Figure S3……………………………………………………………………………4

Appendix Figure S4……………………………………………………………………………5

Appendix Figure S5……………………………………………………………………………6

Appendix Figure S6……………………………………………………………………………7

Appendix Figure S7……………………………………………………………………………8

Appendix Table S1 ……………………………………………………………………………9

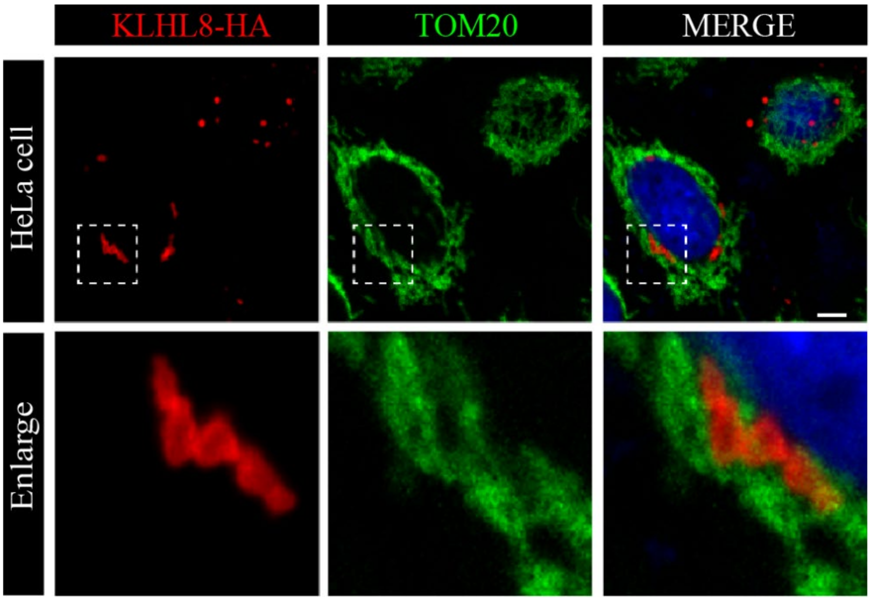

Appendix Figure S1. The localization of KLHL8 in HeLa cells.

Representative immunofluorescence images of KLHL8 and TOM20 in HeLa cells. Red, KLHL8-HA; Green, TOM20. Scale bar, 5 μm.

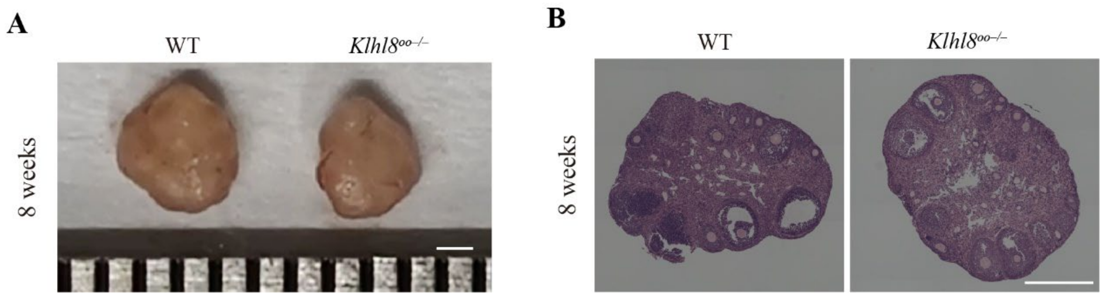

Appendix Figure S2. The ovary size and histology of WT and *Klhl8*$^{oo–/–}$ mice.

(**A**) Morphology of ovaries from WT and *Klhl8*$^{oo–/–}$ mice at 8 weeks. Scale bar = 0.5 mm.

(**B**) Hematoxylin staining of ovary sections from WT and *Klhl8*$^{oo–/–}$ mice at 8 weeks. Scale bar, 1 mm.

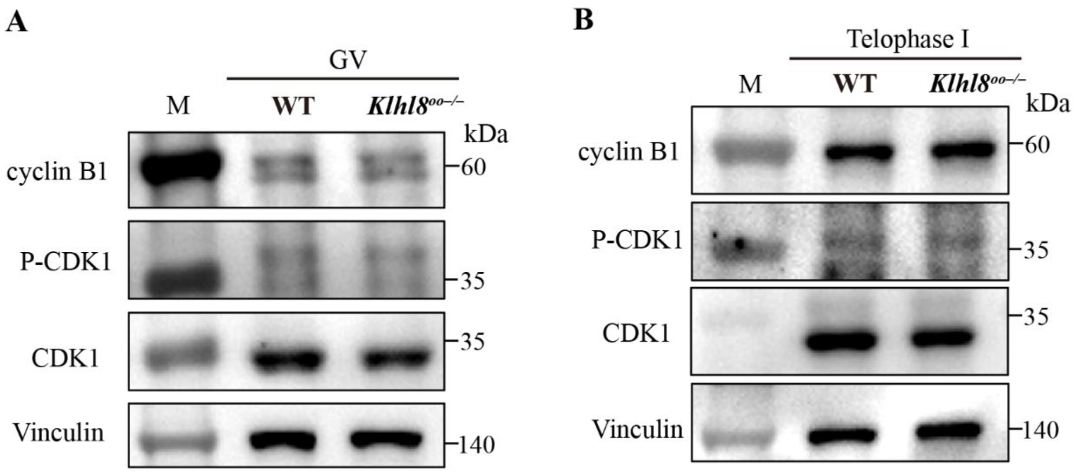

Appendix Figure S3. Activity of MPF in WT and *Klhl8*$^{oo-/-}$ oocytes.

(**A**) Activity of MPF in WT and *Klhl8*$^{oo-/-}$ oocytes at GV stage. (**B**) Activity of MPF in WT and *Klhl8*$^{oo-/-}$ oocytes at Telophase I (the final stage of meiosis I) stage.

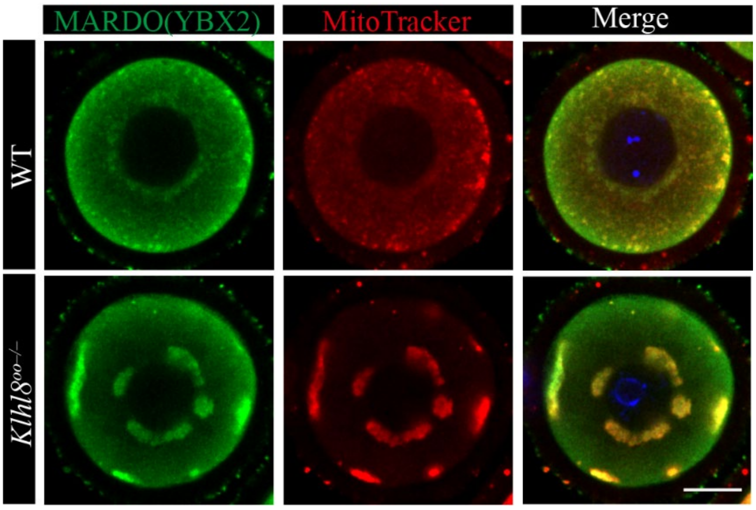

Appendix Figure S4. ***Klhl8*** **deletion causes MARDO coalescence in oocytes.**

Representative immunofluorescence images of GV oocytes collected from WT and *Klhl8*$^{oo-/-}$ mice. Green, MARDO (YBX2); Red, mitochondria (MitoTracker). Scale bar, 20 μm.

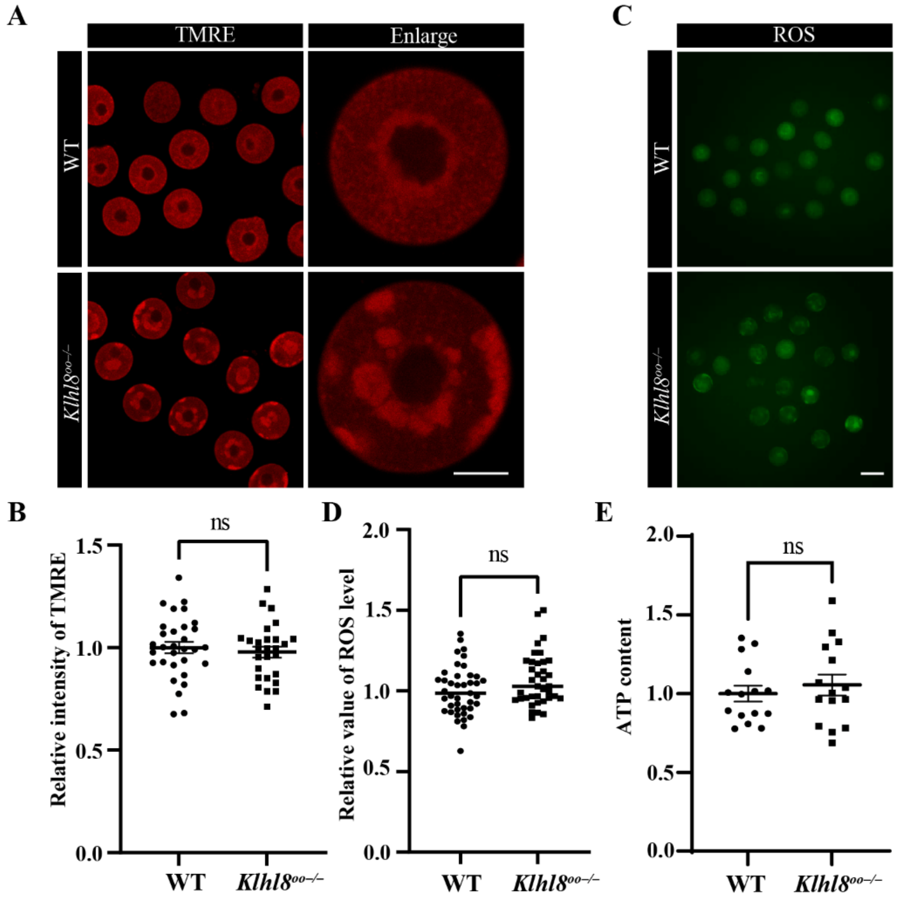

Appendix Figure S5. Detection of mitochondrial function in WT and *Klhl8*$^{oo-/-}$ oocytes.

(**A**) Representative immunofluorescence images of MMP level in WT and *Klhl8*$^{oo-/-}$ oocytes. Scale bar, 20 µm. (**B**) Quantification of MMP fluorescence intensity in WT and *Klhl8*$^{oo-/-}$ oocytes. Each point represents one sample (WT, n = 35; *Klhl8*$^{oo-/-}$, n = 33). (**C**) Representative immunofluorescence images of ROS level in WT and *Klhl8*$^{oo-/-}$ oocytes. Scale bar, 100 µm. (**D**) Quantification of ROS fluorescence intensity in WT and *Klhl8*$^{oo-/-}$ oocytes (WT, n = 60; *Klhl8*$^{oo-/-}$, n = 60). (E) The ATP content in WT and *Klhl8*$^{oo-/-}$ oocytes. Each point represents a sample (WT, n = 20; *Klhl8*$^{oo-/-}$, n = 20). Data information: The fluorescence intensity was quantified by Image J software. In (**B**, **D-E**), data are presented as mean ± SEM. Statistical significance was determined using an unpaired two-tailed t test: ns = not significant.

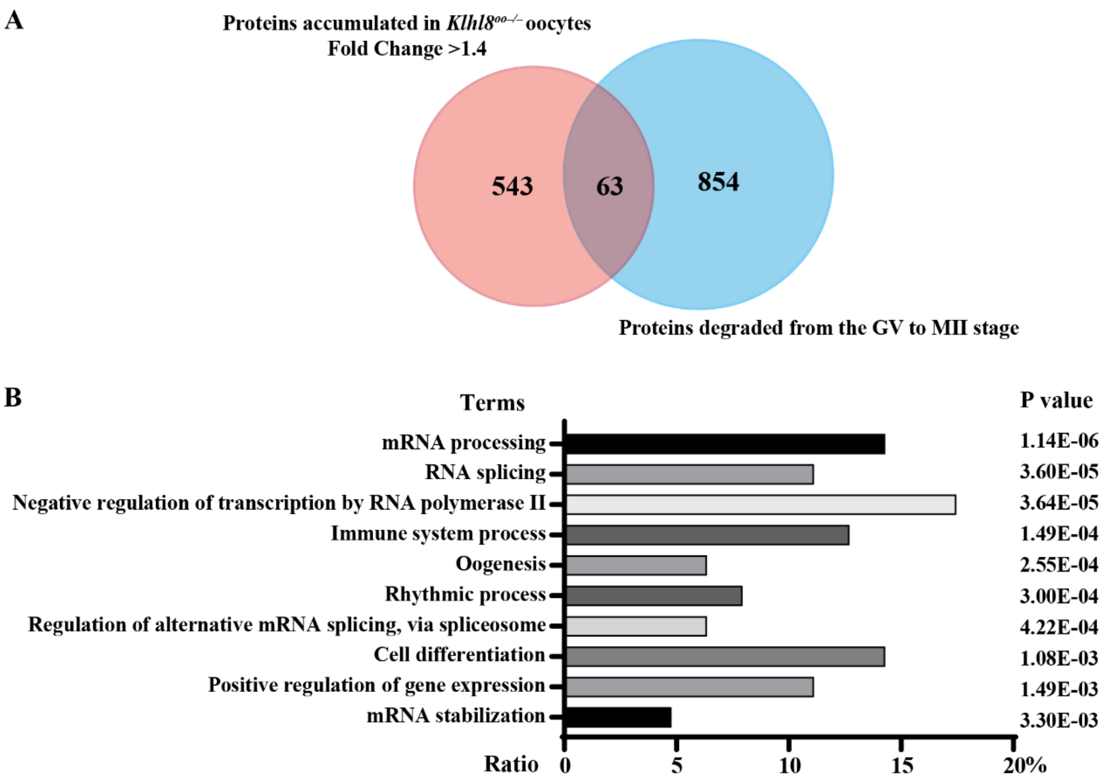

Appendix Figure S6. Overlap analysis of proteins who accumulated in *Klhl8*$^{oo-/-}$ oocytes and degraded in the transition from the GV to MII stage.

(**A**) Venn diagrams showing the overlap proteins who accumulated in *Klhl8*$^{oo-/-}$ GV oocytes and degraded in the transition from the GV to MII stage. The latter data were from a previous study (Sun *et al*, 2023). (**B**) Gene ontology (GO) enrichment analysis of the genes coding shared proteins.

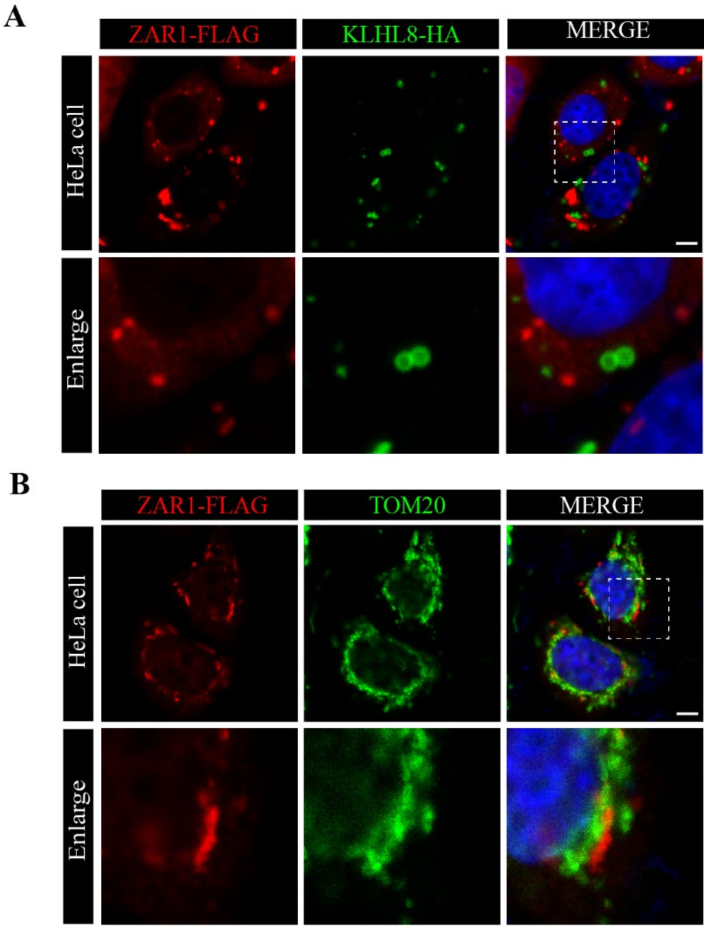

Appendix Figure S7. The localization of KLHL8 in ZAR1-overexpressed HeLa cells.

(**A**) Representative immunofluorescence images of ZAR1 and KLHL8 in HeLa cells. Red, ZAR1-FLAG; Green, KLHL8-HA. Scale bar, 5 µm. (**B**) Representative immunofluorescence images of ZAR1 and TOM20 in HeLa cells. Red, ZAR1-FLAG; Green, TOM20. Scale bar, 5 µm.

Appendix Table S1. List of primers used in PCR and qRT-PCR.

Primer name	Sequence	Purpose
Klhl8-F	CGAATCTGACCAATGGAGCACAG	qRT-PCR
Klhl8-R	GGTGATACCTCTCCACACTGGA	qRT-PCR
Zar1-F	CGGCTACTATCACTGCAAGGAC	qRT-PCR
Zar1-R	GACAGGTGATGTCCTCCACTCT	qRT-PCR
Dazl-F	TGACGTGGATGTGCAGAAGATAG	qRT-PCR
Dazl-R	CAAAGGACGTGGCTGCACATGA	qRT-PCR
Fgf8-F	TTGGAAGCAGAGTCCGAGTTCG	qRT-PCR
Fgf8-R	GCCGTGTAGTTGTTCTCCAGCA	qRT-PCR
Ftdc2-F	ATCCTGCTAGCCCTAAAGGCTG	qRT-PCR
Ftdc2-R	GAGAAAGTTGACTTTCCAGATACTC	qRT-PCR
Khdc3-F	ACTGTACGTGGAACCTCGGCTA	qRT-PCR
Khdc3-R	CCAAATCTCAGCTCGCCTTTCAG	qRT-PCR
Zp2-F	CTCATCACACGCTATCTGCGCA	qRT-PCR
Zp2-R	TCCTCAGGGATGCTCCATTGTC	qRT-PCR
Padi6-F	CTGAGCGAGAAGAGCAAAGTGC	qRT-PCR
Padi6-R	ATGACACCGTCTTGTGAGGAGC	qRT-PCR
Lhx8-F	AGCACAGTTCGCTCAGGACAAC	qRT-PCR
Lhx8-R	GCTGAGGAAGAATGGTTGGGAC	qRT-PCR
Tcp1-F	TTTGTGGAAGCTGGTGCCATGG	qRT-PCR
Tcp1-R	CTCTTCCGCTTGTCCCAACATC	qRT-PCR
Paip2-F	GAGGATAATCCATTTGCAGAGTAC	qRT-PCR
Paip2-R	AGCATTTCTTGGAAACAGCGTTCA	qRT-PCR
Klhl8-F2	ATTGCATGACTACTGTGTAGTTCC	Mouse genotype identification
Klhl8-R2	TTCATCTCCTAAGTTAGCCACACG	Mouse genotype identification
ZP3-F	CAGATGAGGTTTGAGGCCACAG	Mouse genotype identification
ZP3-R	GCGAACATCTTCAGGTTCTGC	Mouse genotype identification
Oligo (dT)	Cy3-TTTTTTTTTTTTTTTTTTTTTT	mRNA-FISH
