# Supplementary figures and images for: The E3 ubiquitin ligase adaptor KLHL8 targets ZAR1 to regulate maternal mRNA degradation in oocytes (part 1 of 2)
Source: EMBO Rep. 2025 Jul 28;26(17):4364–87. doi: 10.1038/s44319-025-00537-y (PMC12420792; doi:10.1038/s44319-025-00537-y)

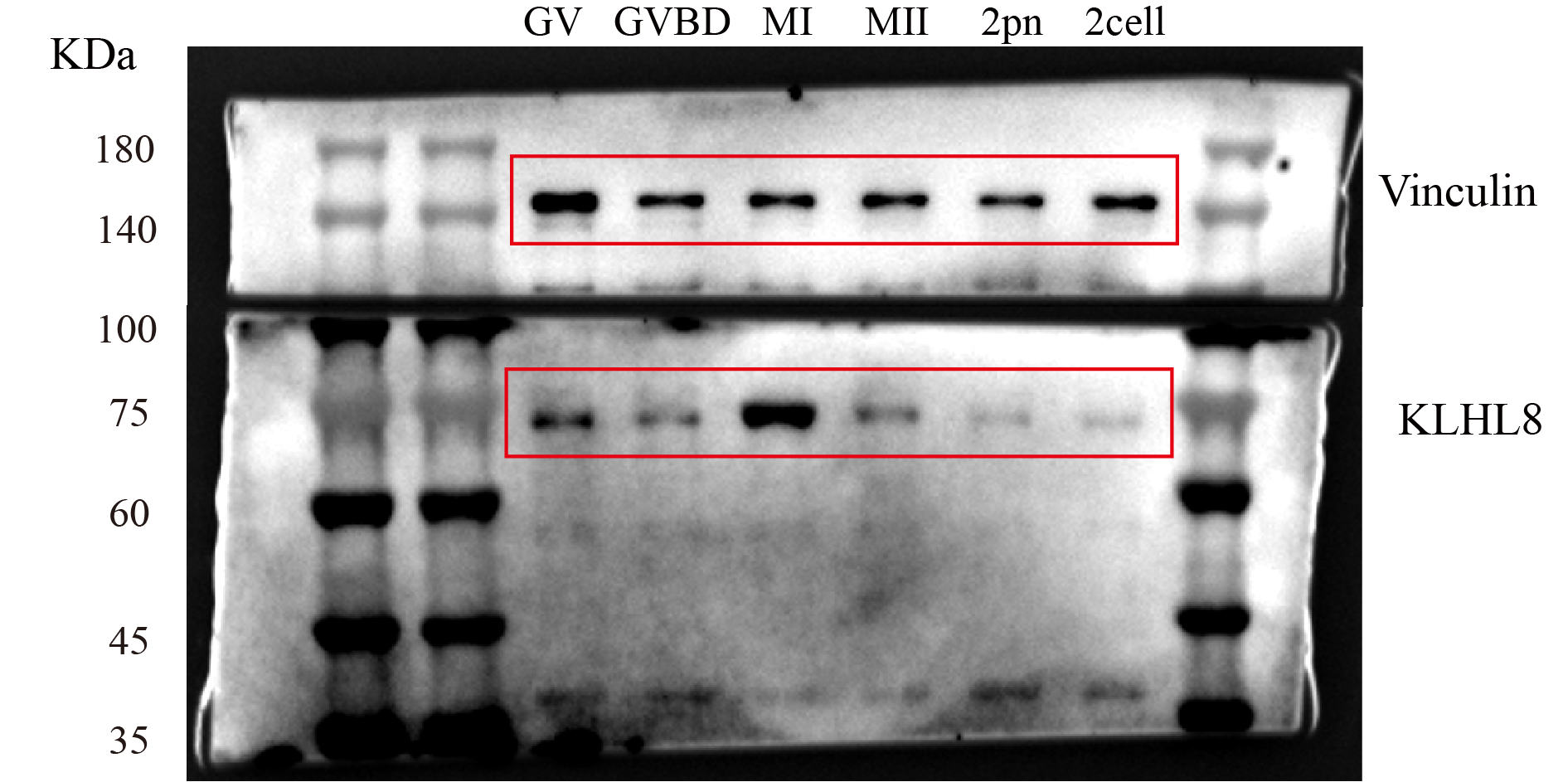

Supplement: Supplementary file 5 — Source data Fig. 1 [file 44319_2025_537_MOESM5_ESM.zip › 1B/1B replicate/1B.tif]

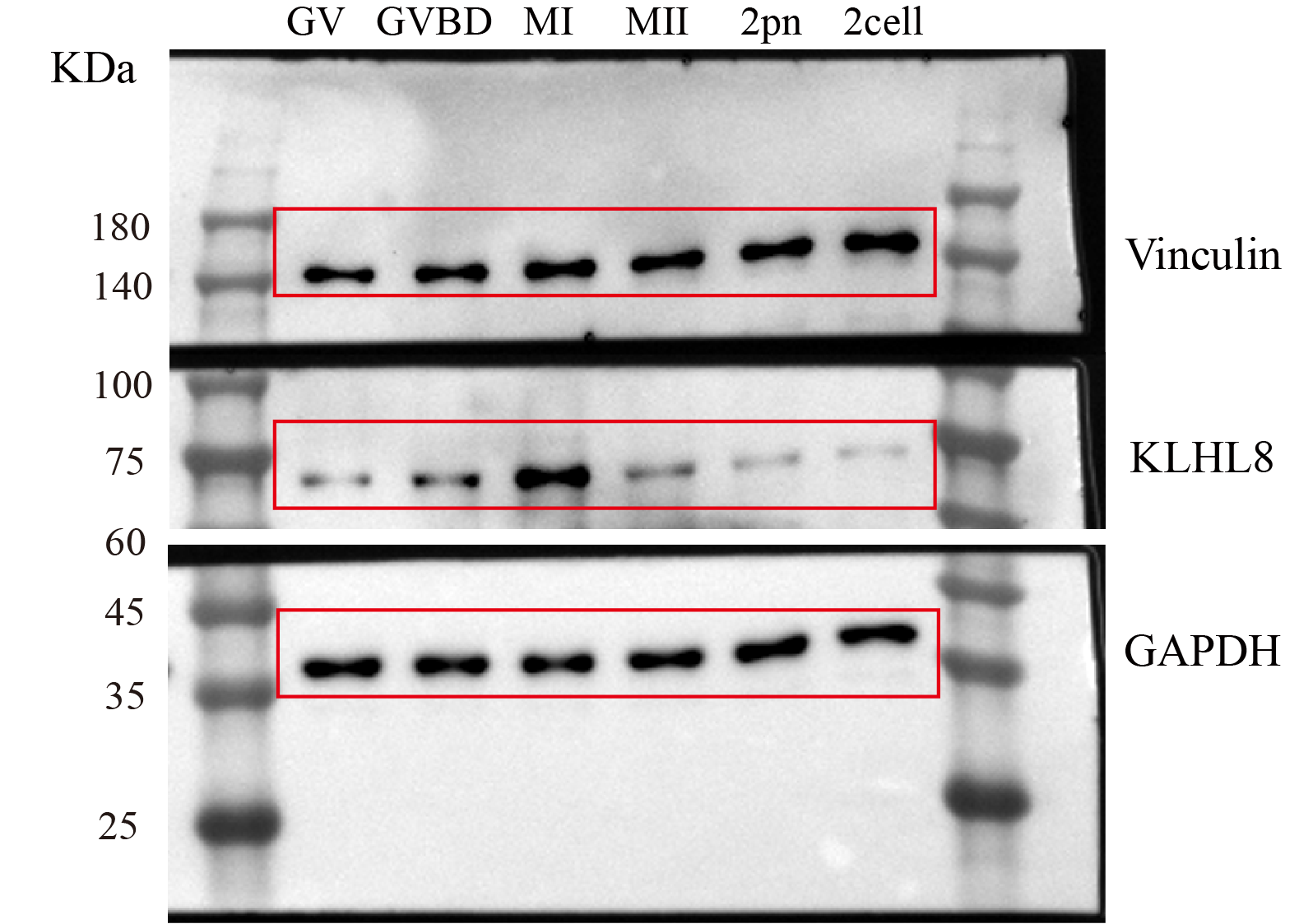

Supplement: Supplementary file 5 — Source data Fig. 1 [file 44319_2025_537_MOESM5_ESM.zip › 1B/1B.tif]

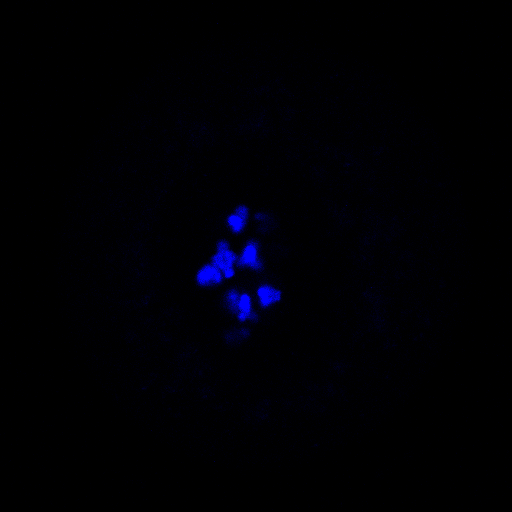

Supplement: Supplementary file 5 — Source data Fig. 1 [file 44319_2025_537_MOESM5_ESM.zip › 1C/GVBD/GVBD_DNA.tif]

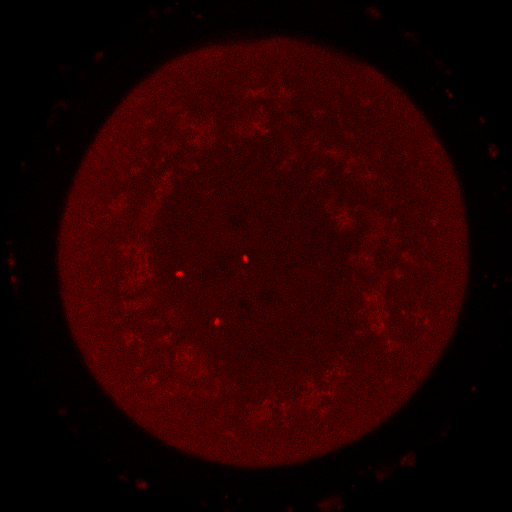

Supplement: Supplementary file 5 — Source data Fig. 1 [file 44319_2025_537_MOESM5_ESM.zip › 1C/GVBD/GVBD_KLHL8.tif]

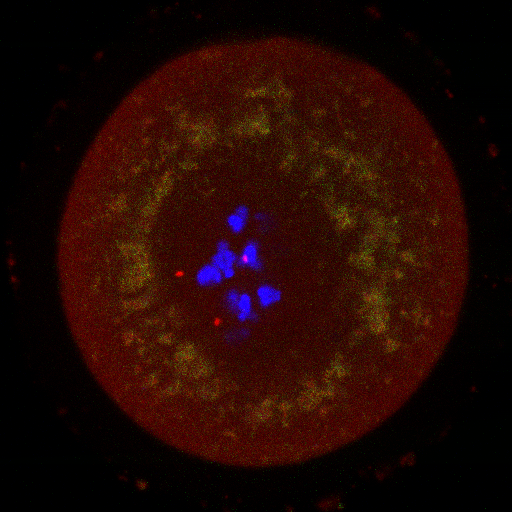

Supplement: Supplementary file 5 — Source data Fig. 1 [file 44319_2025_537_MOESM5_ESM.zip › 1C/GVBD/GVBD_MERGE.tif]

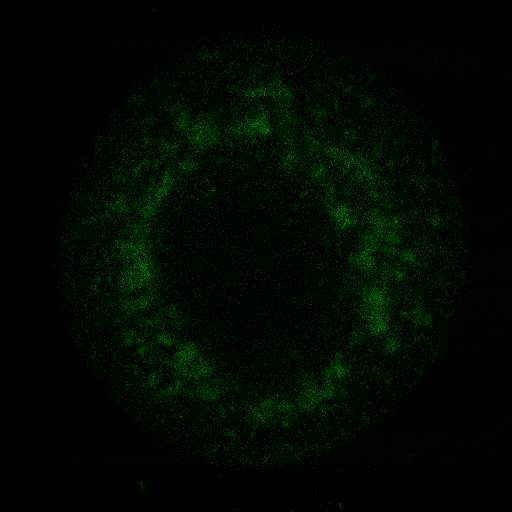

Supplement: Supplementary file 5 — Source data Fig. 1 [file 44319_2025_537_MOESM5_ESM.zip › 1C/GVBD/GVBD_TOM2O.tif]

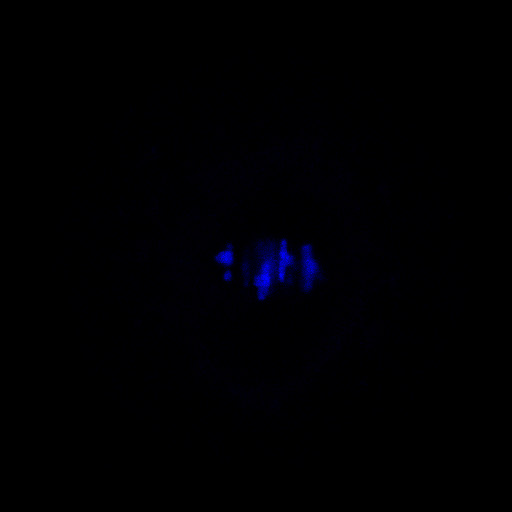

Supplement: Supplementary file 5 — Source data Fig. 1 [file 44319_2025_537_MOESM5_ESM.zip › 1C/MI/MI_DNA.tif]

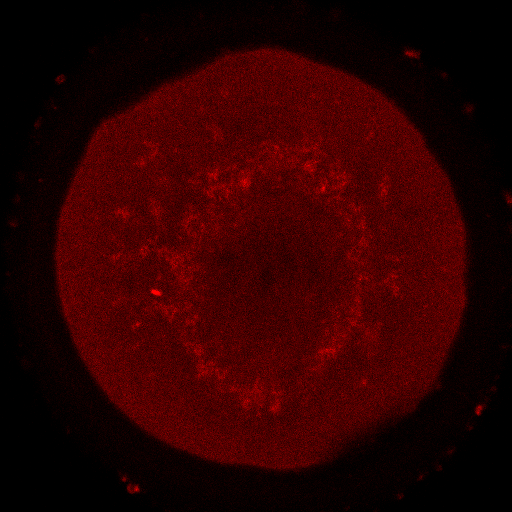

Supplement: Supplementary file 5 — Source data Fig. 1 [file 44319_2025_537_MOESM5_ESM.zip › 1C/MI/MI_KLHL8.tif]

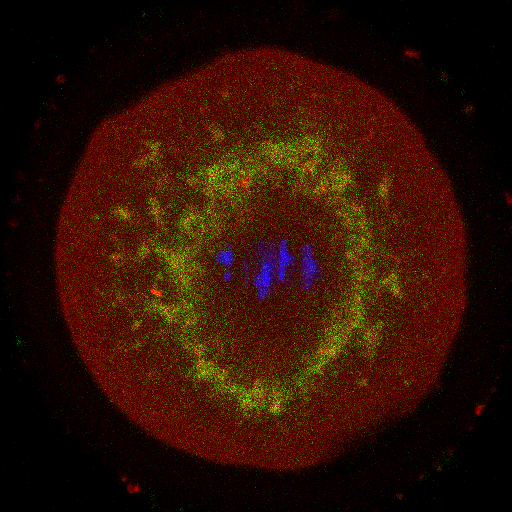

Supplement: Supplementary file 5 — Source data Fig. 1 [file 44319_2025_537_MOESM5_ESM.zip › 1C/MI/MI_MERGE.tif]

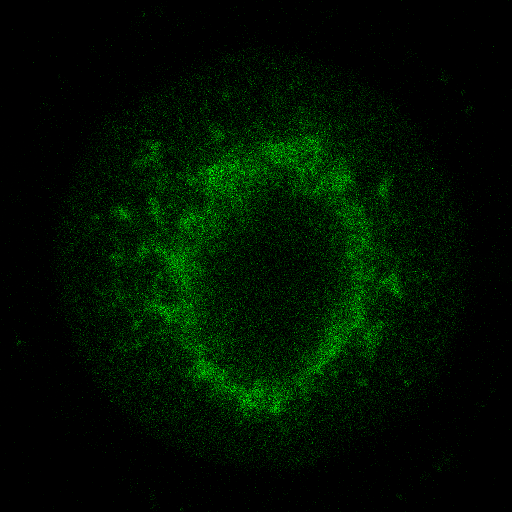

Supplement: Supplementary file 5 — Source data Fig. 1 [file 44319_2025_537_MOESM5_ESM.zip › 1C/MI/MI_TOM20.tif]

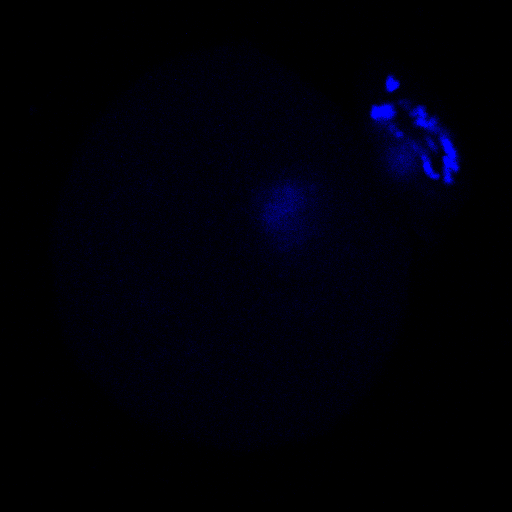

Supplement: Supplementary file 5 — Source data Fig. 1 [file 44319_2025_537_MOESM5_ESM.zip › 1C/MII/MII_DNA.tif]

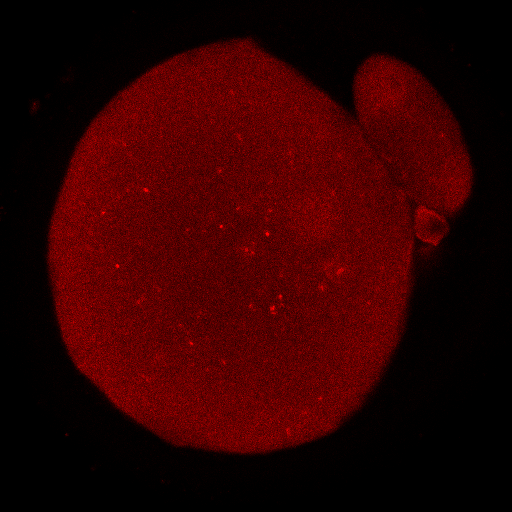

Supplement: Supplementary file 5 — Source data Fig. 1 [file 44319_2025_537_MOESM5_ESM.zip › 1C/MII/MII_KLHL8.tif]

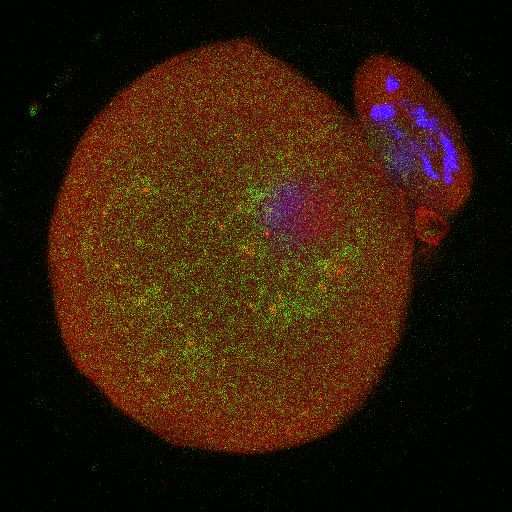

Supplement: Supplementary file 5 — Source data Fig. 1 [file 44319_2025_537_MOESM5_ESM.zip › 1C/MII/MII_MERGE.tif]

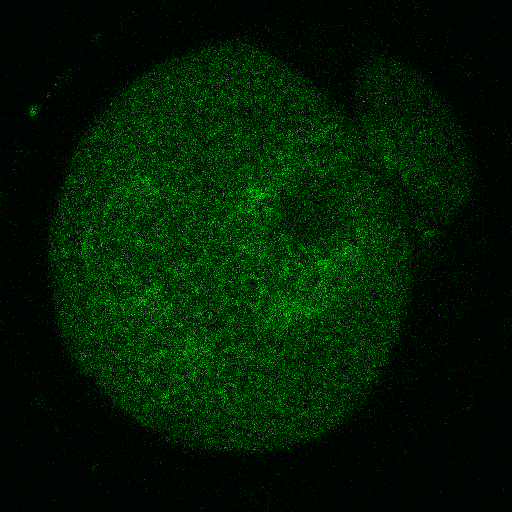

Supplement: Supplementary file 5 — Source data Fig. 1 [file 44319_2025_537_MOESM5_ESM.zip › 1C/MII/MII_TOM20.tif]

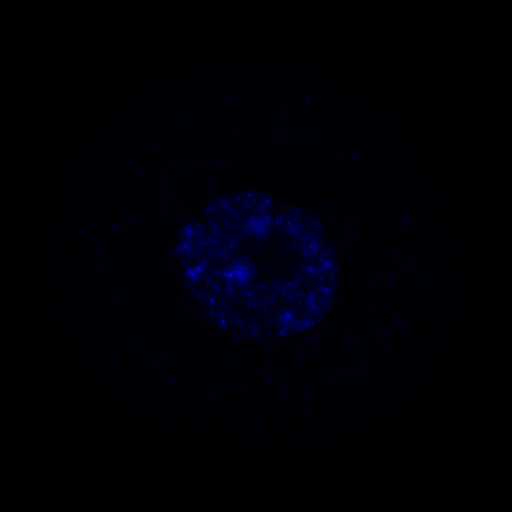

Supplement: Supplementary file 5 — Source data Fig. 1 [file 44319_2025_537_MOESM5_ESM.zip › 1C/NSN/NSN_DNA.tif]

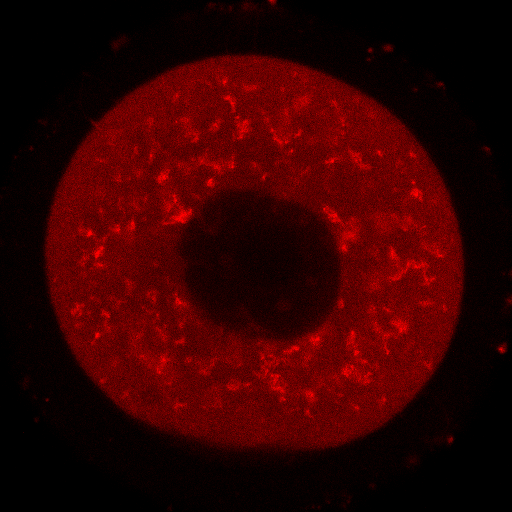

Supplement: Supplementary file 5 — Source data Fig. 1 [file 44319_2025_537_MOESM5_ESM.zip › 1C/NSN/NSN_KLHL8.tif]

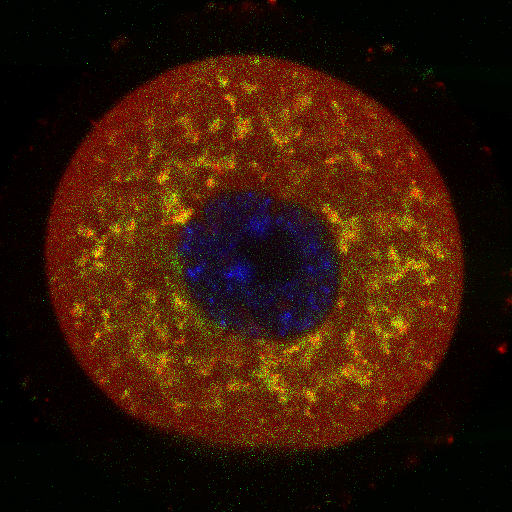

Supplement: Supplementary file 5 — Source data Fig. 1 [file 44319_2025_537_MOESM5_ESM.zip › 1C/NSN/NSN_MERGE.tif]

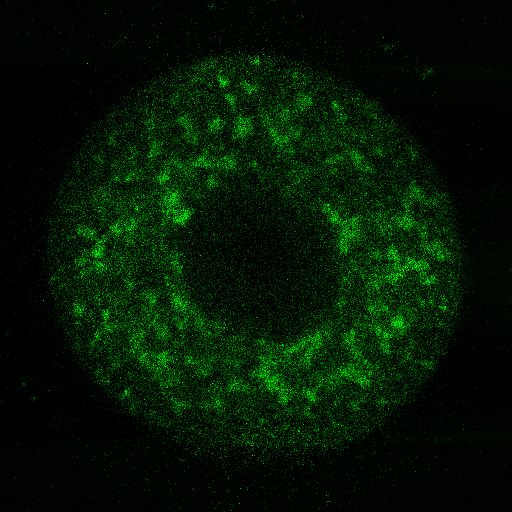

Supplement: Supplementary file 5 — Source data Fig. 1 [file 44319_2025_537_MOESM5_ESM.zip › 1C/NSN/NSN_TOM20.tif]

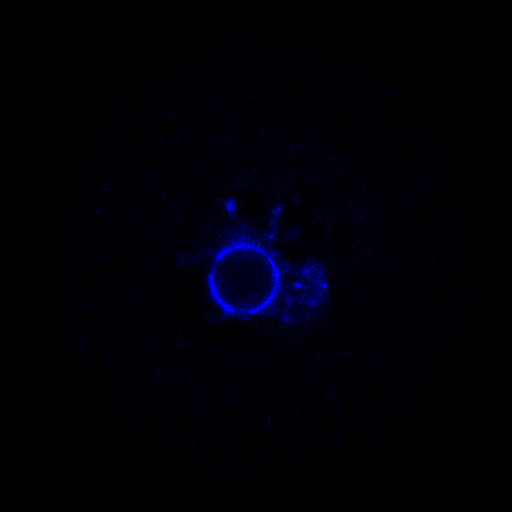

Supplement: Supplementary file 5 — Source data Fig. 1 [file 44319_2025_537_MOESM5_ESM.zip › 1C/SN/SN_DNA.tif]

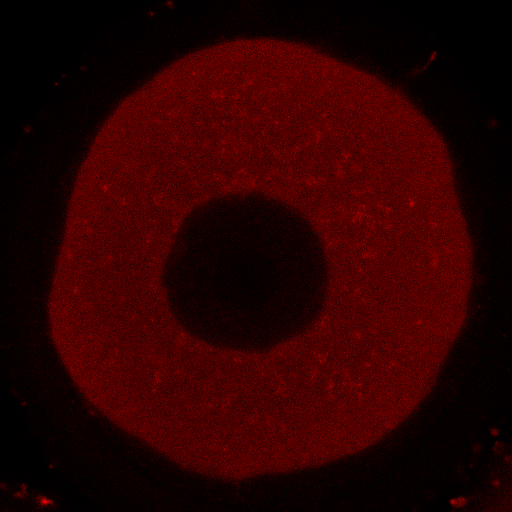

Supplement: Supplementary file 5 — Source data Fig. 1 [file 44319_2025_537_MOESM5_ESM.zip › 1C/SN/SN_KLHL8.tif]

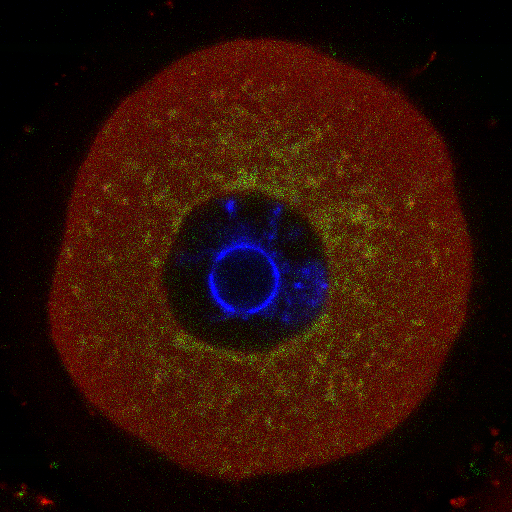

Supplement: Supplementary file 5 — Source data Fig. 1 [file 44319_2025_537_MOESM5_ESM.zip › 1C/SN/SN_MERGE.tif]

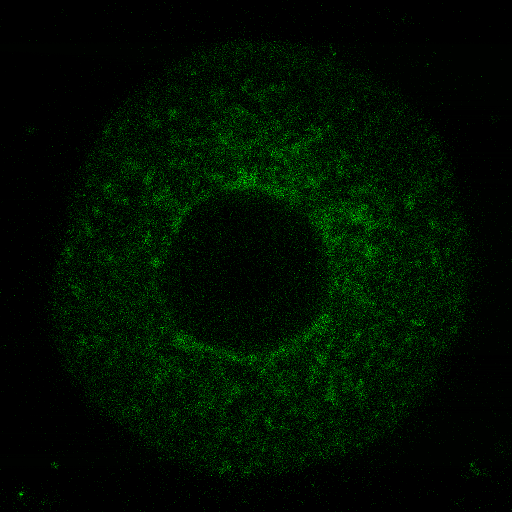

Supplement: Supplementary file 5 — Source data Fig. 1 [file 44319_2025_537_MOESM5_ESM.zip › 1C/SN/SN_TOM20.tif]

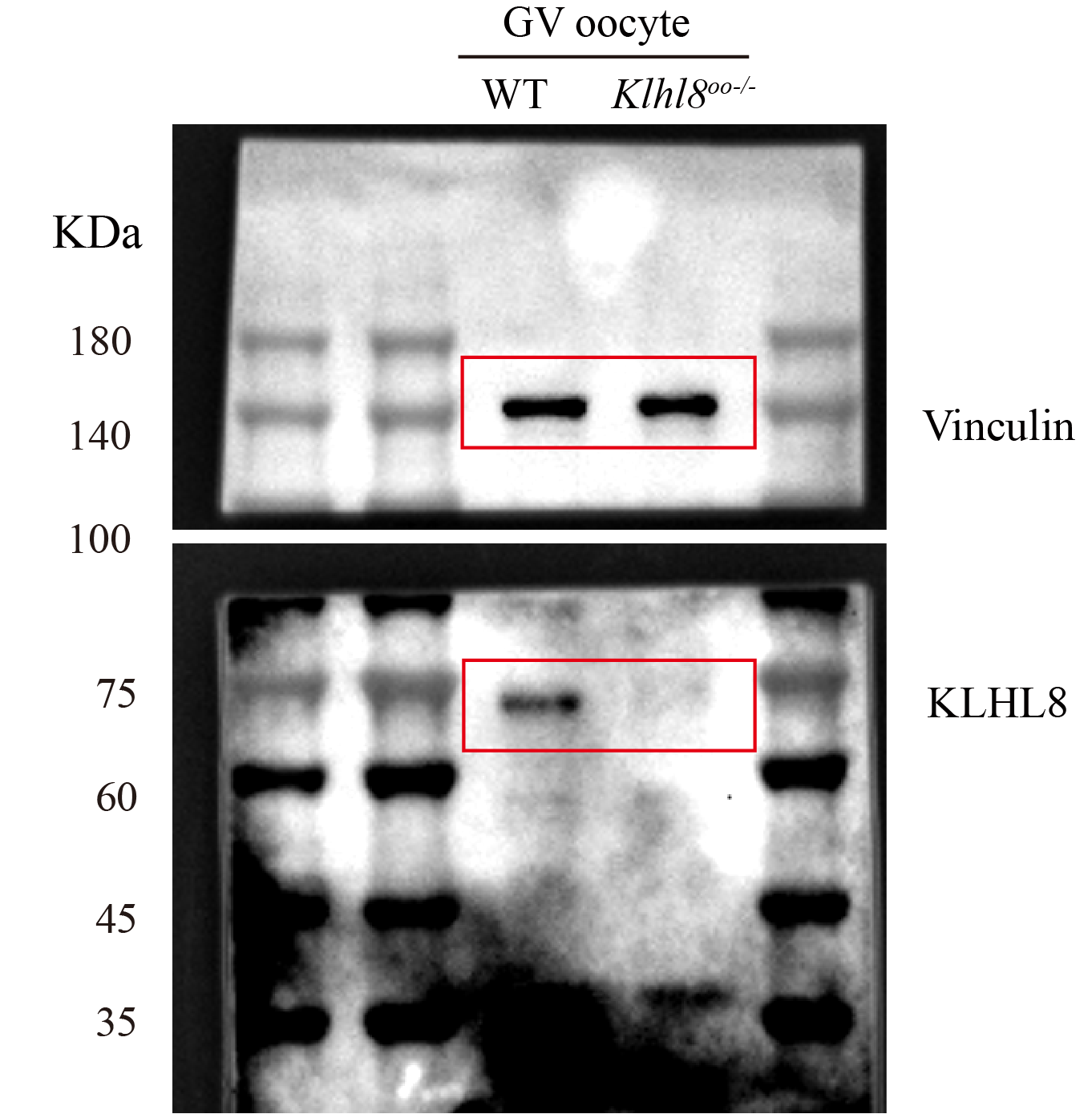

Supplement: Supplementary file 6 — Source data Fig. 2 [file 44319_2025_537_MOESM6_ESM.zip › 2C/2C.tif]

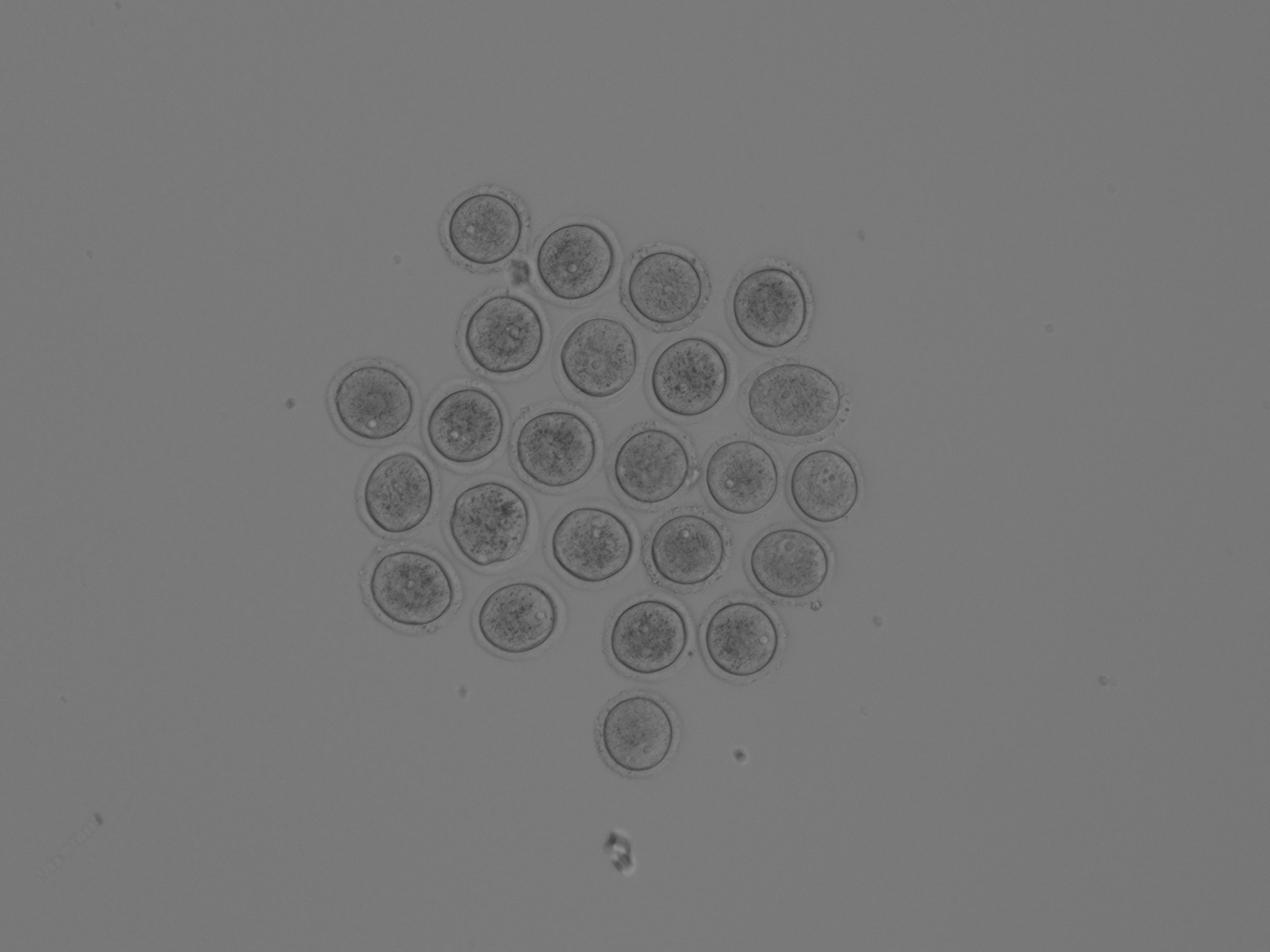

Supplement: Supplementary file 6 — Source data Fig. 2 [file 44319_2025_537_MOESM6_ESM.zip › 2E/Klhl8oo--.TIF]

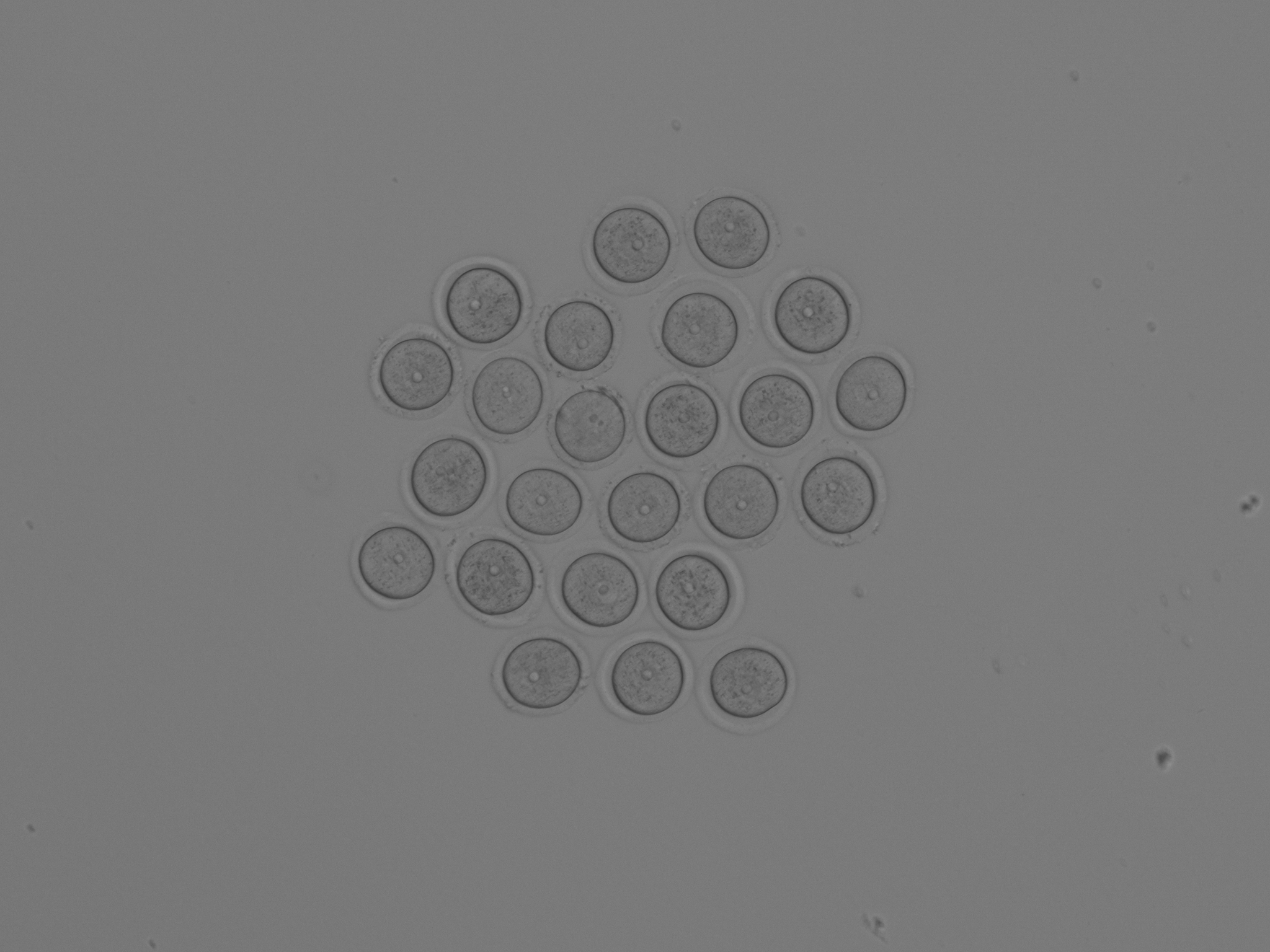

Supplement: Supplementary file 6 — Source data Fig. 2 [file 44319_2025_537_MOESM6_ESM.zip › 2E/WT.TIF]

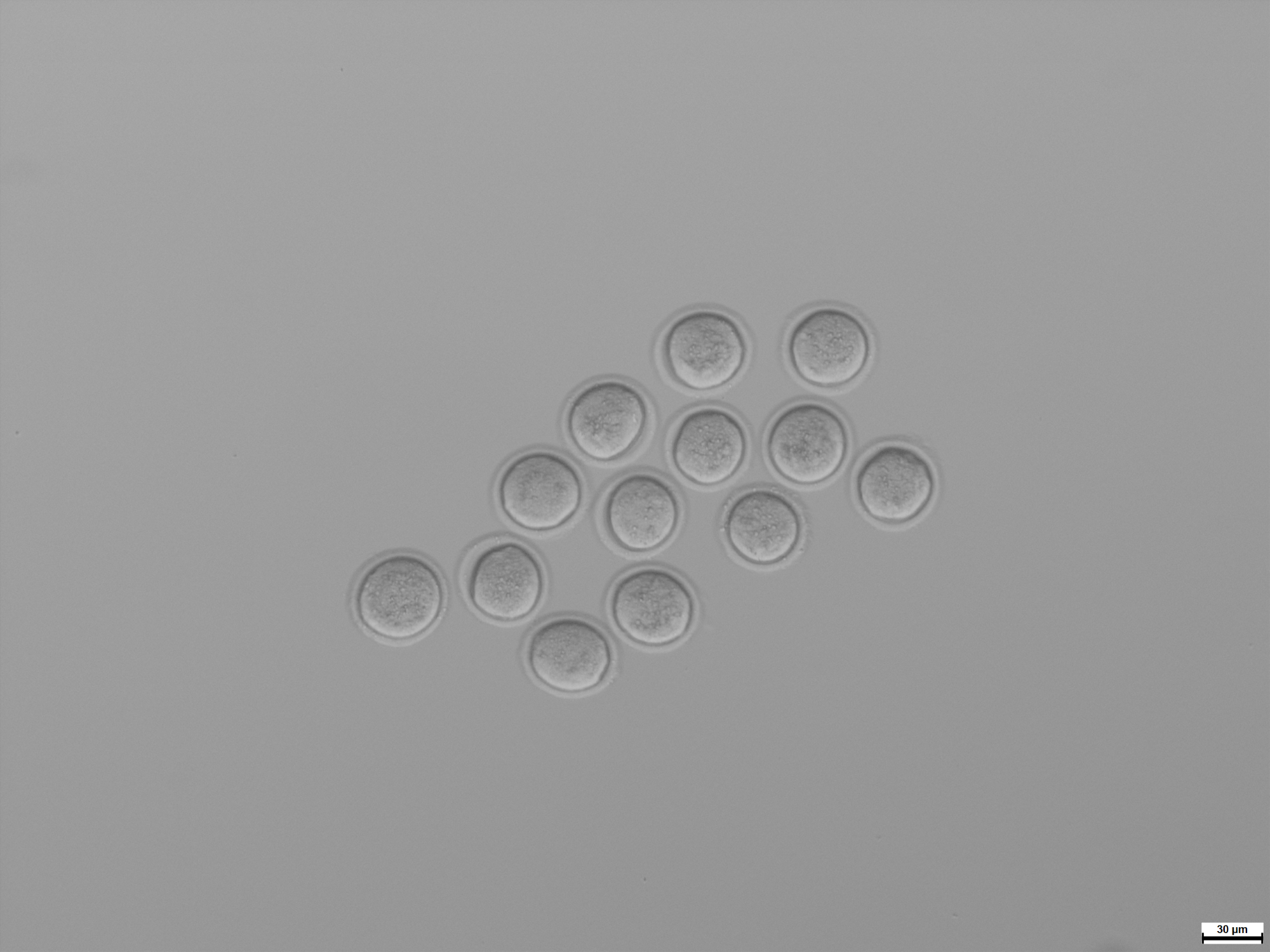

Supplement: Supplementary file 6 — Source data Fig. 2 [file 44319_2025_537_MOESM6_ESM.zip › 2G/Klhl8oo--GVBD.tif]

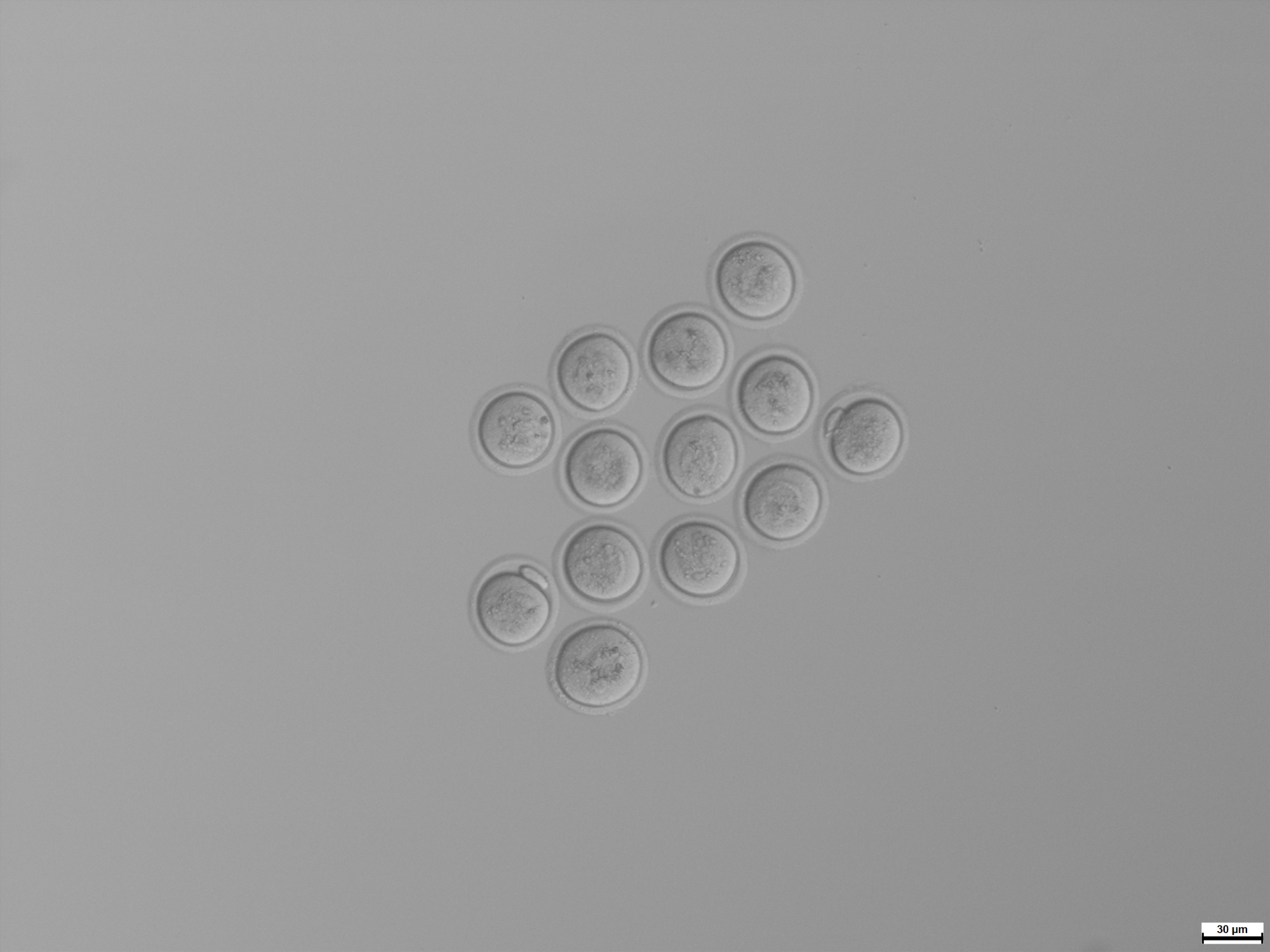

Supplement: Supplementary file 6 — Source data Fig. 2 [file 44319_2025_537_MOESM6_ESM.zip › 2G/Klhl8oo--PB1.tif]

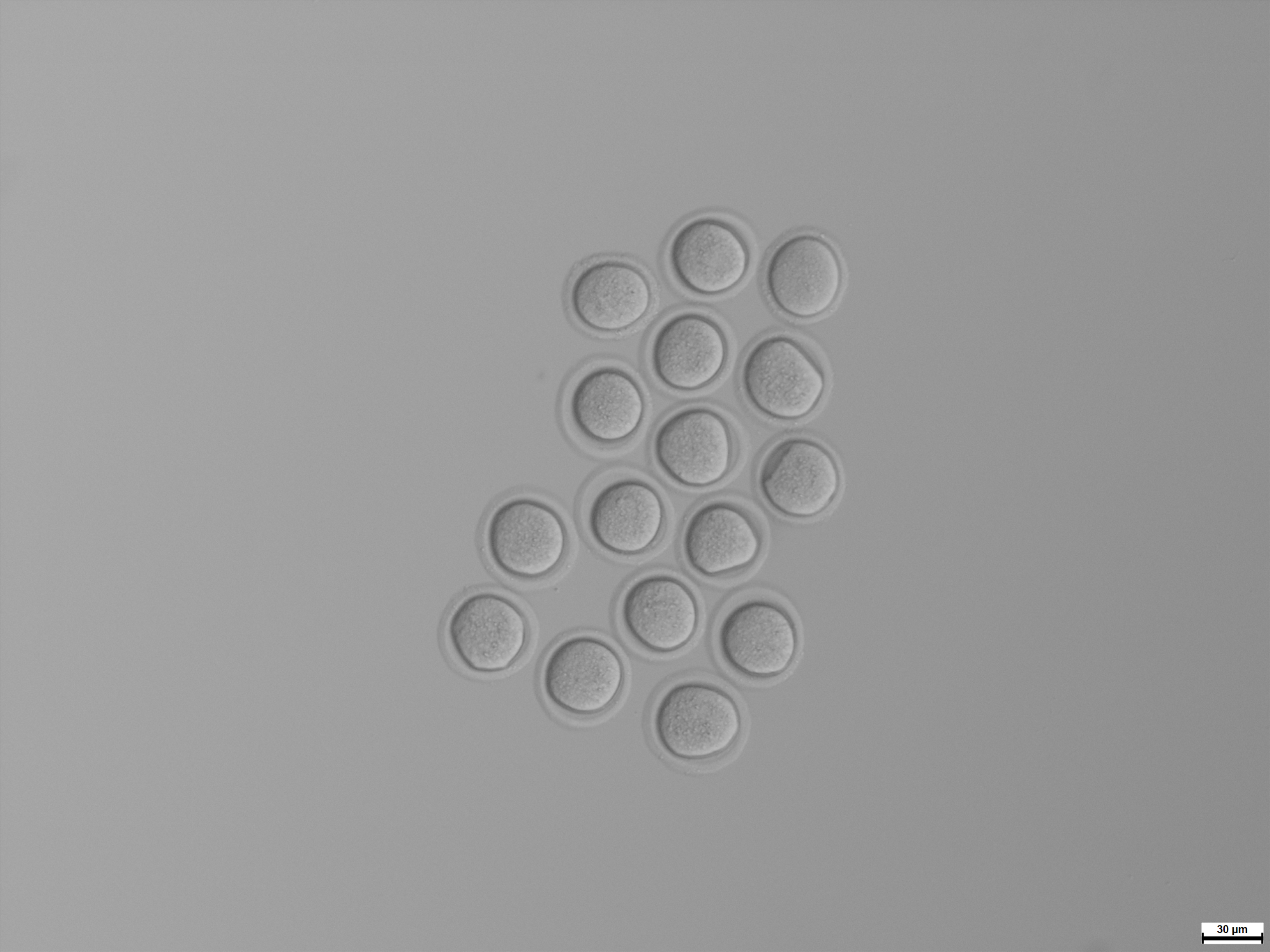

Supplement: Supplementary file 6 — Source data Fig. 2 [file 44319_2025_537_MOESM6_ESM.zip › 2G/WT-GVBD.tif]

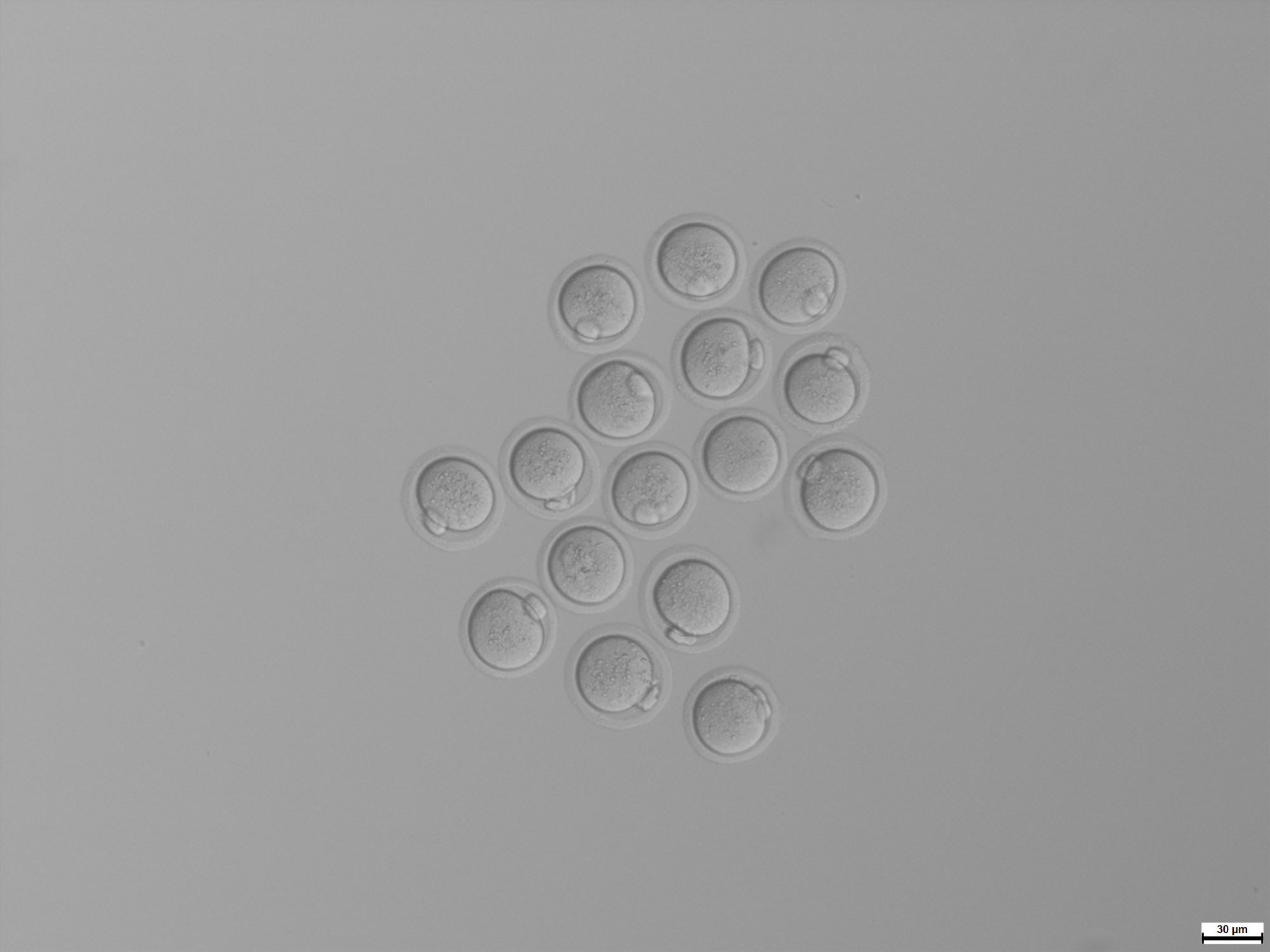

Supplement: Supplementary file 6 — Source data Fig. 2 [file 44319_2025_537_MOESM6_ESM.zip › 2G/WT-PB1.tif]

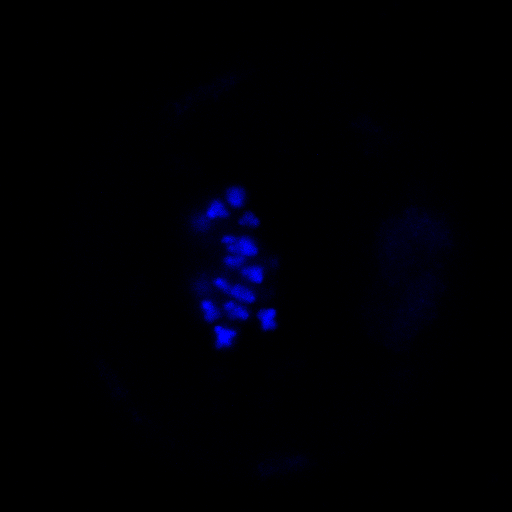

Supplement: Supplementary file 6 — Source data Fig. 2 [file 44319_2025_537_MOESM6_ESM.zip › 2I/Klhl8oo-- MI/Klhl8oo-- MI hochest.tif]

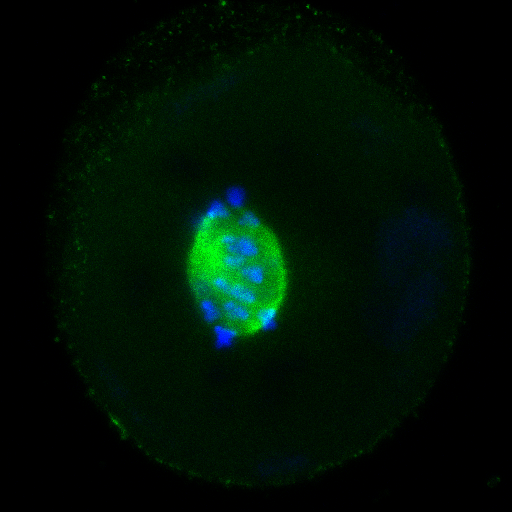

Supplement: Supplementary file 6 — Source data Fig. 2 [file 44319_2025_537_MOESM6_ESM.zip › 2I/Klhl8oo-- MI/Klhl8oo-- MI merge.tif]

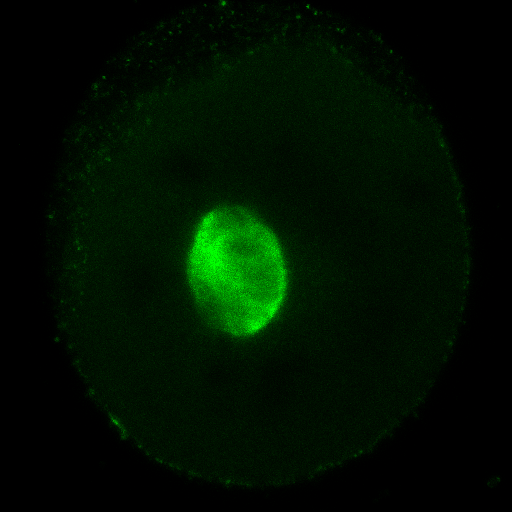

Supplement: Supplementary file 6 — Source data Fig. 2 [file 44319_2025_537_MOESM6_ESM.zip › 2I/Klhl8oo-- MI/Klhl8oo-- MI tubulin.tif]

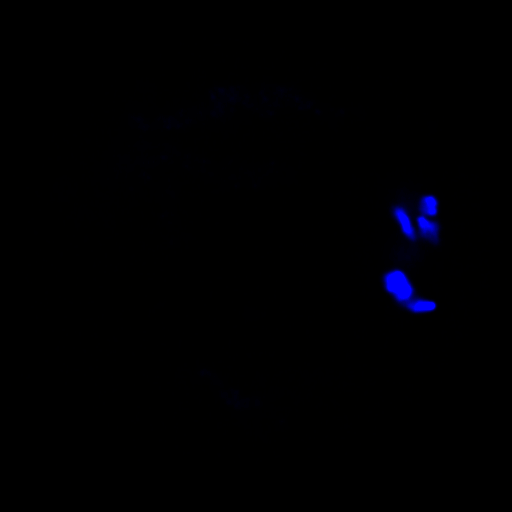

Supplement: Supplementary file 6 — Source data Fig. 2 [file 44319_2025_537_MOESM6_ESM.zip › 2I/Klhl8oo-- MII/Klhl8oo-- MII hochest.tif]

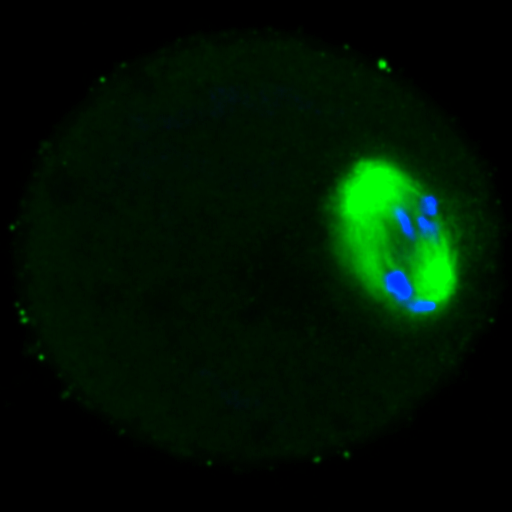

Supplement: Supplementary file 6 — Source data Fig. 2 [file 44319_2025_537_MOESM6_ESM.zip › 2I/Klhl8oo-- MII/Klhl8oo-- MII MERGE.tif]

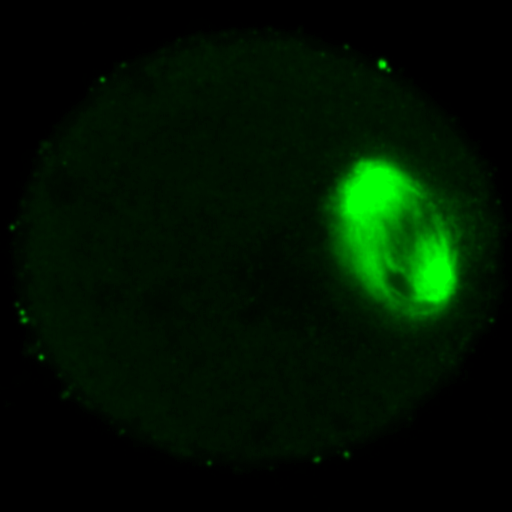

Supplement: Supplementary file 6 — Source data Fig. 2 [file 44319_2025_537_MOESM6_ESM.zip › 2I/Klhl8oo-- MII/Klhl8oo-- MII tubulin.tif]

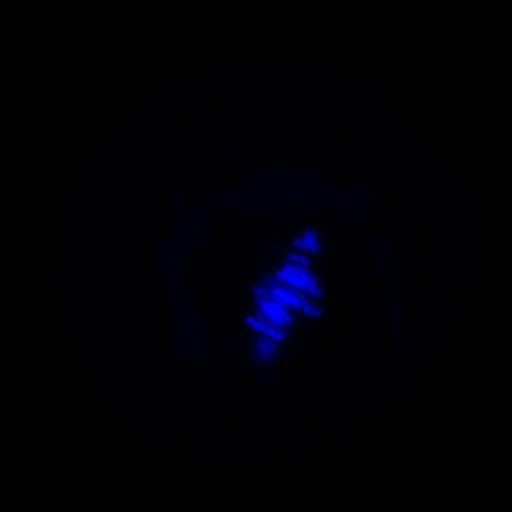

Supplement: Supplementary file 6 — Source data Fig. 2 [file 44319_2025_537_MOESM6_ESM.zip › 2I/WT MI/WT MI_hochest.tif]

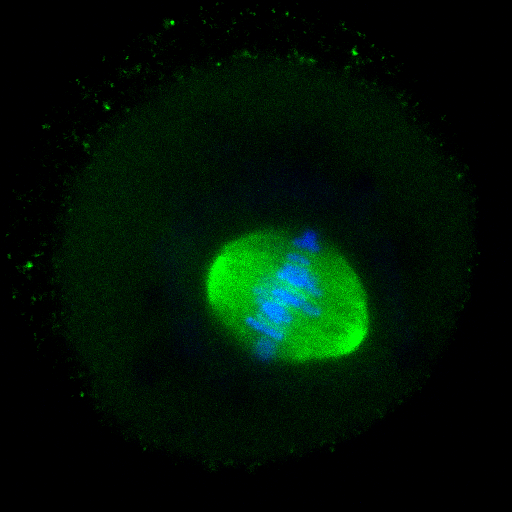

Supplement: Supplementary file 6 — Source data Fig. 2 [file 44319_2025_537_MOESM6_ESM.zip › 2I/WT MI/WT MI_merge.tif]

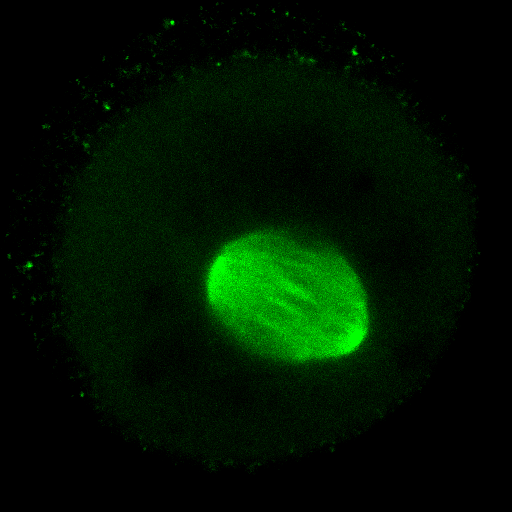

Supplement: Supplementary file 6 — Source data Fig. 2 [file 44319_2025_537_MOESM6_ESM.zip › 2I/WT MI/WT MI_tubulin.tif]

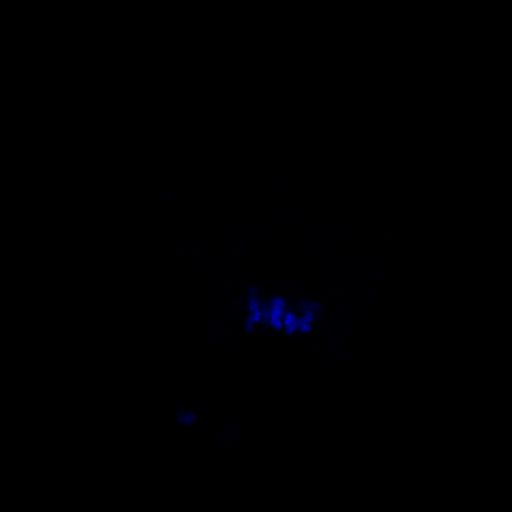

Supplement: Supplementary file 6 — Source data Fig. 2 [file 44319_2025_537_MOESM6_ESM.zip › 2I/WT MII/WT MII_hochest.tif]

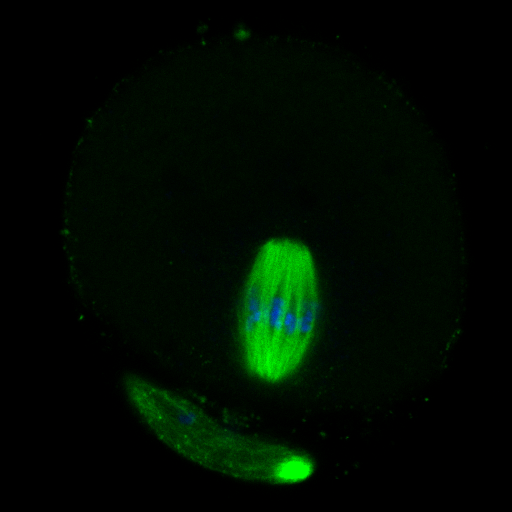

Supplement: Supplementary file 6 — Source data Fig. 2 [file 44319_2025_537_MOESM6_ESM.zip › 2I/WT MII/WT MII_merge.tif]

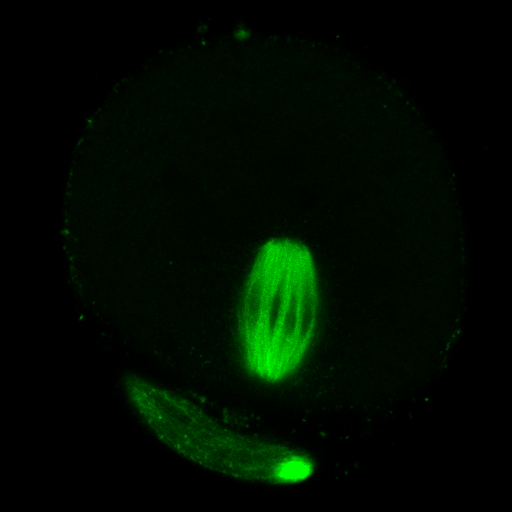

Supplement: Supplementary file 6 — Source data Fig. 2 [file 44319_2025_537_MOESM6_ESM.zip › 2I/WT MII/WT MII_tubulin.tif]

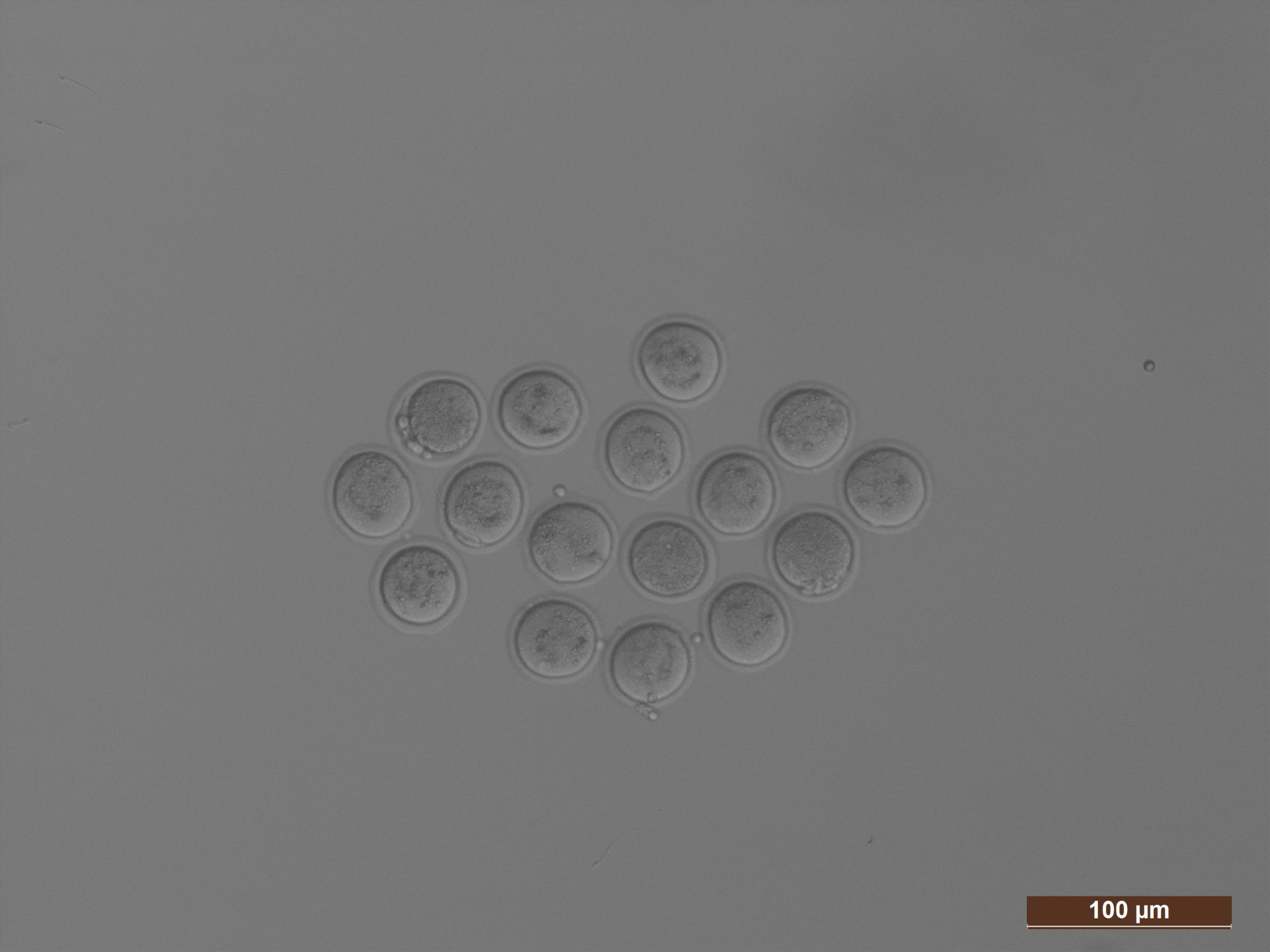

Supplement: Supplementary file 6 — Source data Fig. 2 [file 44319_2025_537_MOESM6_ESM.zip › 2L/Klhl8oo--2PN.tif]

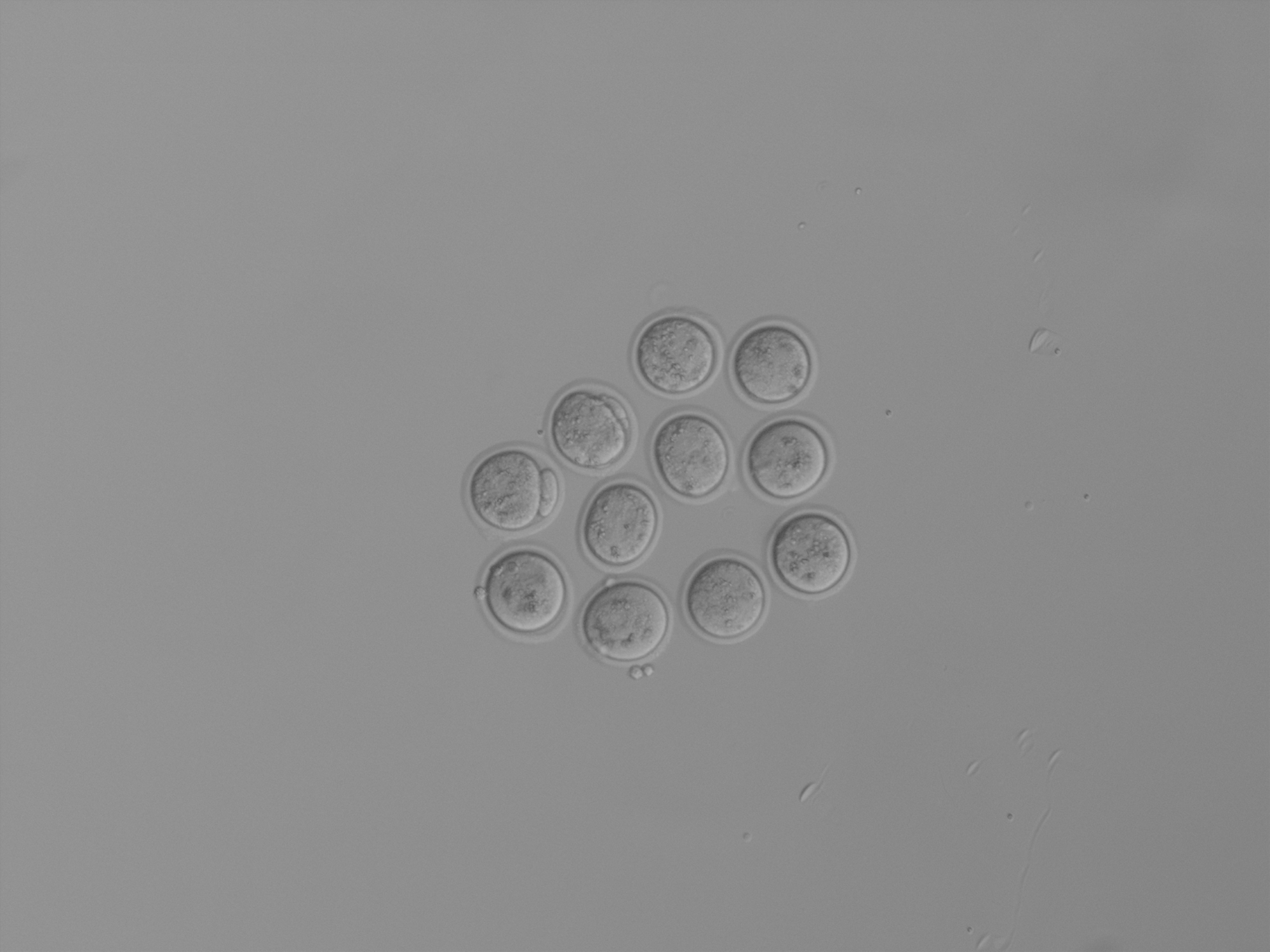

Supplement: Supplementary file 6 — Source data Fig. 2 [file 44319_2025_537_MOESM6_ESM.zip › 2L/Klhl8oo--MII.tif]

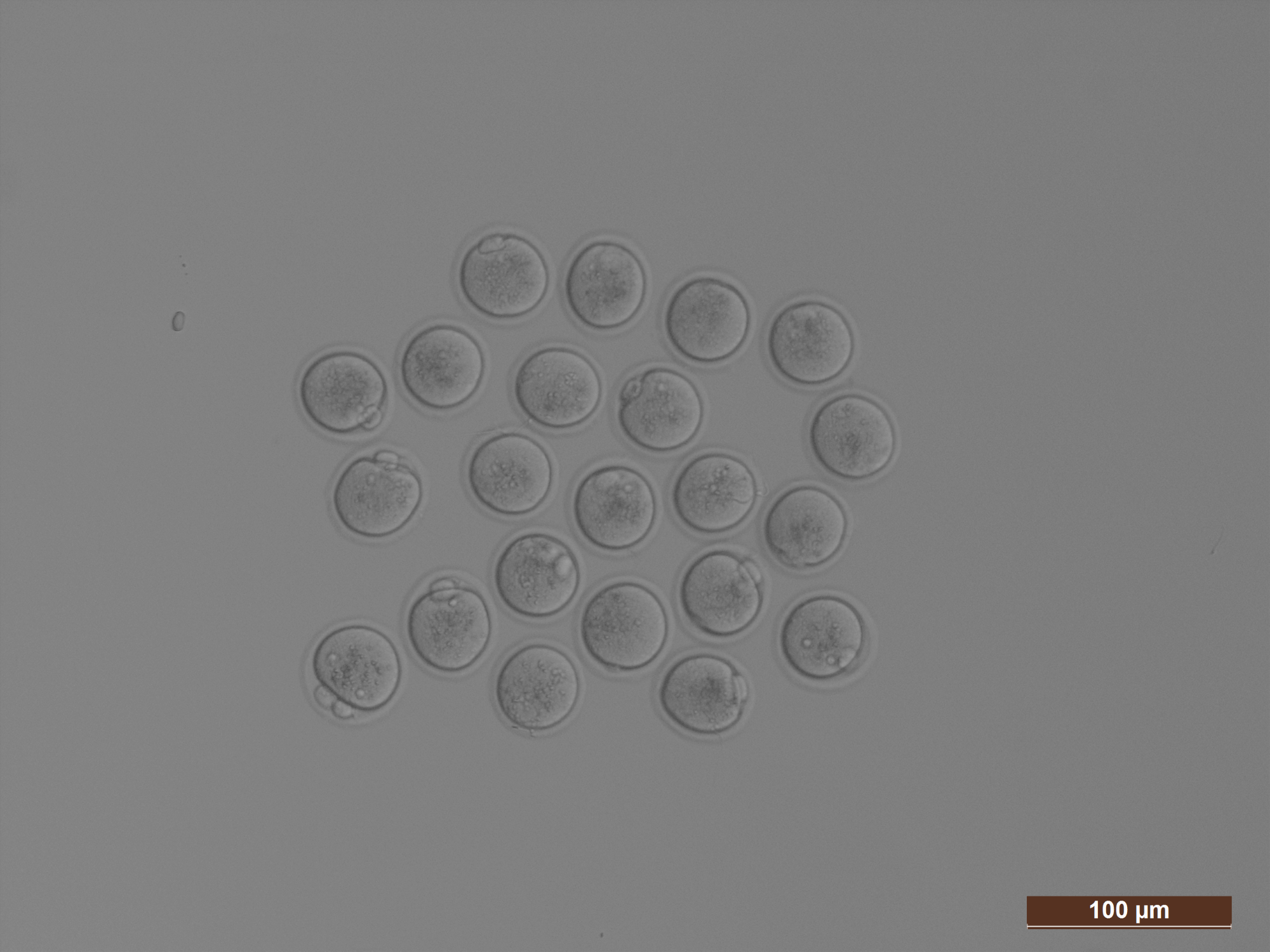

Supplement: Supplementary file 6 — Source data Fig. 2 [file 44319_2025_537_MOESM6_ESM.zip › 2L/WT-2PN.tif]

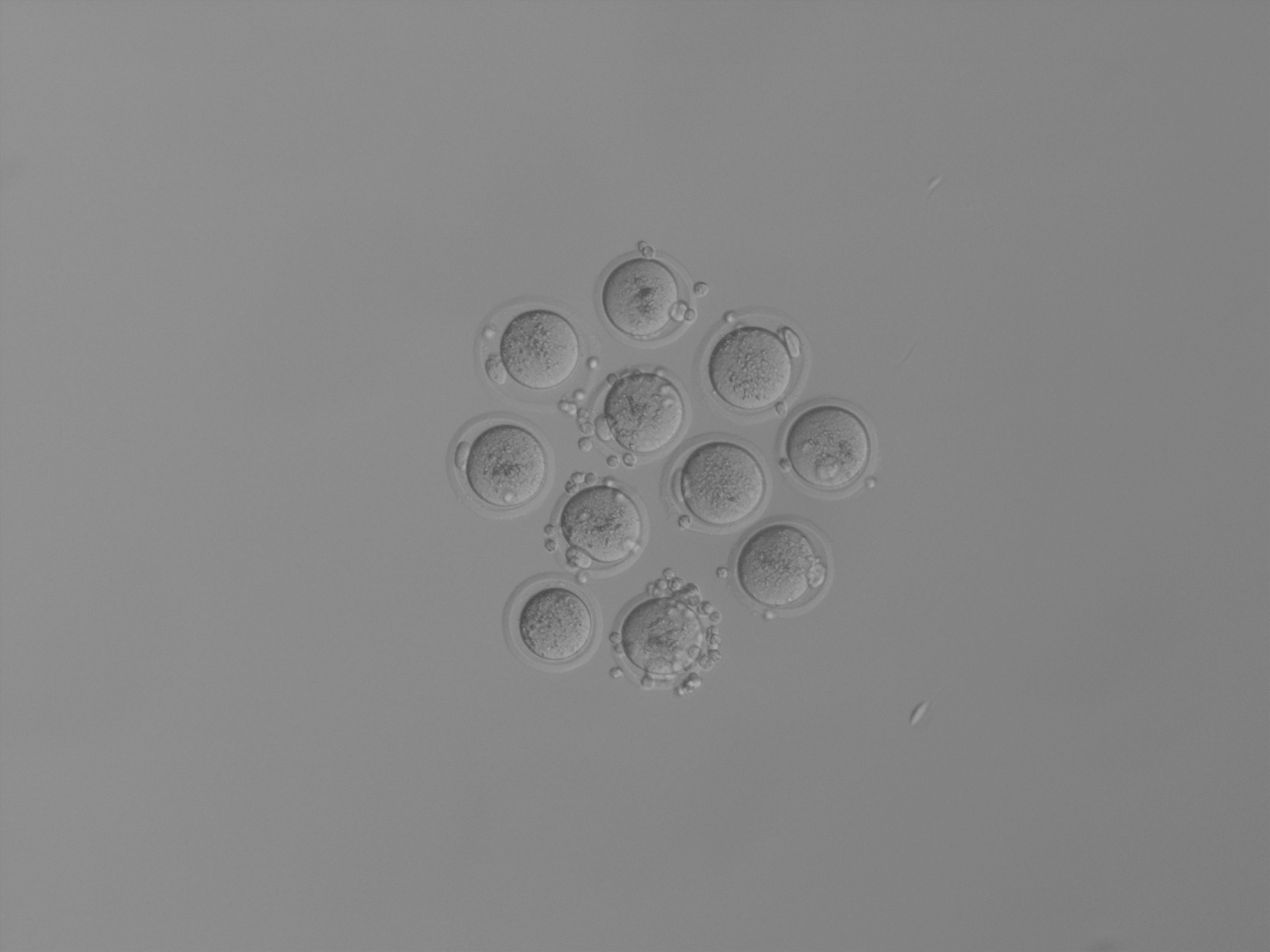

Supplement: Supplementary file 6 — Source data Fig. 2 [file 44319_2025_537_MOESM6_ESM.zip › 2L/WT-MII.tif]

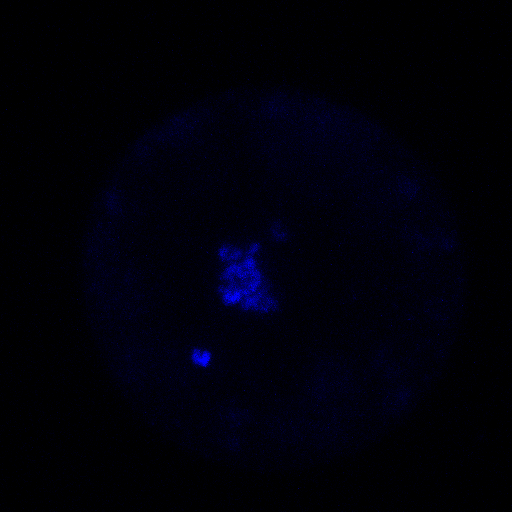

Supplement: Supplementary file 6 — Source data Fig. 2 [file 44319_2025_537_MOESM6_ESM.zip › 2N/Klhl8oo--MII-1/Klhl8oo--MII-1-DNA.tif]

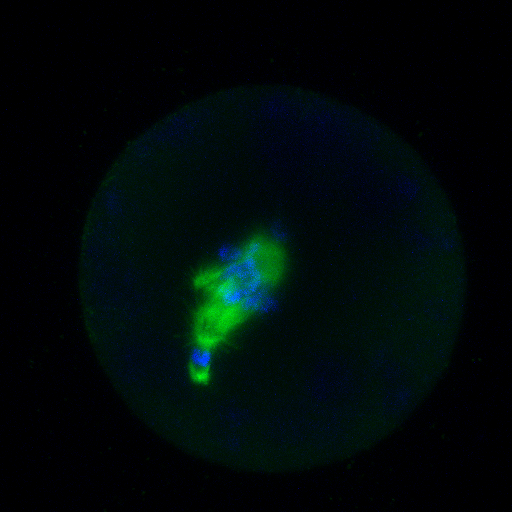

Supplement: Supplementary file 6 — Source data Fig. 2 [file 44319_2025_537_MOESM6_ESM.zip › 2N/Klhl8oo--MII-1/Klhl8oo--MII-1-MERGE.tif]

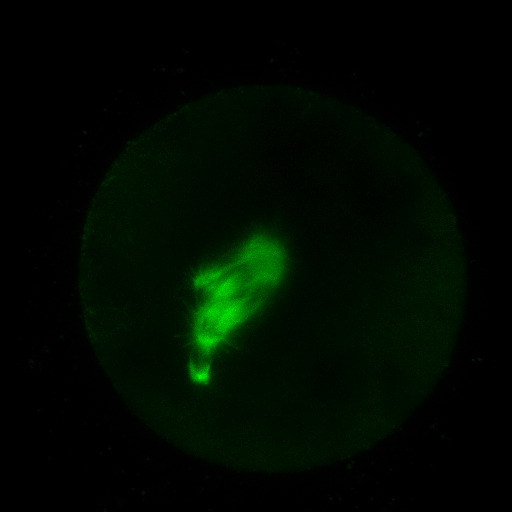

Supplement: Supplementary file 6 — Source data Fig. 2 [file 44319_2025_537_MOESM6_ESM.zip › 2N/Klhl8oo--MII-1/Klhl8oo--MII-1-Tubulin.tif]

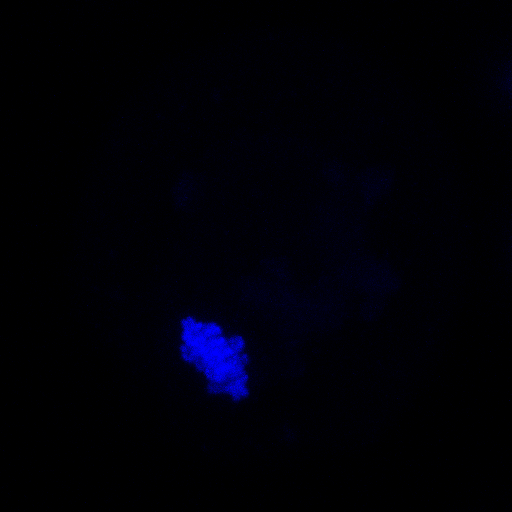

Supplement: Supplementary file 6 — Source data Fig. 2 [file 44319_2025_537_MOESM6_ESM.zip › 2N/Klhl8oo--MII-2/Klhl8oo--MII-2-hochest.tif]

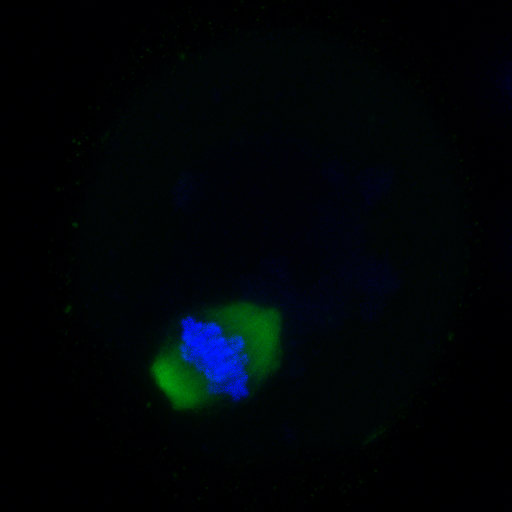

Supplement: Supplementary file 6 — Source data Fig. 2 [file 44319_2025_537_MOESM6_ESM.zip › 2N/Klhl8oo--MII-2/Klhl8oo--MII-2-merge.tif]

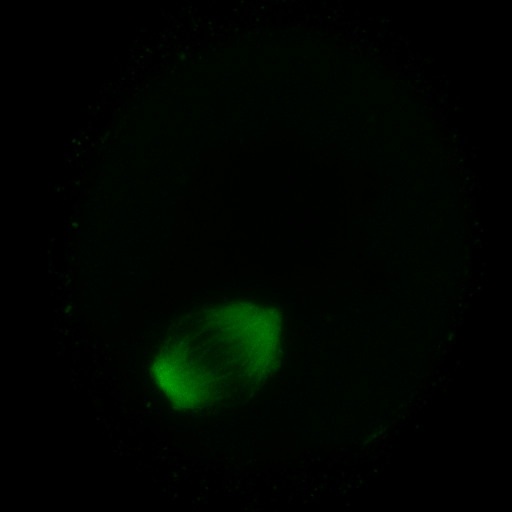

Supplement: Supplementary file 6 — Source data Fig. 2 [file 44319_2025_537_MOESM6_ESM.zip › 2N/Klhl8oo--MII-2/Klhl8oo--MII-2-tubulin.tif]

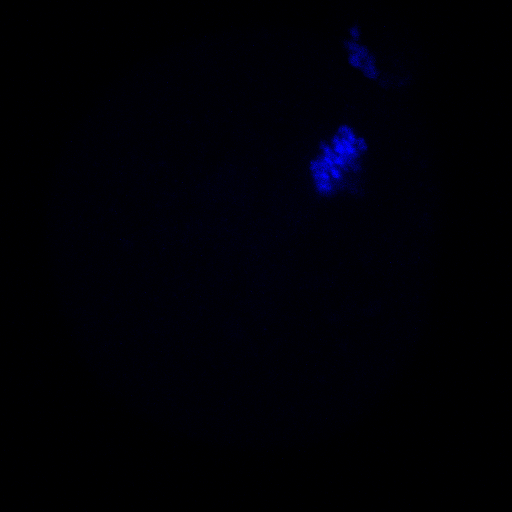

Supplement: Supplementary file 6 — Source data Fig. 2 [file 44319_2025_537_MOESM6_ESM.zip › 2N/WT-MII-1/WT-MII-1-hochest.tif]

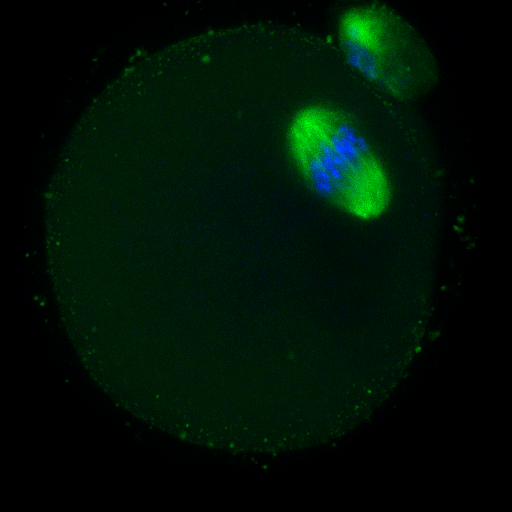

Supplement: Supplementary file 6 — Source data Fig. 2 [file 44319_2025_537_MOESM6_ESM.zip › 2N/WT-MII-1/WT-MII-1-merge.tif]

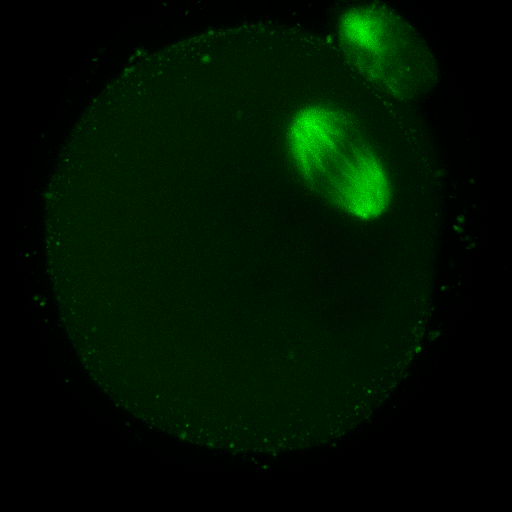

Supplement: Supplementary file 6 — Source data Fig. 2 [file 44319_2025_537_MOESM6_ESM.zip › 2N/WT-MII-1/WT-MII-1-tubulin.tif]

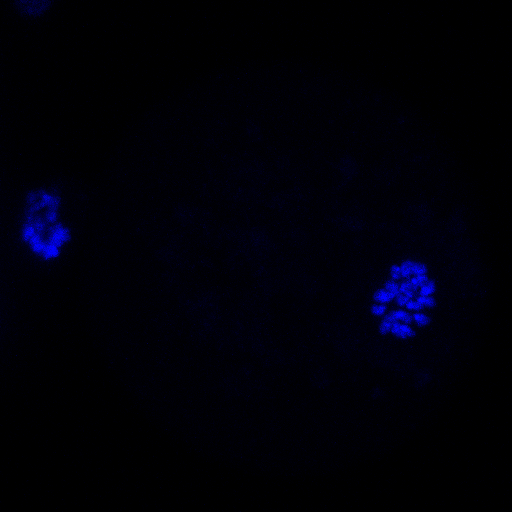

Supplement: Supplementary file 6 — Source data Fig. 2 [file 44319_2025_537_MOESM6_ESM.zip › 2N/WT-MII-2/WT-MII-2-hochest.tif]

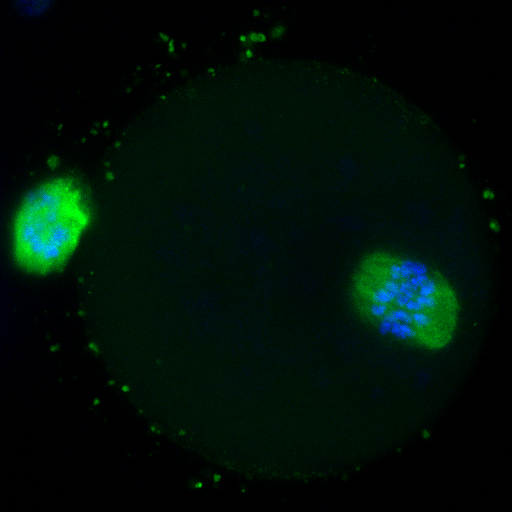

Supplement: Supplementary file 6 — Source data Fig. 2 [file 44319_2025_537_MOESM6_ESM.zip › 2N/WT-MII-2/WT-MII-2-merge.tif]

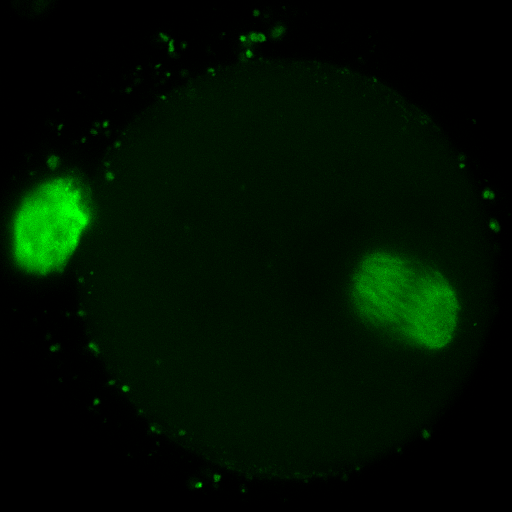

Supplement: Supplementary file 6 — Source data Fig. 2 [file 44319_2025_537_MOESM6_ESM.zip › 2N/WT-MII-2/WT-MII-2-tubulin.tif]

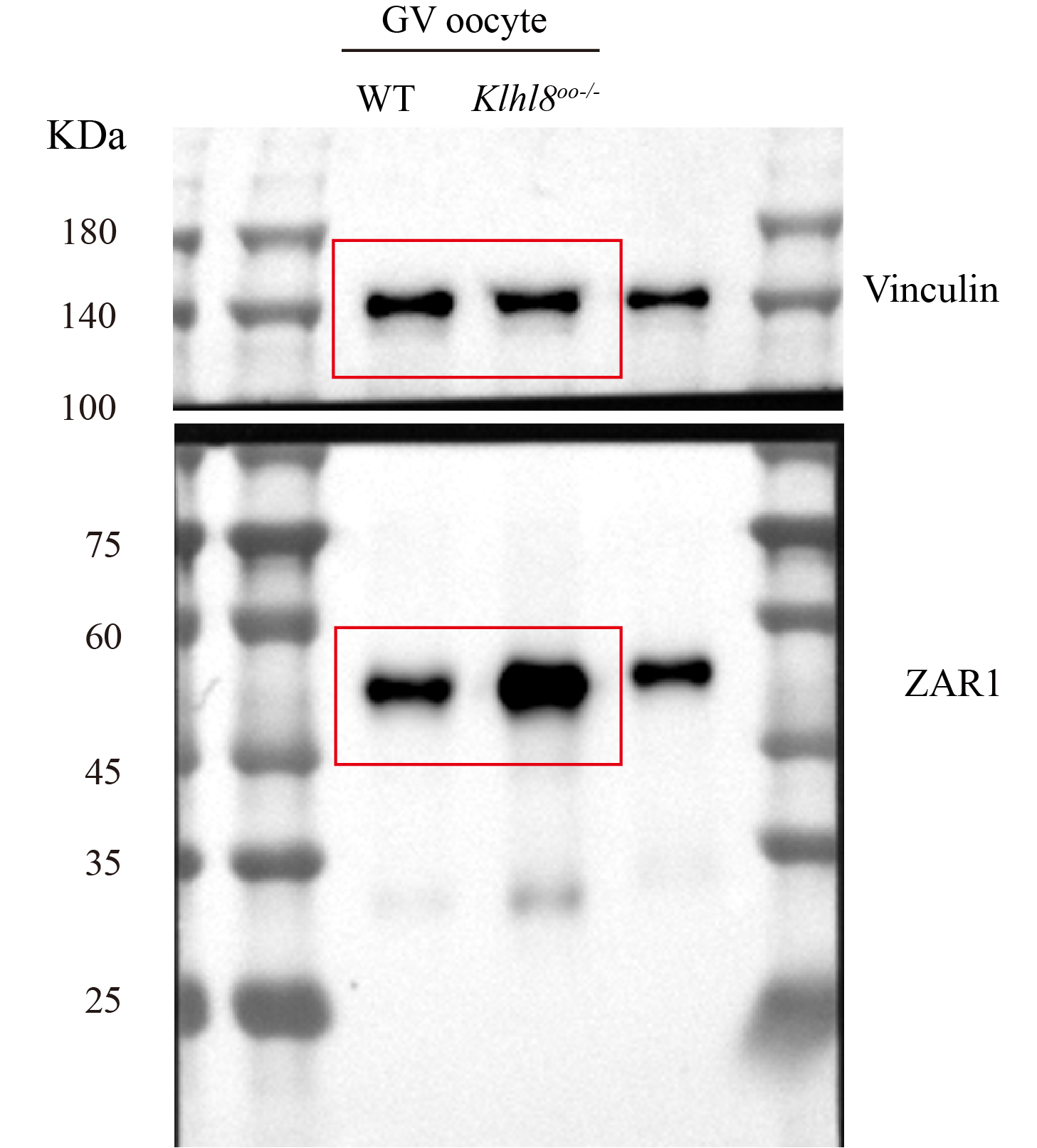

Supplement: Supplementary file 7 — Source data Fig. 3 [file 44319_2025_537_MOESM7_ESM.zip › 3D/3D.tif]

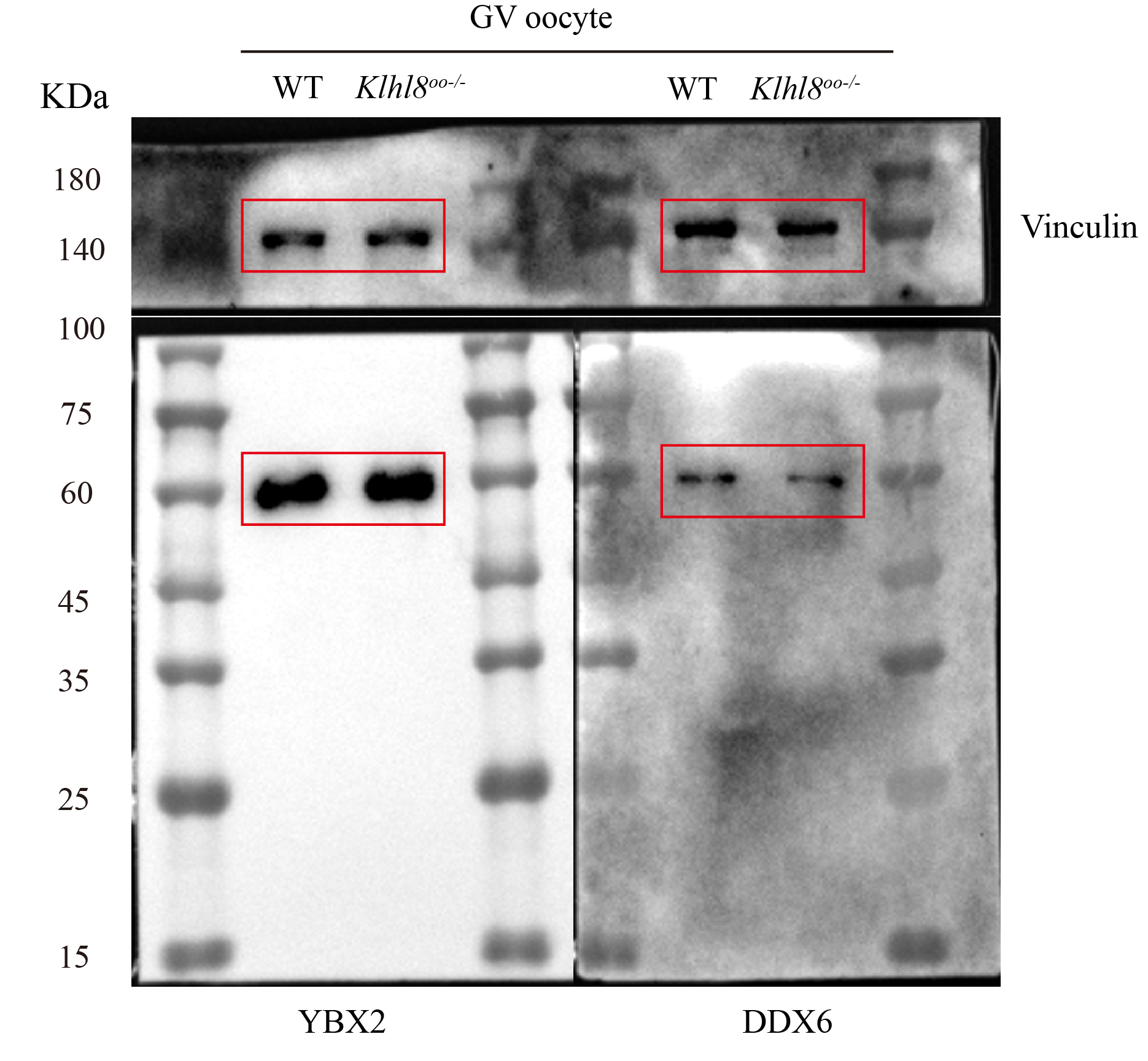

Supplement: Supplementary file 7 — Source data Fig. 3 [file 44319_2025_537_MOESM7_ESM.zip › 3G/3G.tif]

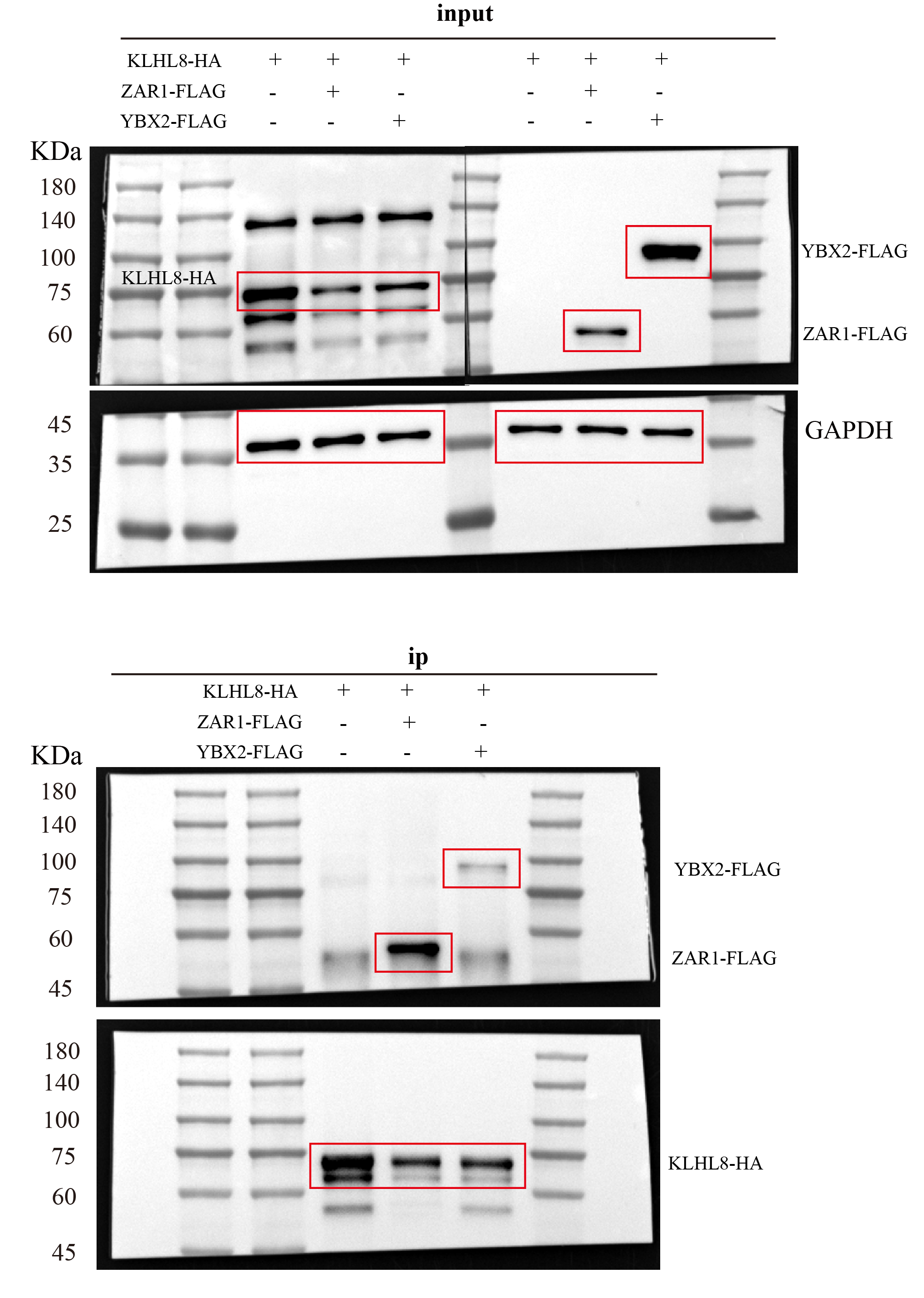

Supplement: Supplementary file 8 — Source data Fig. 4 [file 44319_2025_537_MOESM8_ESM.zip › 4A/4A.tif]

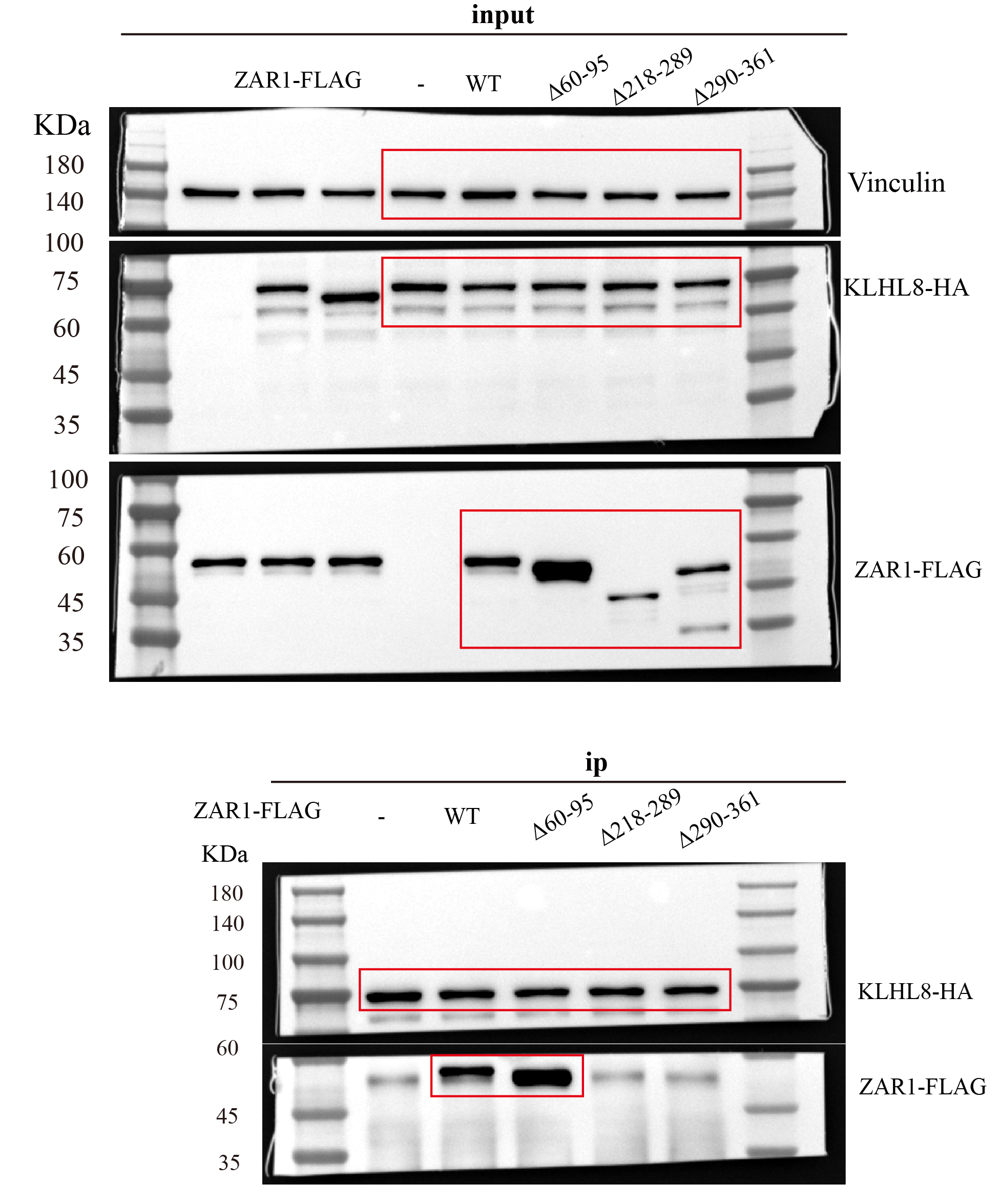

Supplement: Supplementary file 8 — Source data Fig. 4 [file 44319_2025_537_MOESM8_ESM.zip › 4B/4B.tif]

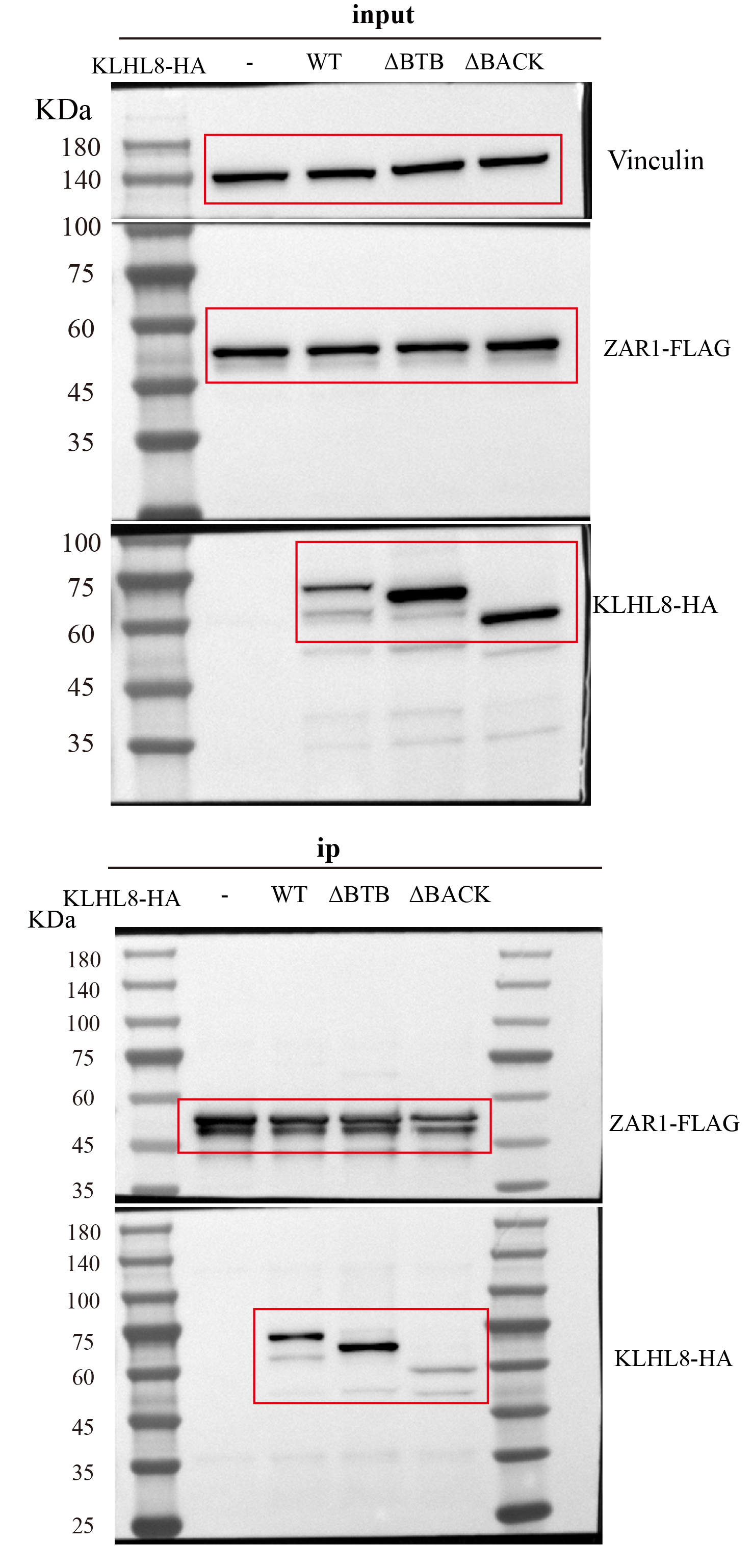

Supplement: Supplementary file 8 — Source data Fig. 4 [file 44319_2025_537_MOESM8_ESM.zip › 4C/4C.tif]

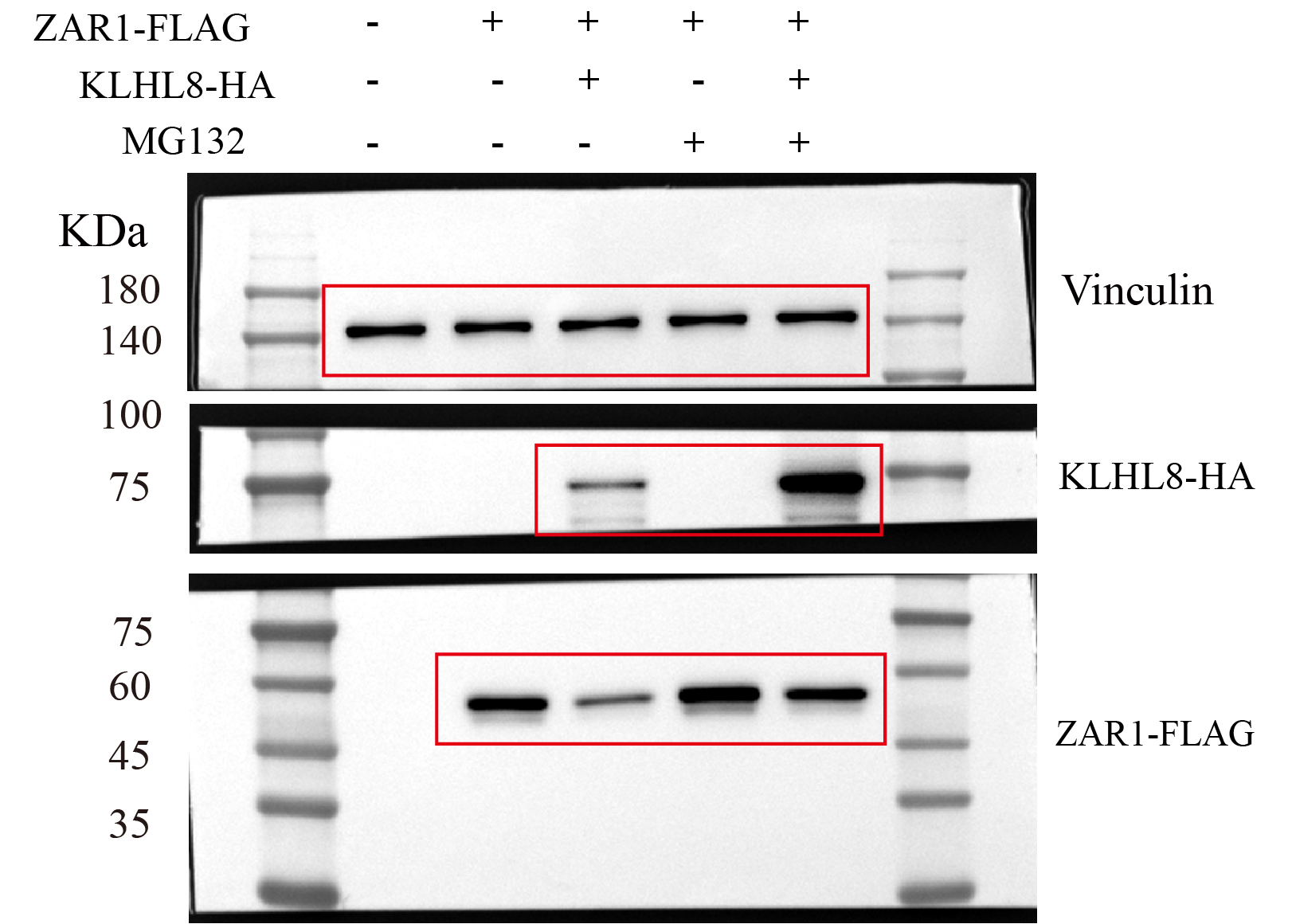

Supplement: Supplementary file 8 — Source data Fig. 4 [file 44319_2025_537_MOESM8_ESM.zip › 4D/4D.tif]

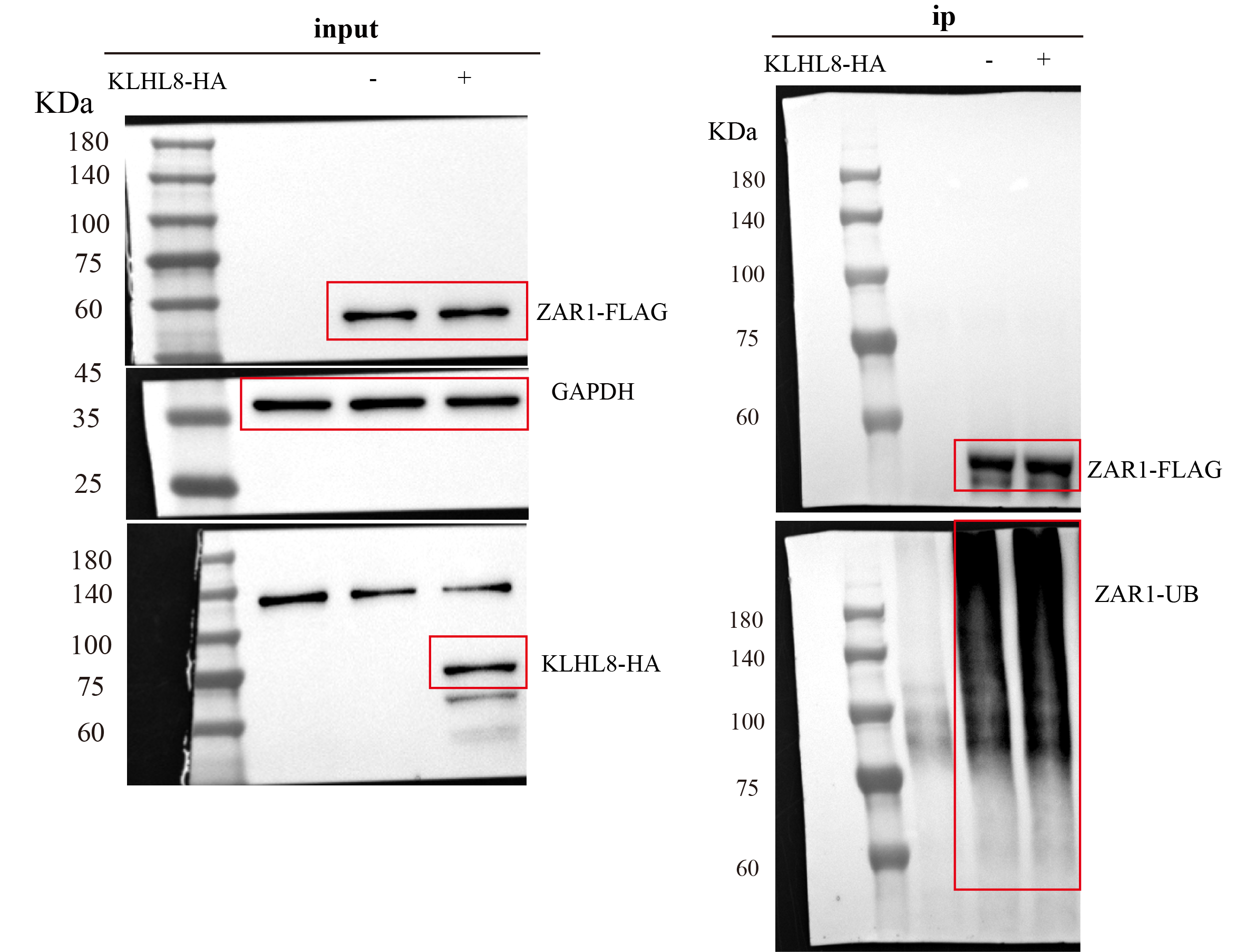

Supplement: Supplementary file 8 — Source data Fig. 4 [file 44319_2025_537_MOESM8_ESM.zip › 4F/4F.tif]

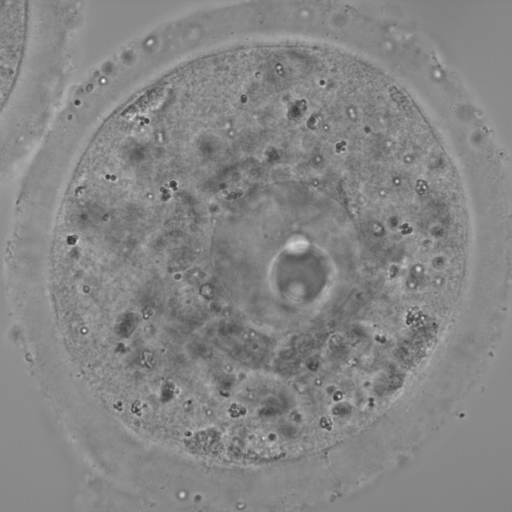

Supplement: Supplementary file 9 — Source data Fig. 5 [file 44319_2025_537_MOESM9_ESM.zip › 5A/Klhl8oo--/Klhl8oo--_bright.tif]

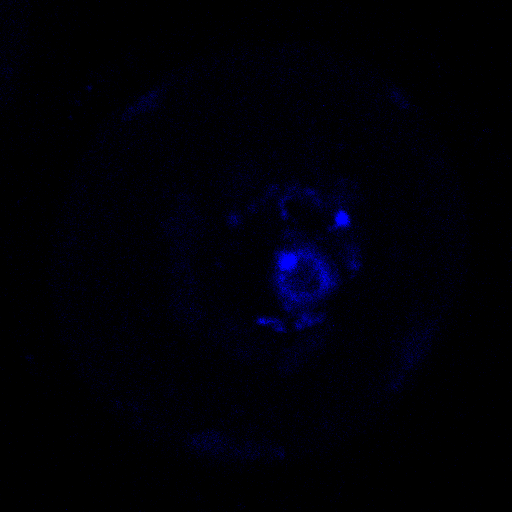

Supplement: Supplementary file 9 — Source data Fig. 5 [file 44319_2025_537_MOESM9_ESM.zip › 5A/Klhl8oo--/Klhl8oo--_hoechst.tif]

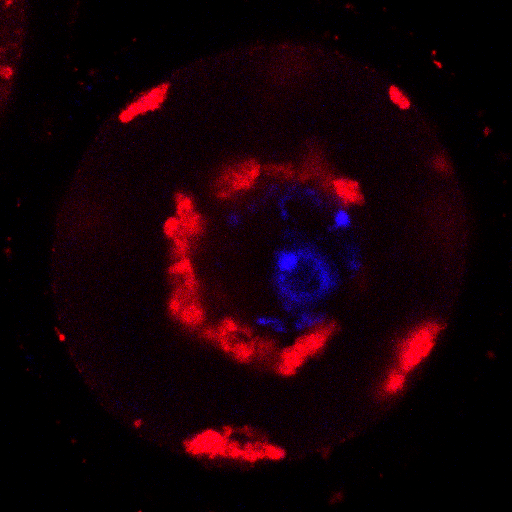

Supplement: Supplementary file 9 — Source data Fig. 5 [file 44319_2025_537_MOESM9_ESM.zip › 5A/Klhl8oo--/Klhl8oo--_merge.tif]

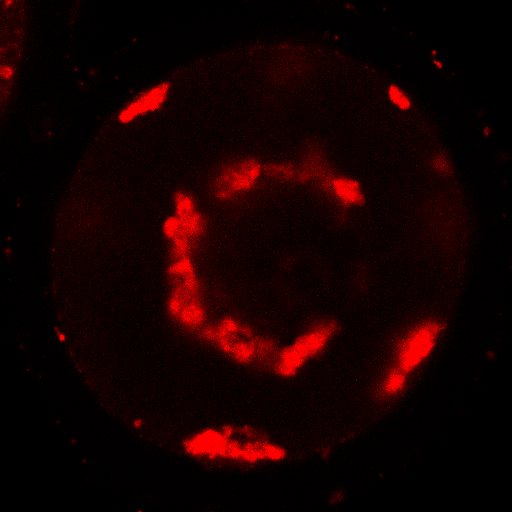

Supplement: Supplementary file 9 — Source data Fig. 5 [file 44319_2025_537_MOESM9_ESM.zip › 5A/Klhl8oo--/Klhl8oo--_TOM20.tif]

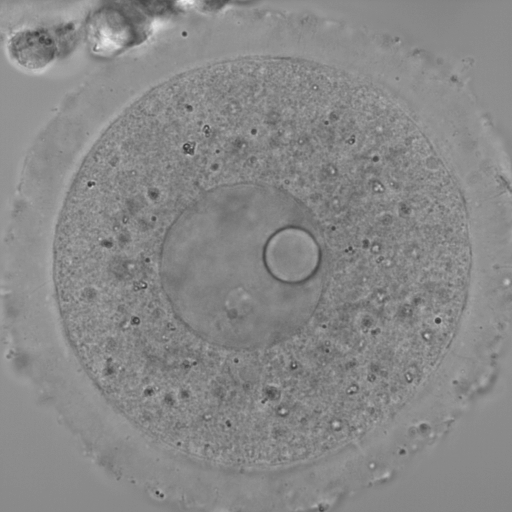

Supplement: Supplementary file 9 — Source data Fig. 5 [file 44319_2025_537_MOESM9_ESM.zip › 5A/WT/WT_bright.tif]

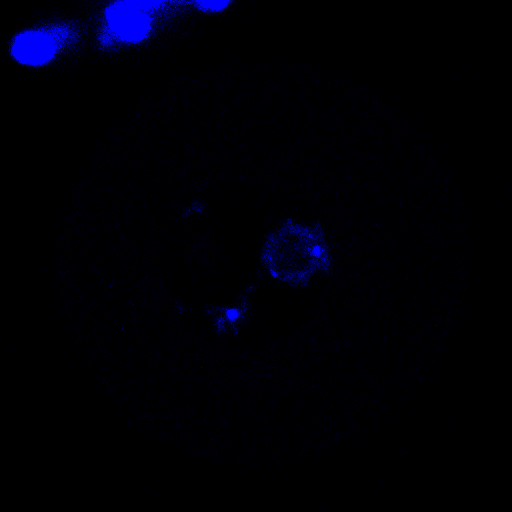

Supplement: Supplementary file 9 — Source data Fig. 5 [file 44319_2025_537_MOESM9_ESM.zip › 5A/WT/WT_hoechst.tif]

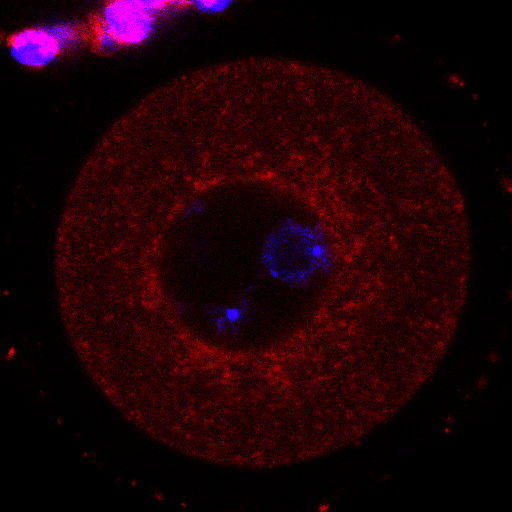

Supplement: Supplementary file 9 — Source data Fig. 5 [file 44319_2025_537_MOESM9_ESM.zip › 5A/WT/WT_merge.tif]

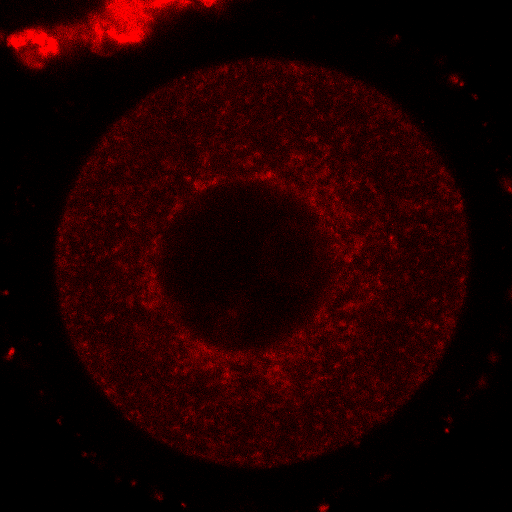

Supplement: Supplementary file 9 — Source data Fig. 5 [file 44319_2025_537_MOESM9_ESM.zip › 5A/WT/WT_TOM20.tif]

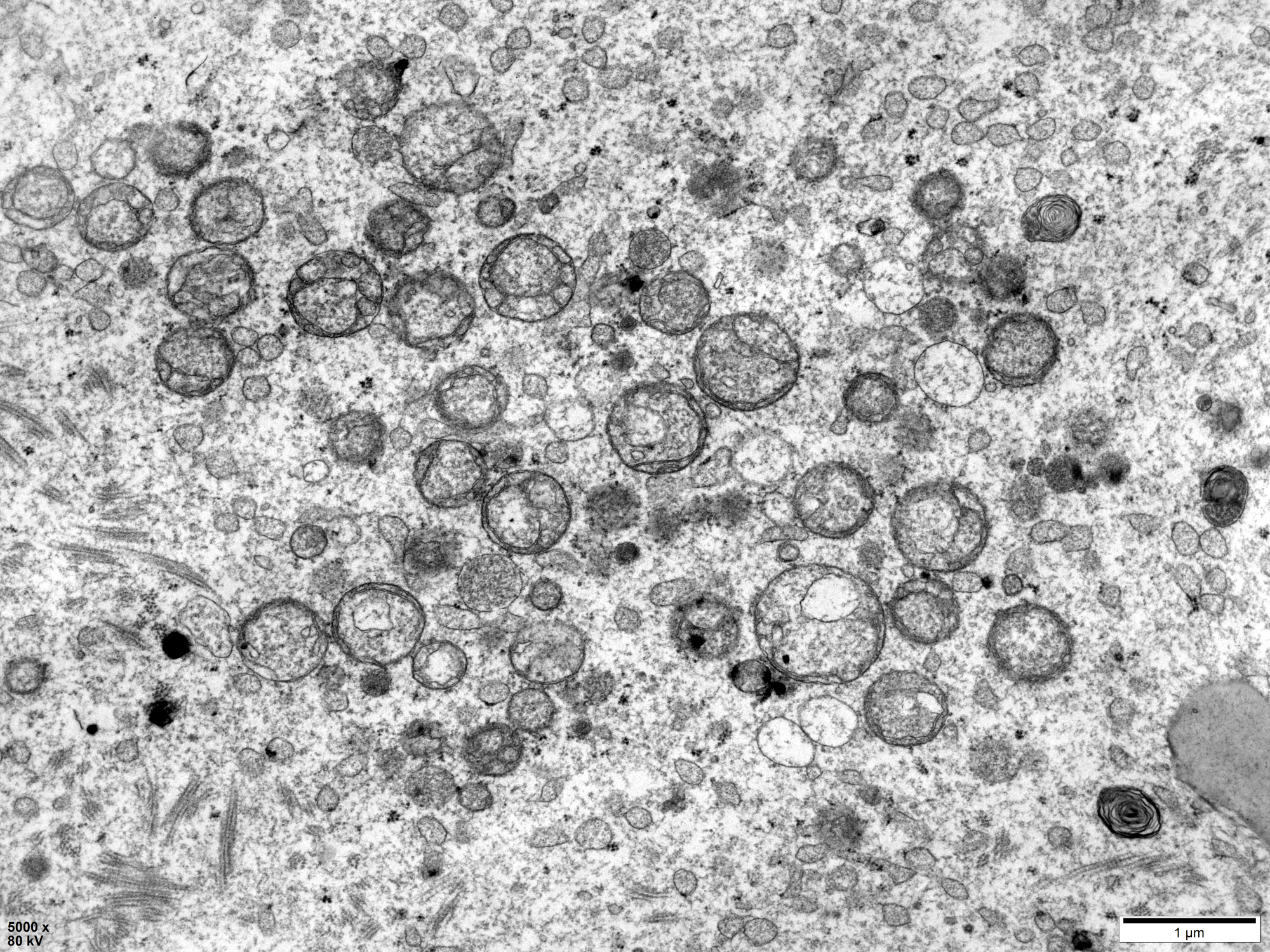

Supplement: Supplementary file 9 — Source data Fig. 5 [file 44319_2025_537_MOESM9_ESM.zip › 5B/Klhl8oo--.tif]

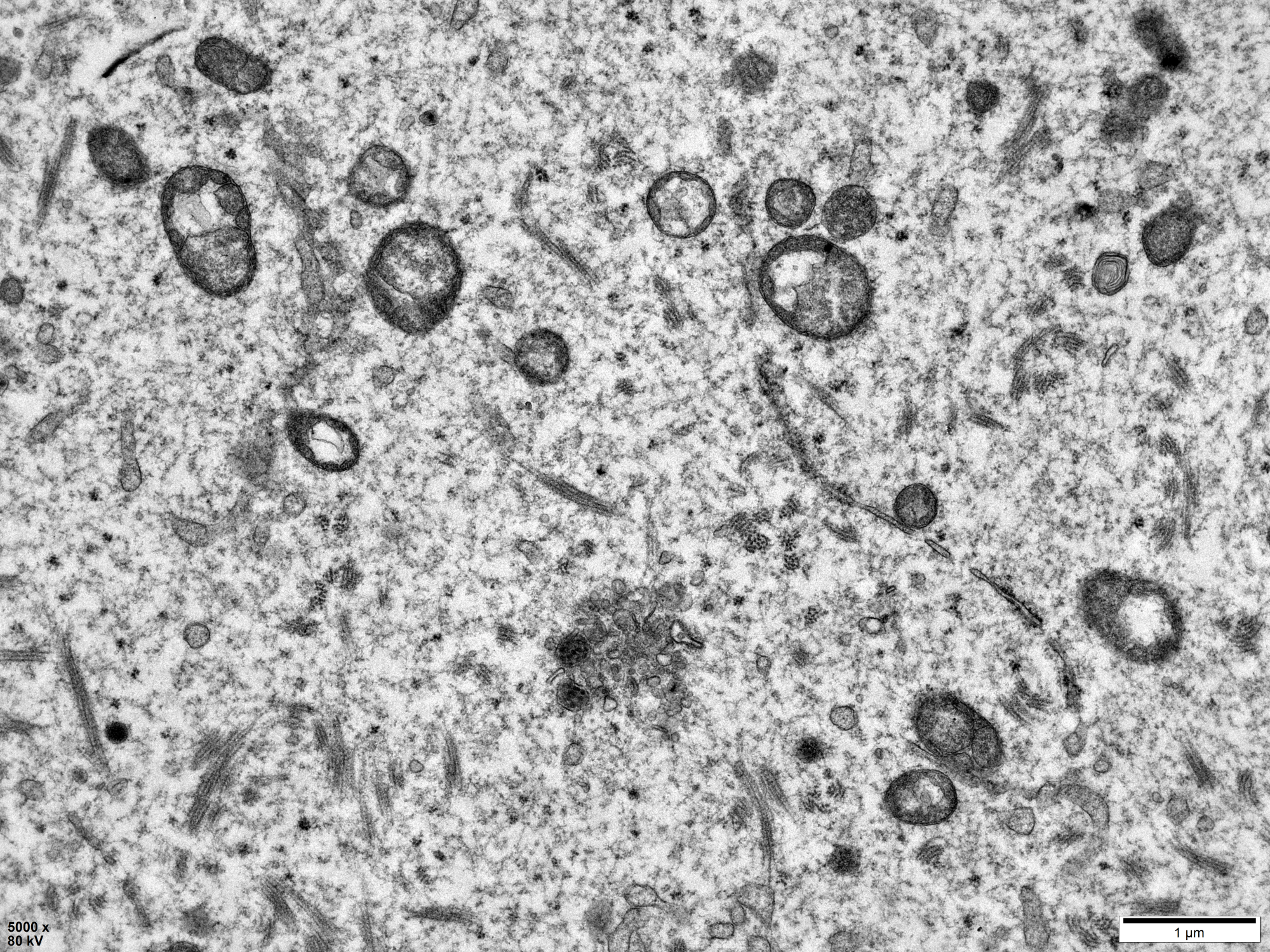

Supplement: Supplementary file 9 — Source data Fig. 5 [file 44319_2025_537_MOESM9_ESM.zip › 5B/WT.tif]

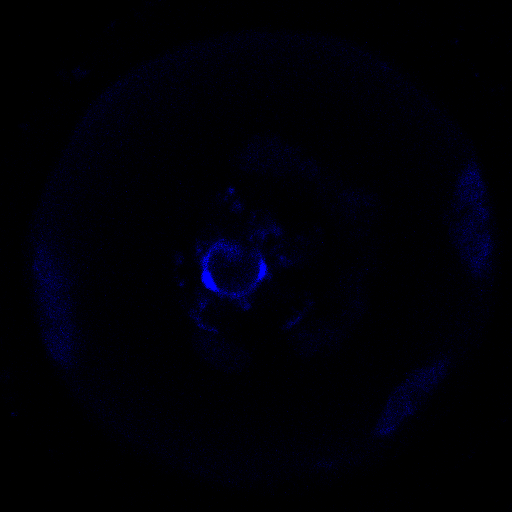

Supplement: Supplementary file 9 — Source data Fig. 5 [file 44319_2025_537_MOESM9_ESM.zip › 5C/Klhl8oo--/Klhl8oo--_DNA.tif]

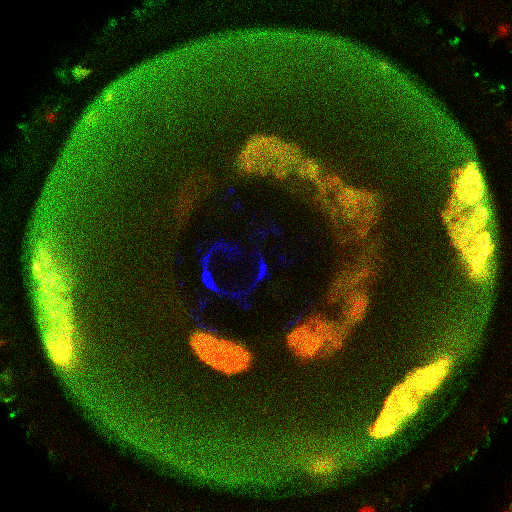

Supplement: Supplementary file 9 — Source data Fig. 5 [file 44319_2025_537_MOESM9_ESM.zip › 5C/Klhl8oo--/Klhl8oo--_merge.tif]

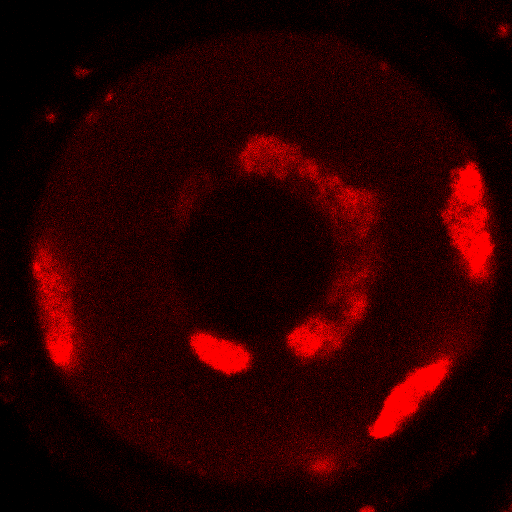

Supplement: Supplementary file 9 — Source data Fig. 5 [file 44319_2025_537_MOESM9_ESM.zip › 5C/Klhl8oo--/Klhl8oo--_MitoTracker.tif]

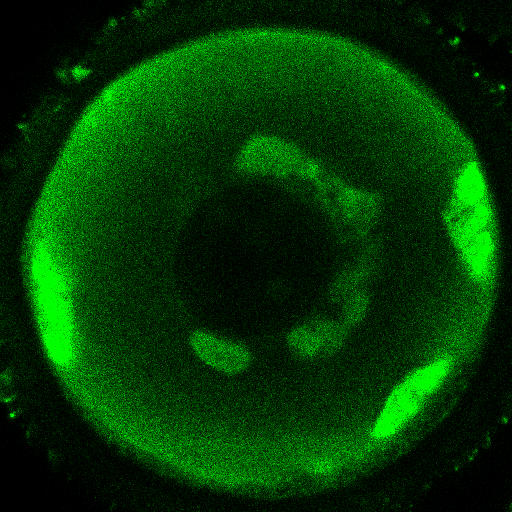

Supplement: Supplementary file 9 — Source data Fig. 5 [file 44319_2025_537_MOESM9_ESM.zip › 5C/Klhl8oo--/Klhl8oo--_ZAR1.tif]

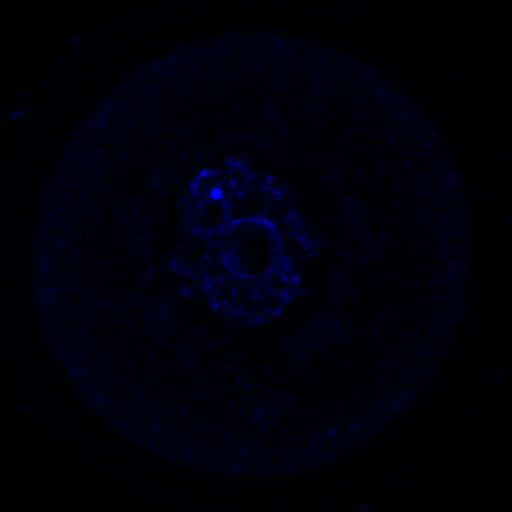

Supplement: Supplementary file 9 — Source data Fig. 5 [file 44319_2025_537_MOESM9_ESM.zip › 5C/WT/WT_DNA.tif]

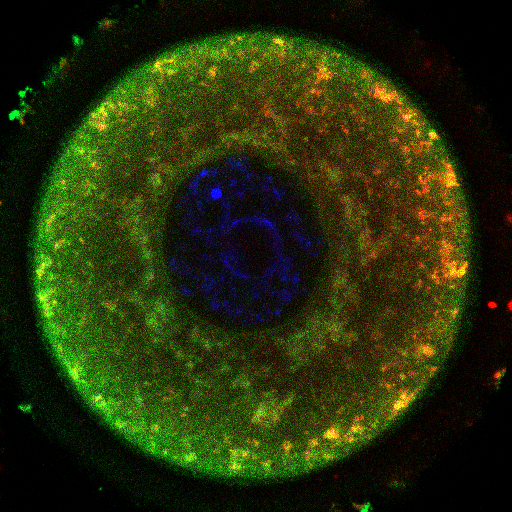

Supplement: Supplementary file 9 — Source data Fig. 5 [file 44319_2025_537_MOESM9_ESM.zip › 5C/WT/WT_merge.tif]

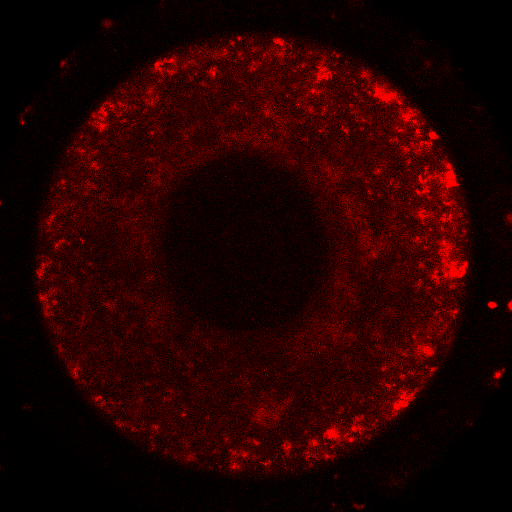

Supplement: Supplementary file 9 — Source data Fig. 5 [file 44319_2025_537_MOESM9_ESM.zip › 5C/WT/WT_MitoTracker.tif]

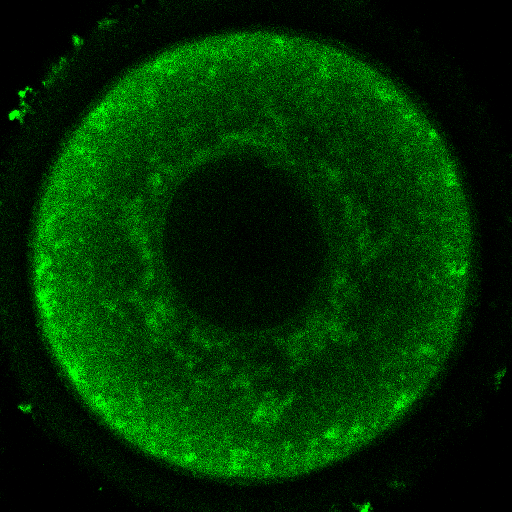

Supplement: Supplementary file 9 — Source data Fig. 5 [file 44319_2025_537_MOESM9_ESM.zip › 5C/WT/WT_ZAR1.tif]

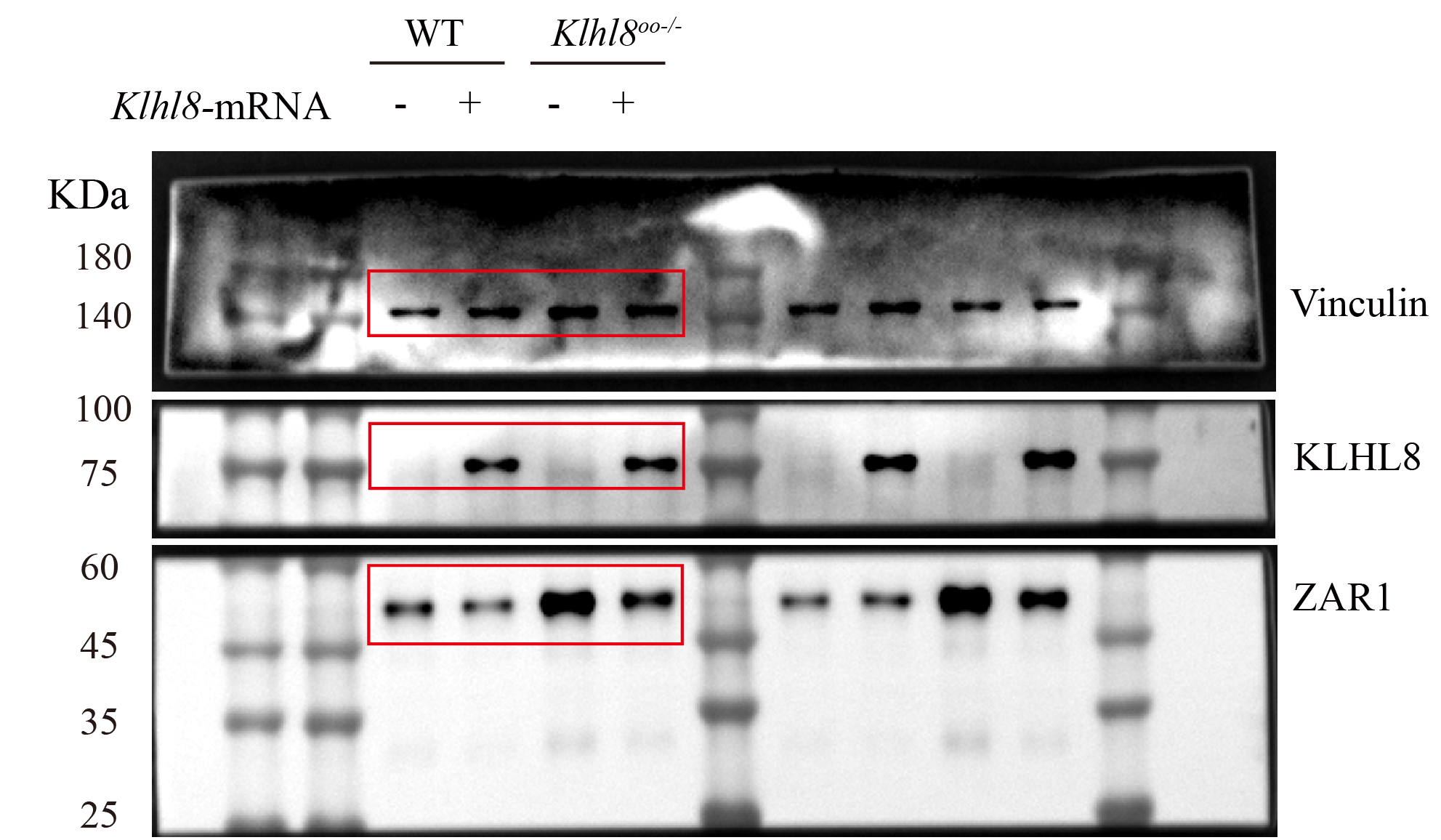

Supplement: Supplementary file 10 — Source data Fig. 6 [file 44319_2025_537_MOESM10_ESM.zip › 6A/6A.tif]

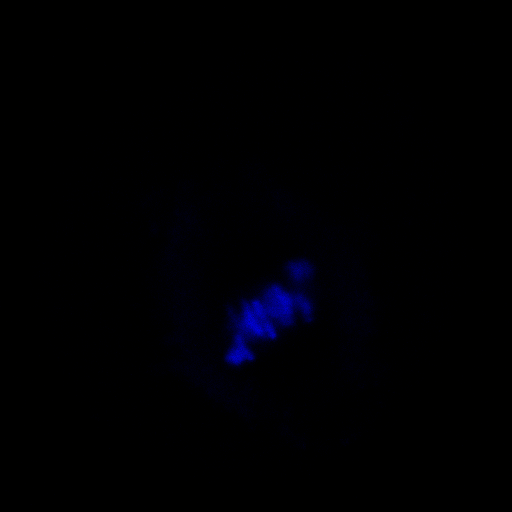

Supplement: Supplementary file 10 — Source data Fig. 6 [file 44319_2025_537_MOESM10_ESM.zip › 6C/Klhl8oo-- _mRNA MI/Klhl8oo-- _mRNA MI_DNA.tif]

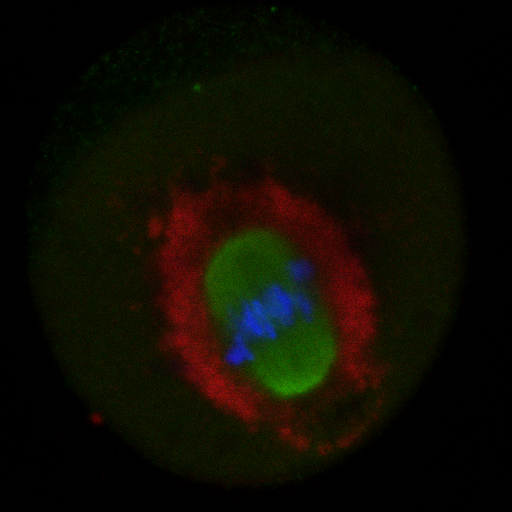

Supplement: Supplementary file 10 — Source data Fig. 6 [file 44319_2025_537_MOESM10_ESM.zip › 6C/Klhl8oo-- _mRNA MI/Klhl8oo-- _mRNA MI_merge.tif]

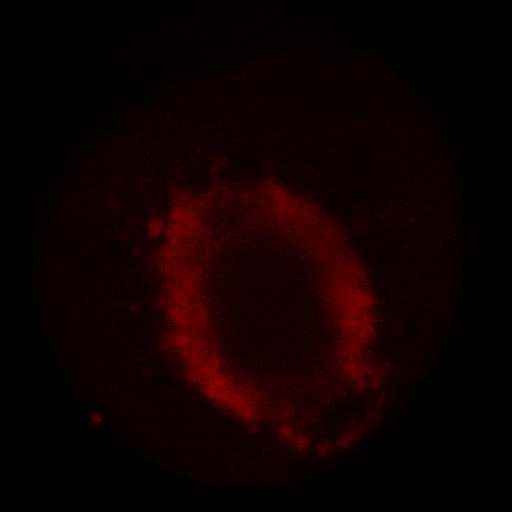

Supplement: Supplementary file 10 — Source data Fig. 6 [file 44319_2025_537_MOESM10_ESM.zip › 6C/Klhl8oo-- _mRNA MI/Klhl8oo-- _mRNA MI_MitoTracker.tif]

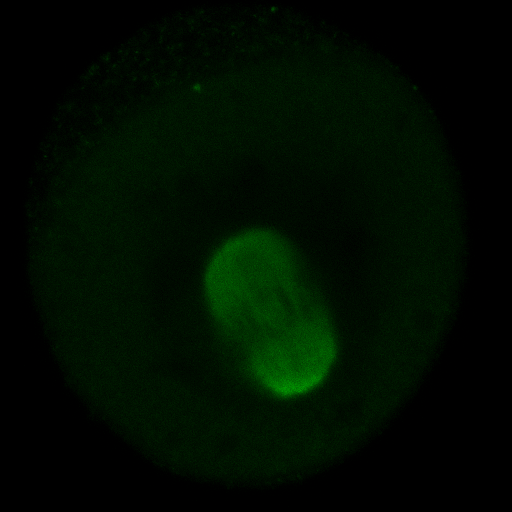

Supplement: Supplementary file 10 — Source data Fig. 6 [file 44319_2025_537_MOESM10_ESM.zip › 6C/Klhl8oo-- _mRNA MI/Klhl8oo-- _mRNA MI_tubulin.tif]

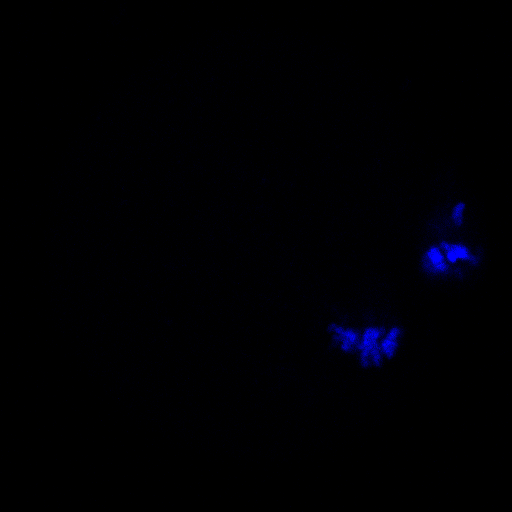

Supplement: Supplementary file 10 — Source data Fig. 6 [file 44319_2025_537_MOESM10_ESM.zip › 6C/Klhl8oo-- _mRNA MII/Klhl8oo-- _mRNA MII_DNA.tif]

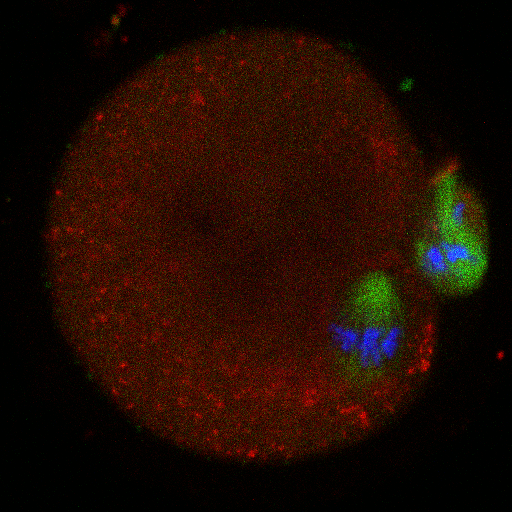

Supplement: Supplementary file 10 — Source data Fig. 6 [file 44319_2025_537_MOESM10_ESM.zip › 6C/Klhl8oo-- _mRNA MII/Klhl8oo-- _mRNA MII_merge.tif]

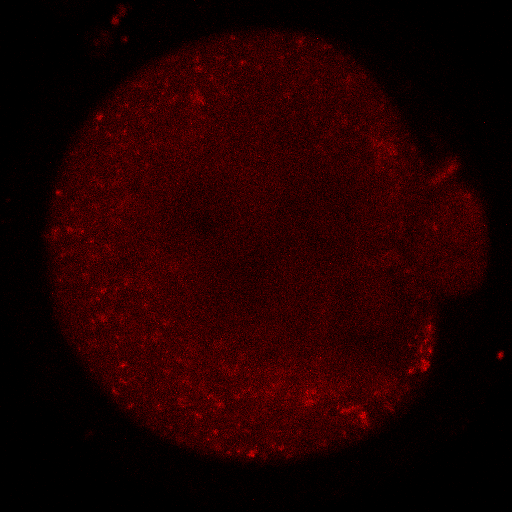

Supplement: Supplementary file 10 — Source data Fig. 6 [file 44319_2025_537_MOESM10_ESM.zip › 6C/Klhl8oo-- _mRNA MII/Klhl8oo-- _mRNA MII_MitoTracker.tif]

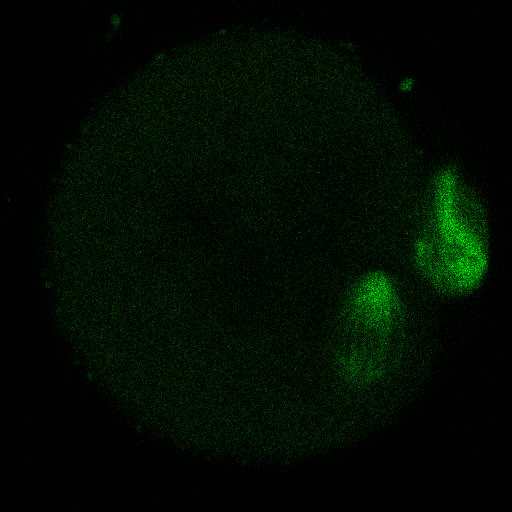

Supplement: Supplementary file 10 — Source data Fig. 6 [file 44319_2025_537_MOESM10_ESM.zip › 6C/Klhl8oo-- _mRNA MII/Klhl8oo-- _mRNA MII_tubulin.tif]

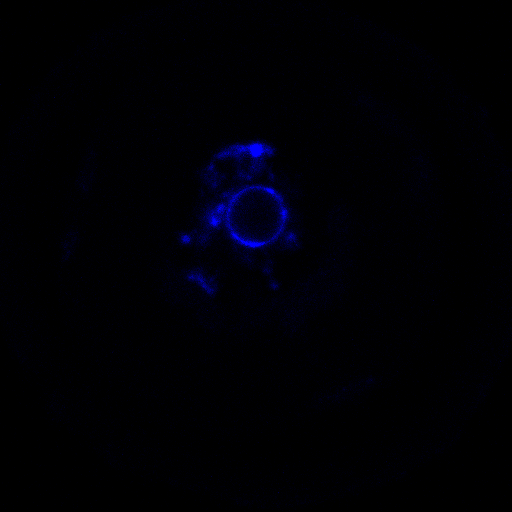

Supplement: Supplementary file 10 — Source data Fig. 6 [file 44319_2025_537_MOESM10_ESM.zip › 6C/Klhl8oo-- GV/Klhl8oo-- GV_DNA.tif]

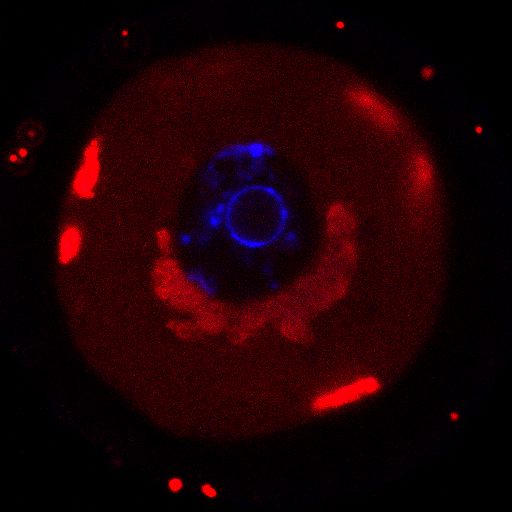

Supplement: Supplementary file 10 — Source data Fig. 6 [file 44319_2025_537_MOESM10_ESM.zip › 6C/Klhl8oo-- GV/Klhl8oo-- GV_merge.tif]

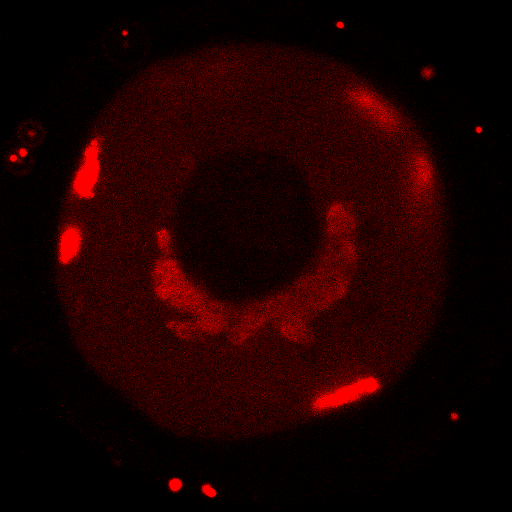

Supplement: Supplementary file 10 — Source data Fig. 6 [file 44319_2025_537_MOESM10_ESM.zip › 6C/Klhl8oo-- GV/Klhl8oo-- GV_MitoTracker.tif]

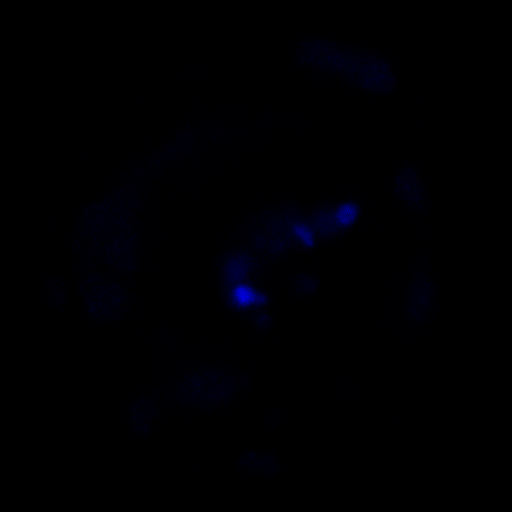

Supplement: Supplementary file 10 — Source data Fig. 6 [file 44319_2025_537_MOESM10_ESM.zip › 6C/Klhl8oo-- MI/Klhl8oo-- MI_DNA.tif]

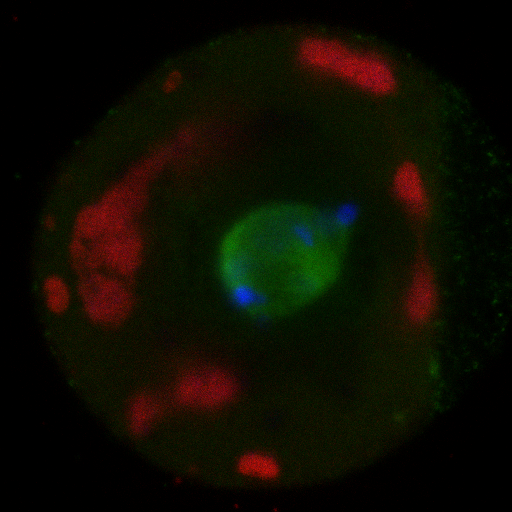

Supplement: Supplementary file 10 — Source data Fig. 6 [file 44319_2025_537_MOESM10_ESM.zip › 6C/Klhl8oo-- MI/Klhl8oo-- MI_merge.tif]

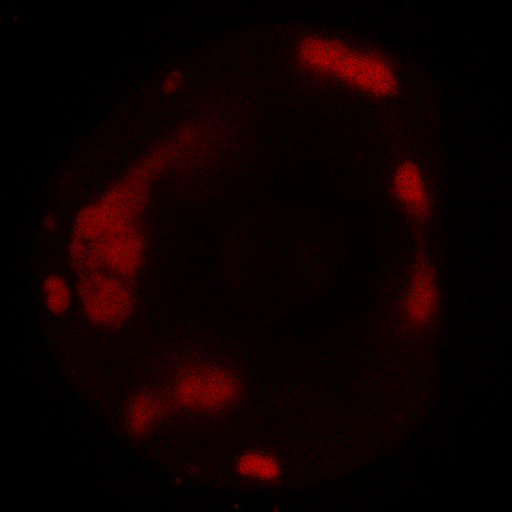

Supplement: Supplementary file 10 — Source data Fig. 6 [file 44319_2025_537_MOESM10_ESM.zip › 6C/Klhl8oo-- MI/Klhl8oo-- MI_MitoTracker.tif]

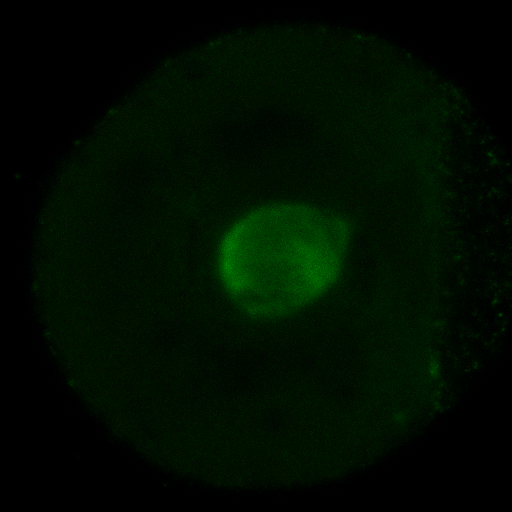

Supplement: Supplementary file 10 — Source data Fig. 6 [file 44319_2025_537_MOESM10_ESM.zip › 6C/Klhl8oo-- MI/Klhl8oo-- MI_tubulin.tif]

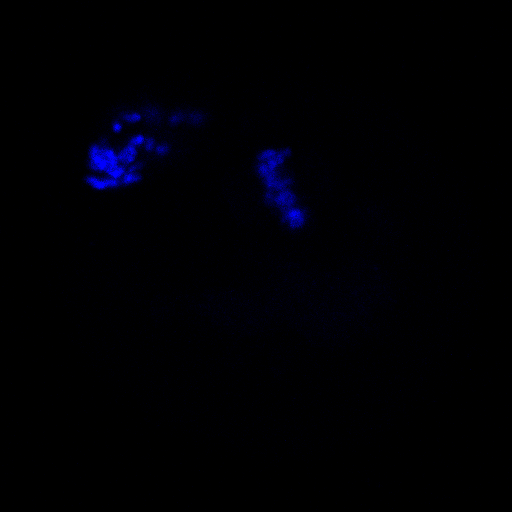

Supplement: Supplementary file 10 — Source data Fig. 6 [file 44319_2025_537_MOESM10_ESM.zip › 6C/Klhl8oo-- MII/Klhl8oo-- MII_DNA.tif]

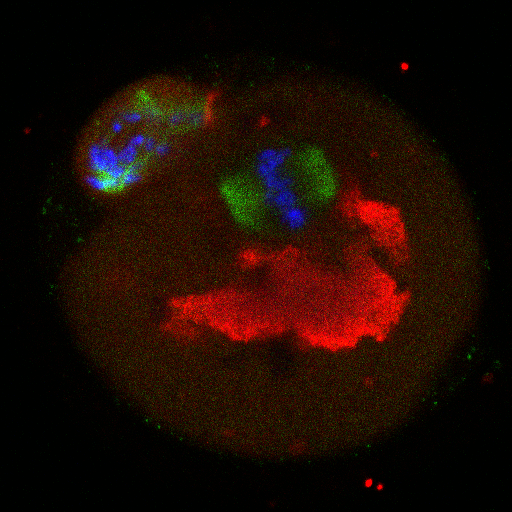

Supplement: Supplementary file 10 — Source data Fig. 6 [file 44319_2025_537_MOESM10_ESM.zip › 6C/Klhl8oo-- MII/Klhl8oo-- MII_merge.tif]
