# Supplementary figures and images for: The E3 ubiquitin ligase adaptor KLHL8 targets ZAR1 to regulate maternal mRNA degradation in oocytes (part 2 of 2)
Source: EMBO Rep. 2025 Jul 28;26(17):4364–87. doi: 10.1038/s44319-025-00537-y (PMC12420792; doi:10.1038/s44319-025-00537-y)

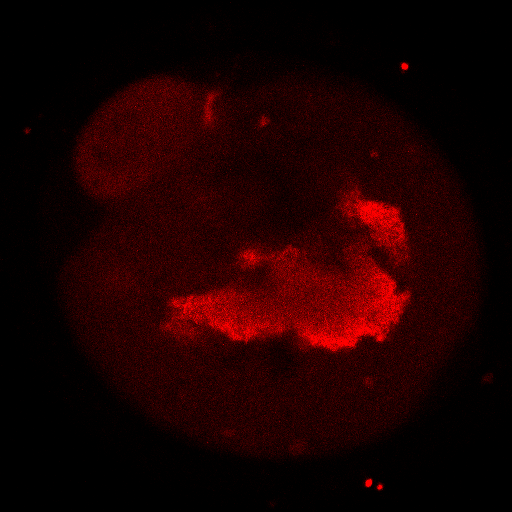

Supplement: Supplementary file 10 — Source data Fig. 6 [file 44319_2025_537_MOESM10_ESM.zip › 6C/Klhl8oo-- MII/Klhl8oo-- MII_MitoTracker.tif]

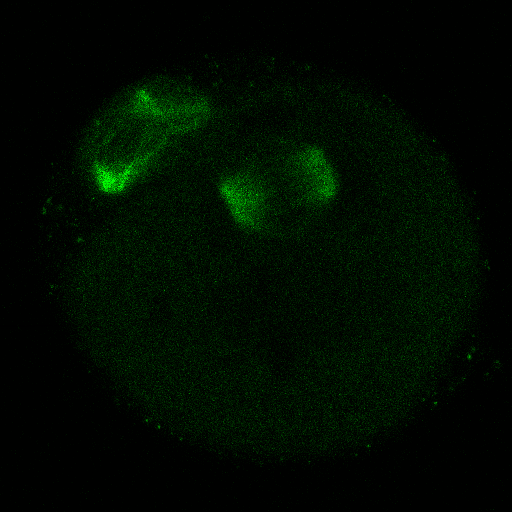

Supplement: Supplementary file 10 — Source data Fig. 6 [file 44319_2025_537_MOESM10_ESM.zip › 6C/Klhl8oo-- MII/Klhl8oo-- MII_tubulin.tif]

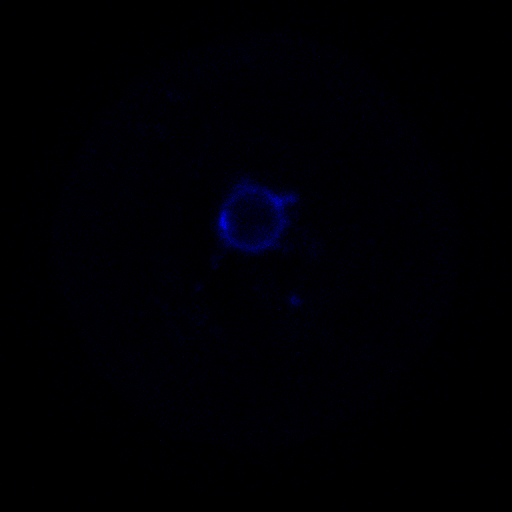

Supplement: Supplementary file 10 — Source data Fig. 6 [file 44319_2025_537_MOESM10_ESM.zip › 6C/Klhl8oo--_mRNA GV/Klhl8oo--_mRNA GV_DNA.tif]

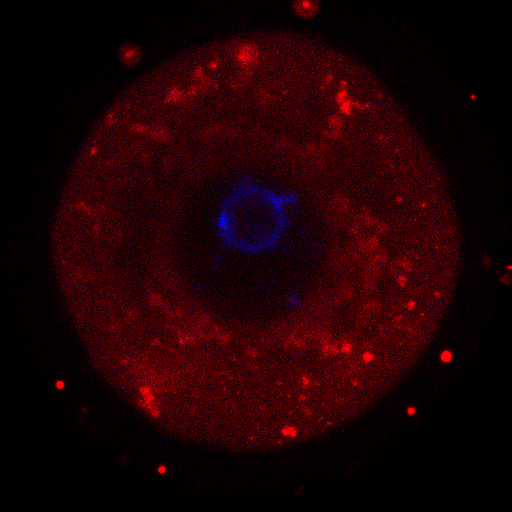

Supplement: Supplementary file 10 — Source data Fig. 6 [file 44319_2025_537_MOESM10_ESM.zip › 6C/Klhl8oo--_mRNA GV/Klhl8oo--_mRNA GV_merge.tif]

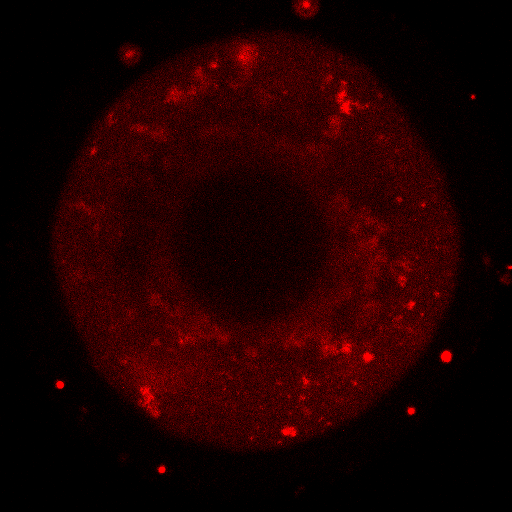

Supplement: Supplementary file 10 — Source data Fig. 6 [file 44319_2025_537_MOESM10_ESM.zip › 6C/Klhl8oo--_mRNA GV/Klhl8oo--_mRNA GV_MitoTracker.tif]

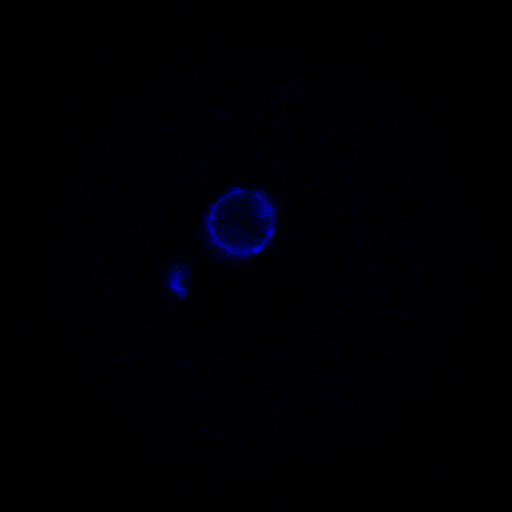

Supplement: Supplementary file 10 — Source data Fig. 6 [file 44319_2025_537_MOESM10_ESM.zip › 6C/WT GV/WT GV_DNA.tif]

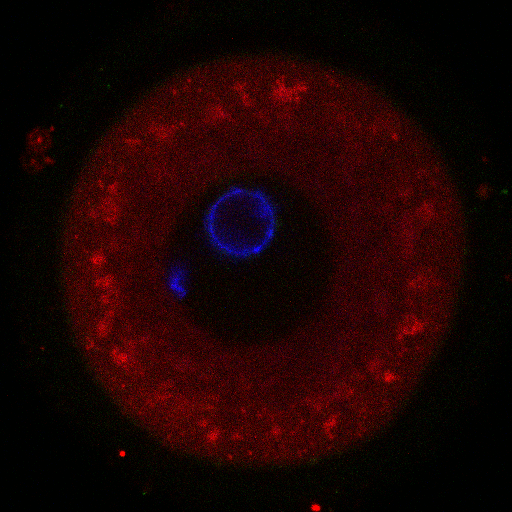

Supplement: Supplementary file 10 — Source data Fig. 6 [file 44319_2025_537_MOESM10_ESM.zip › 6C/WT GV/WT GV_merge.tif]

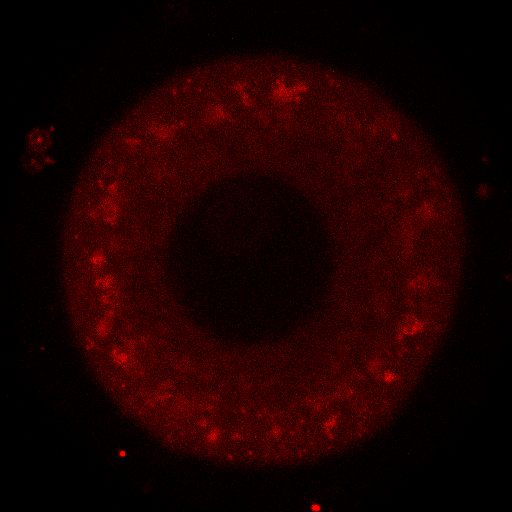

Supplement: Supplementary file 10 — Source data Fig. 6 [file 44319_2025_537_MOESM10_ESM.zip › 6C/WT GV/WT GV_MitoTracker.tif]

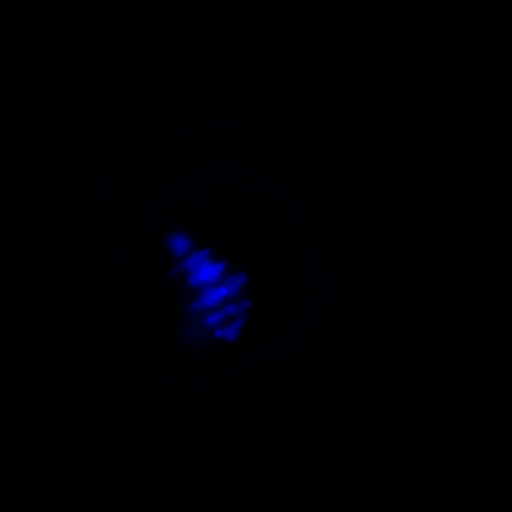

Supplement: Supplementary file 10 — Source data Fig. 6 [file 44319_2025_537_MOESM10_ESM.zip › 6C/WT MI/WT MI_DNA.tif]

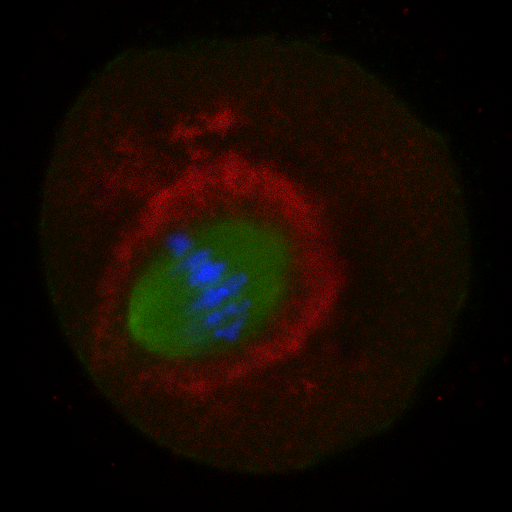

Supplement: Supplementary file 10 — Source data Fig. 6 [file 44319_2025_537_MOESM10_ESM.zip › 6C/WT MI/WT MI_merge.tif]

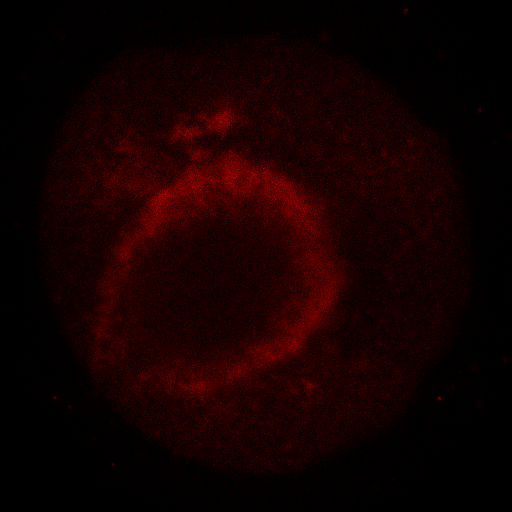

Supplement: Supplementary file 10 — Source data Fig. 6 [file 44319_2025_537_MOESM10_ESM.zip › 6C/WT MI/WT MI_MitoTracker.tif]

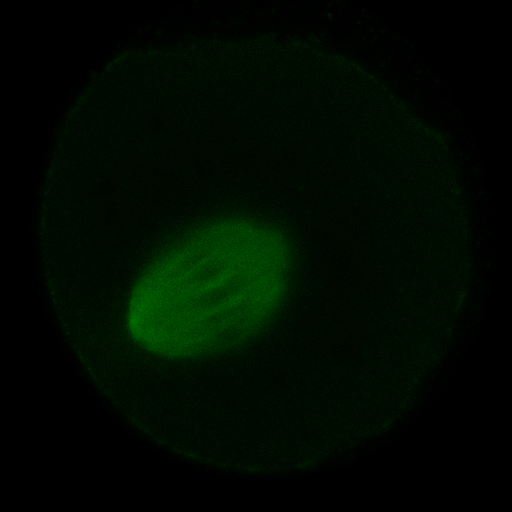

Supplement: Supplementary file 10 — Source data Fig. 6 [file 44319_2025_537_MOESM10_ESM.zip › 6C/WT MI/WT MI_tubulin.tif]

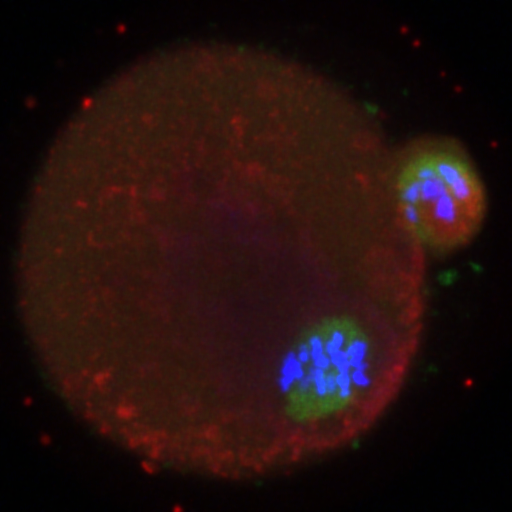

Supplement: Supplementary file 10 — Source data Fig. 6 [file 44319_2025_537_MOESM10_ESM.zip › 6C/WT MII/WT _MII_c1+2+3.tif]

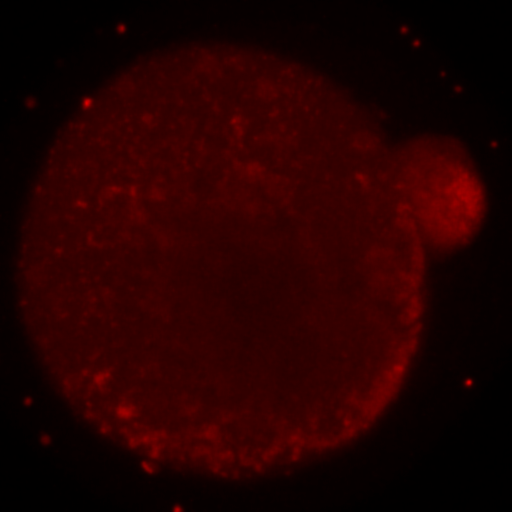

Supplement: Supplementary file 10 — Source data Fig. 6 [file 44319_2025_537_MOESM10_ESM.zip › 6C/WT MII/WT _MII_c1.tif]

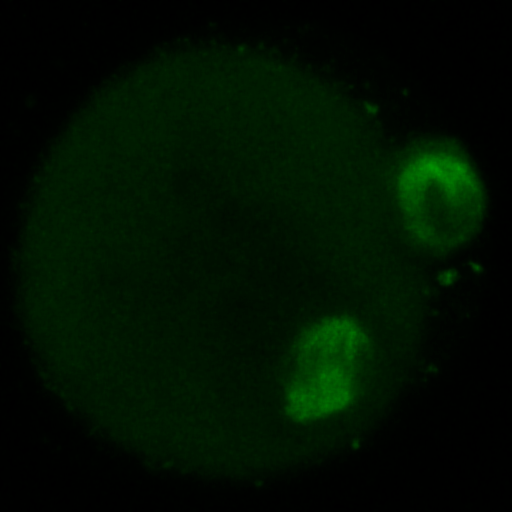

Supplement: Supplementary file 10 — Source data Fig. 6 [file 44319_2025_537_MOESM10_ESM.zip › 6C/WT MII/WT _MII_c2.tif]

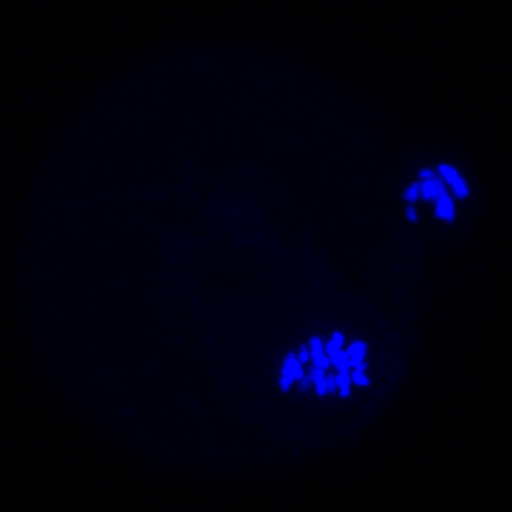

Supplement: Supplementary file 10 — Source data Fig. 6 [file 44319_2025_537_MOESM10_ESM.zip › 6C/WT MII/WT _MII_c3.tif]

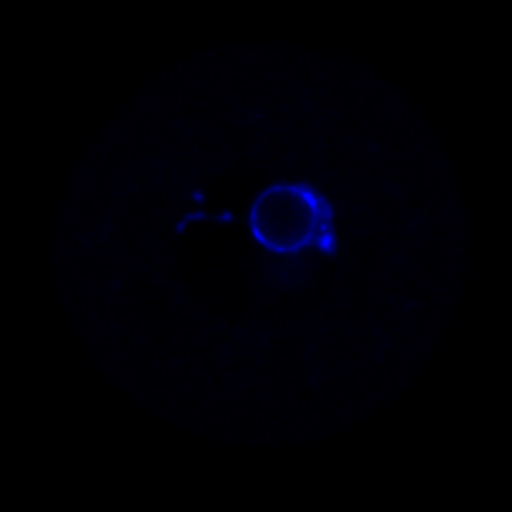

Supplement: Supplementary file 10 — Source data Fig. 6 [file 44319_2025_537_MOESM10_ESM.zip › 6C/WT mRNA GV/WT mRNA GV_DNA.tif]

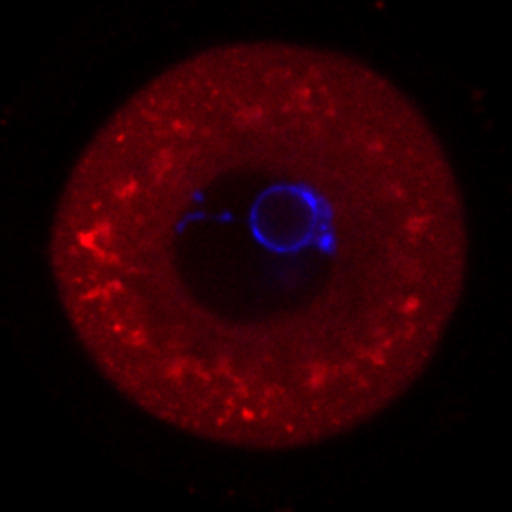

Supplement: Supplementary file 10 — Source data Fig. 6 [file 44319_2025_537_MOESM10_ESM.zip › 6C/WT mRNA GV/WT mRNA GV_merge.tif]

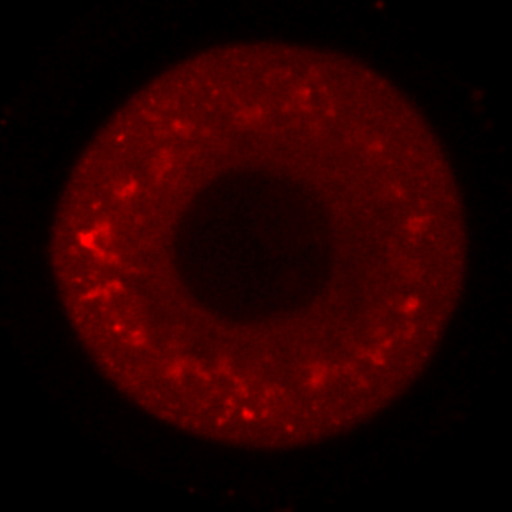

Supplement: Supplementary file 10 — Source data Fig. 6 [file 44319_2025_537_MOESM10_ESM.zip › 6C/WT mRNA GV/WT mRNA GV_MitoTracker.tif]

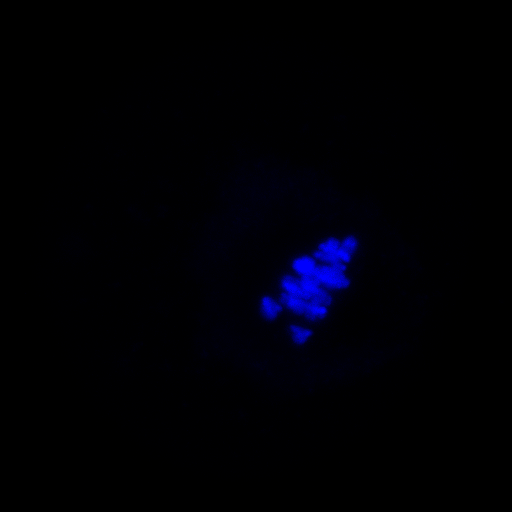

Supplement: Supplementary file 10 — Source data Fig. 6 [file 44319_2025_537_MOESM10_ESM.zip › 6C/WT mRNA MI/WT mRNA MI_DNA.tif]

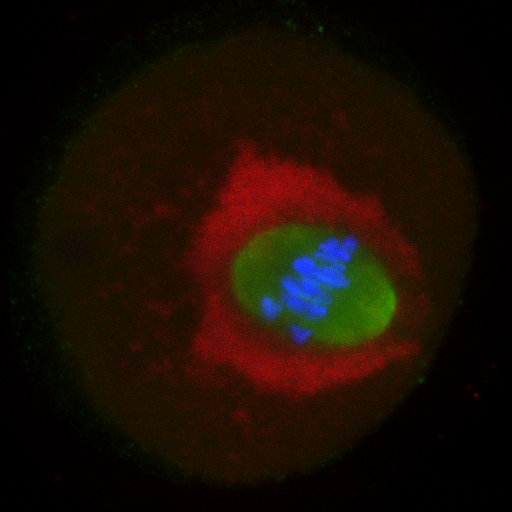

Supplement: Supplementary file 10 — Source data Fig. 6 [file 44319_2025_537_MOESM10_ESM.zip › 6C/WT mRNA MI/WT mRNA MI_merge.tif]

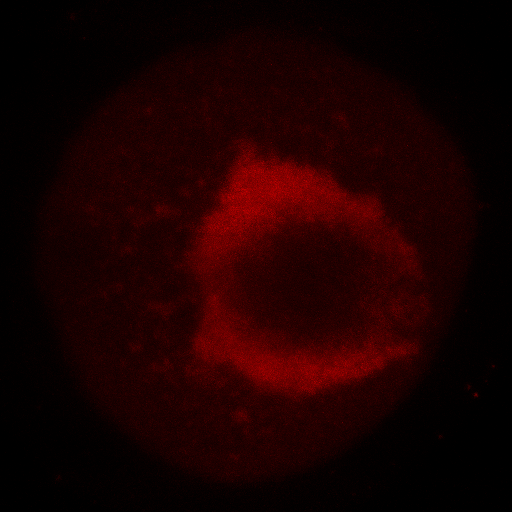

Supplement: Supplementary file 10 — Source data Fig. 6 [file 44319_2025_537_MOESM10_ESM.zip › 6C/WT mRNA MI/WT mRNA MI_MitoTracker.tif]

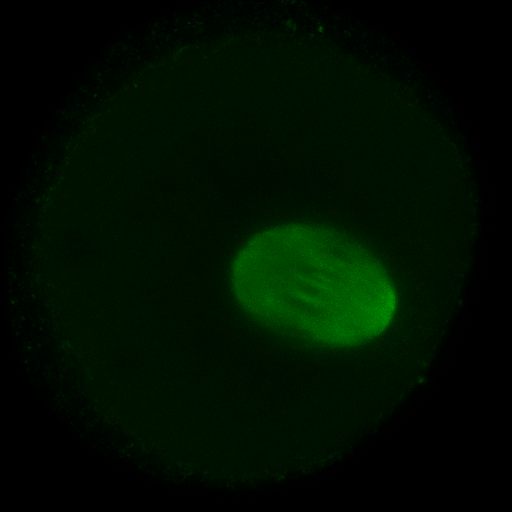

Supplement: Supplementary file 10 — Source data Fig. 6 [file 44319_2025_537_MOESM10_ESM.zip › 6C/WT mRNA MI/WT mRNA MI_tubulin.tif]

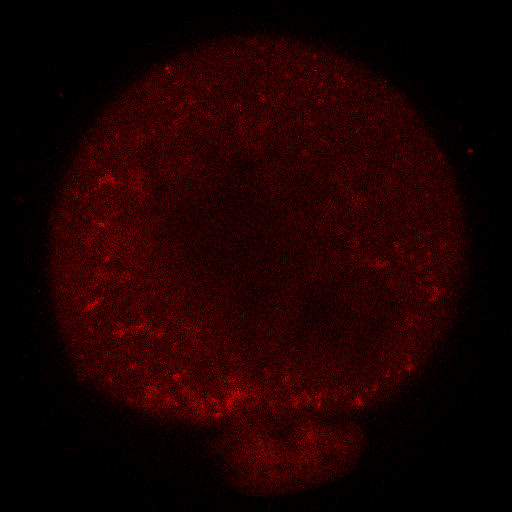

Supplement: Supplementary file 10 — Source data Fig. 6 [file 44319_2025_537_MOESM10_ESM.zip › 6C/WT mRNA MII/WT_mRNA MII_MitoTracker.tif]

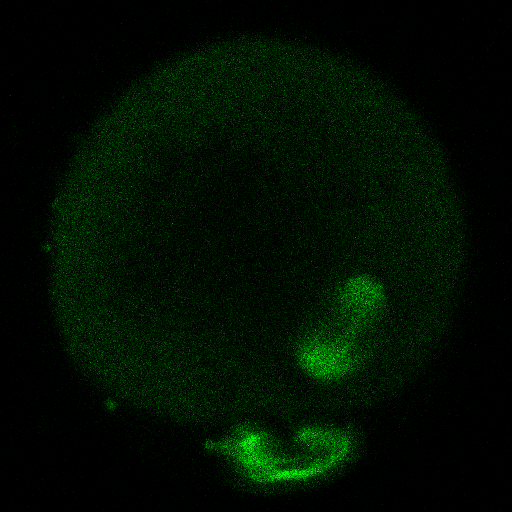

Supplement: Supplementary file 10 — Source data Fig. 6 [file 44319_2025_537_MOESM10_ESM.zip › 6C/WT mRNA MII/WT_mRNA MII_tubulin.tif]

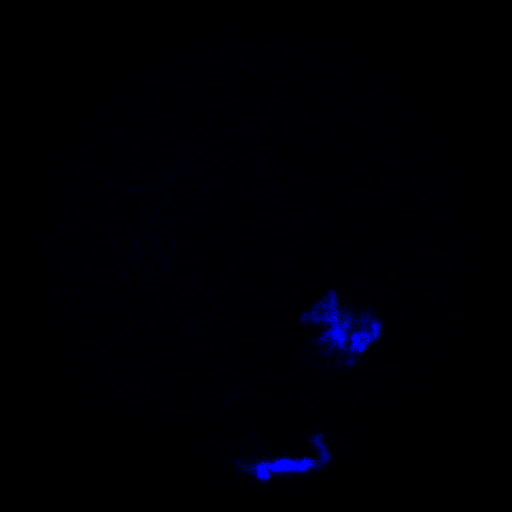

Supplement: Supplementary file 10 — Source data Fig. 6 [file 44319_2025_537_MOESM10_ESM.zip › 6C/WT mRNA MII/WT_mRNA MII_DNA.tif]

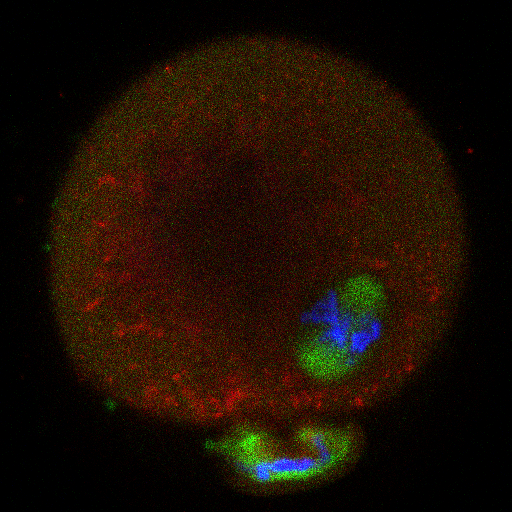

Supplement: Supplementary file 10 — Source data Fig. 6 [file 44319_2025_537_MOESM10_ESM.zip › 6C/WT mRNA MII/WT_mRNA MII_merge.tif]

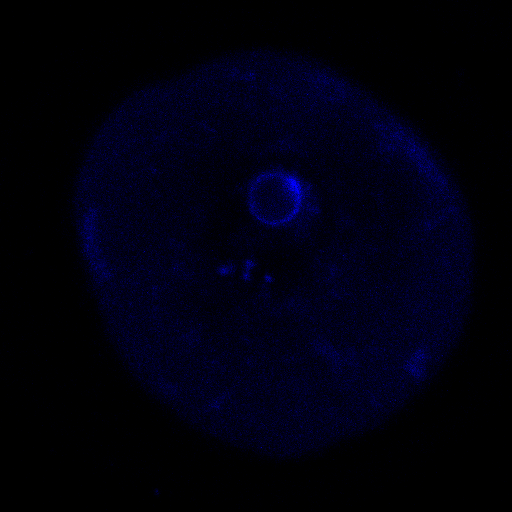

Supplement: Supplementary file 10 — Source data Fig. 6 [file 44319_2025_537_MOESM10_ESM.zip › 6D/Klhl8oo-- _mRNA/Klhl8oo-- _mRNA_DNA.tif]

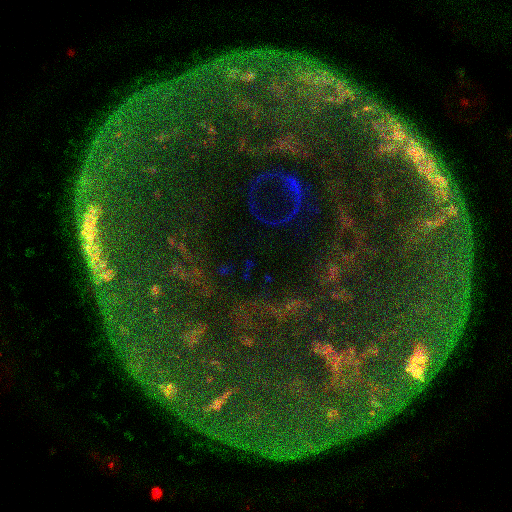

Supplement: Supplementary file 10 — Source data Fig. 6 [file 44319_2025_537_MOESM10_ESM.zip › 6D/Klhl8oo-- _mRNA/Klhl8oo-- _mRNA_merge.tif]

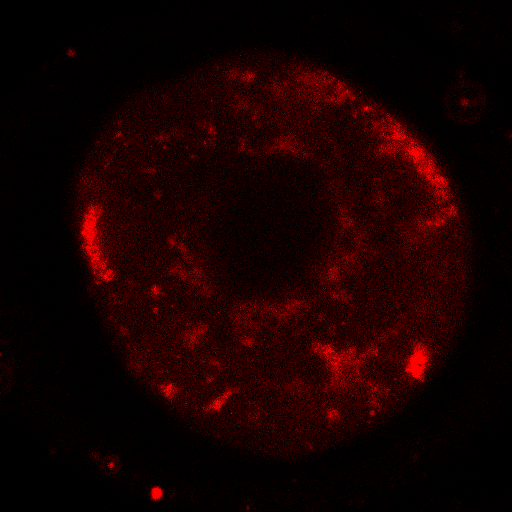

Supplement: Supplementary file 10 — Source data Fig. 6 [file 44319_2025_537_MOESM10_ESM.zip › 6D/Klhl8oo-- _mRNA/Klhl8oo-- _mRNA_MitoTracker.tif]

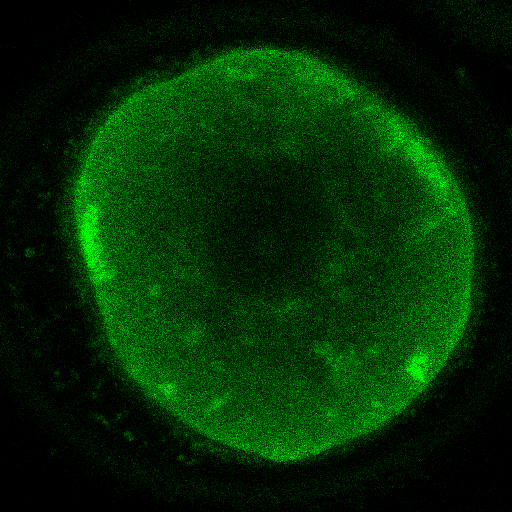

Supplement: Supplementary file 10 — Source data Fig. 6 [file 44319_2025_537_MOESM10_ESM.zip › 6D/Klhl8oo-- _mRNA/Klhl8oo-- _mRNA_ZAR1.tif]

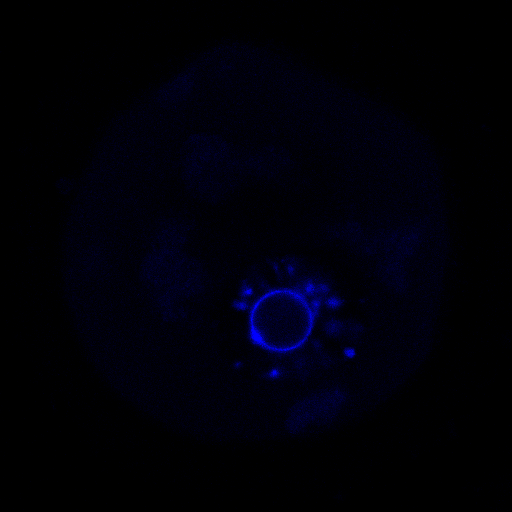

Supplement: Supplementary file 10 — Source data Fig. 6 [file 44319_2025_537_MOESM10_ESM.zip › 6D/Klhl8oo--/Klhl8oo--_DNA.tif]

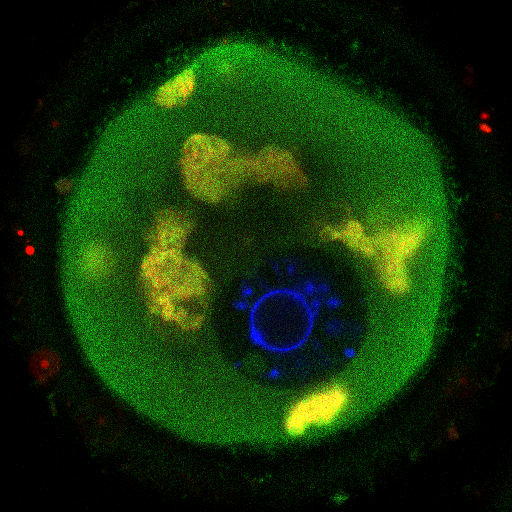

Supplement: Supplementary file 10 — Source data Fig. 6 [file 44319_2025_537_MOESM10_ESM.zip › 6D/Klhl8oo--/Klhl8oo--_merge.tif]

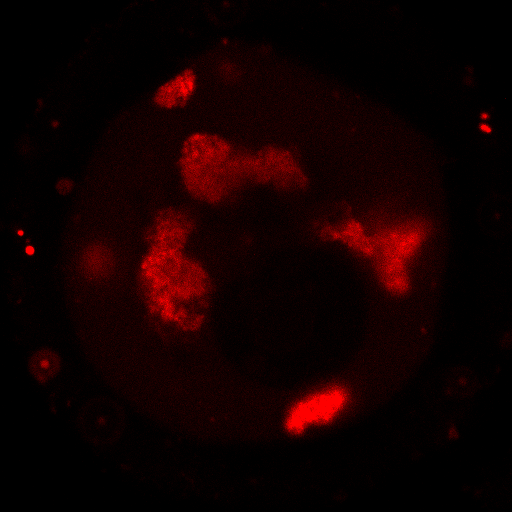

Supplement: Supplementary file 10 — Source data Fig. 6 [file 44319_2025_537_MOESM10_ESM.zip › 6D/Klhl8oo--/Klhl8oo--_MitoTracker.tif]

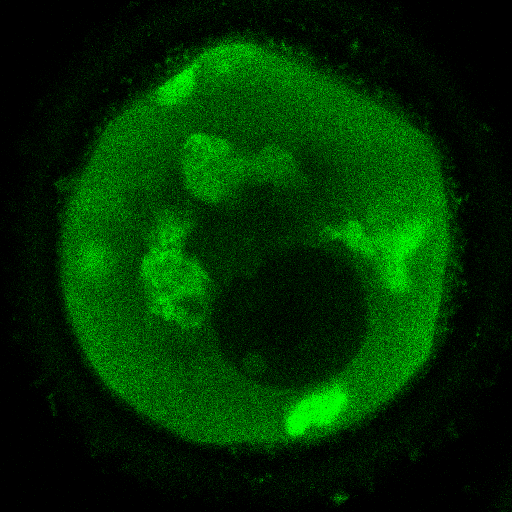

Supplement: Supplementary file 10 — Source data Fig. 6 [file 44319_2025_537_MOESM10_ESM.zip › 6D/Klhl8oo--/Klhl8oo--_ZAR1.tif]

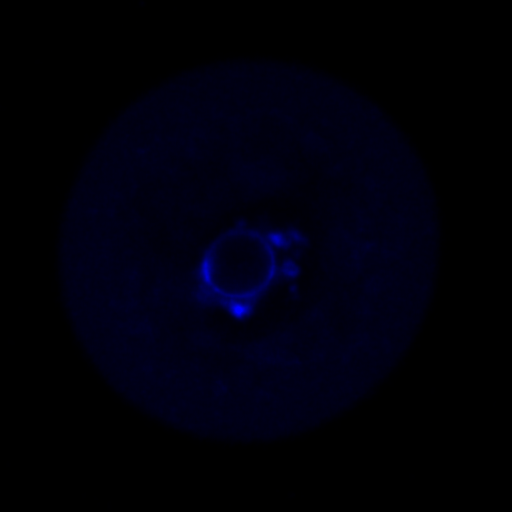

Supplement: Supplementary file 10 — Source data Fig. 6 [file 44319_2025_537_MOESM10_ESM.zip › 6D/WT/WT_DNA.tif]

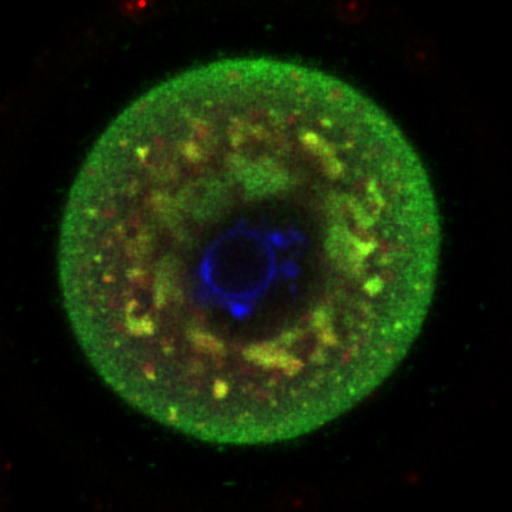

Supplement: Supplementary file 10 — Source data Fig. 6 [file 44319_2025_537_MOESM10_ESM.zip › 6D/WT/WT_merge.tif]

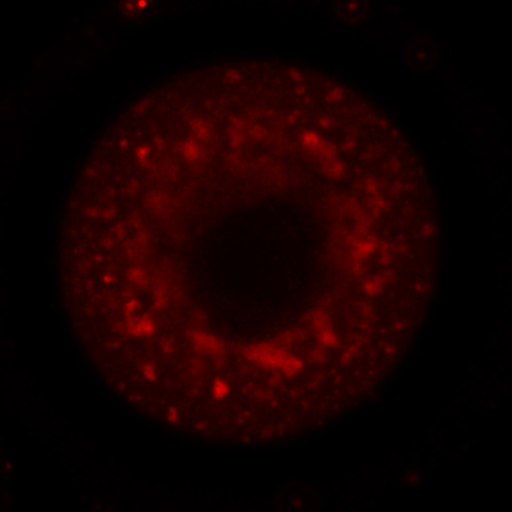

Supplement: Supplementary file 10 — Source data Fig. 6 [file 44319_2025_537_MOESM10_ESM.zip › 6D/WT/WT_MitoTracker.tif]

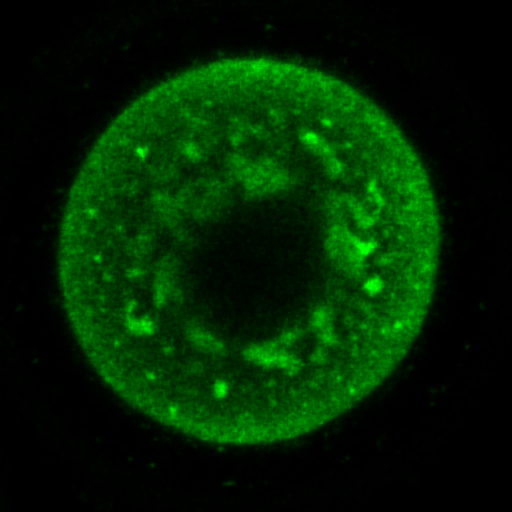

Supplement: Supplementary file 10 — Source data Fig. 6 [file 44319_2025_537_MOESM10_ESM.zip › 6D/WT/WT_ZAR1.tif]

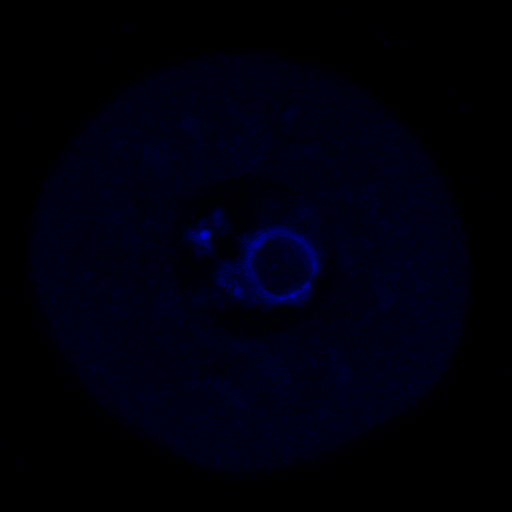

Supplement: Supplementary file 10 — Source data Fig. 6 [file 44319_2025_537_MOESM10_ESM.zip › 6D/WT_mRNA/WT_mRNA_DNA.tif]

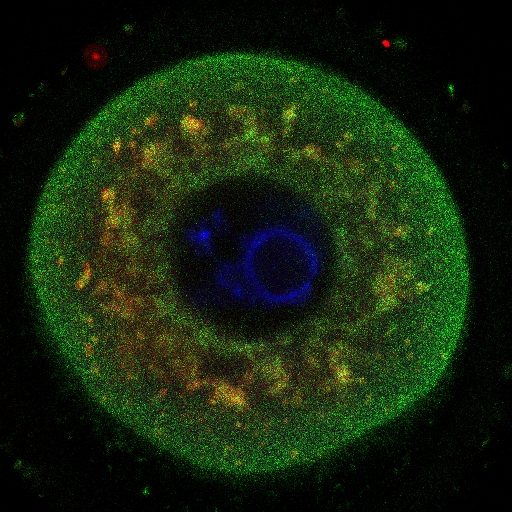

Supplement: Supplementary file 10 — Source data Fig. 6 [file 44319_2025_537_MOESM10_ESM.zip › 6D/WT_mRNA/WT_mRNA_merge.tif]

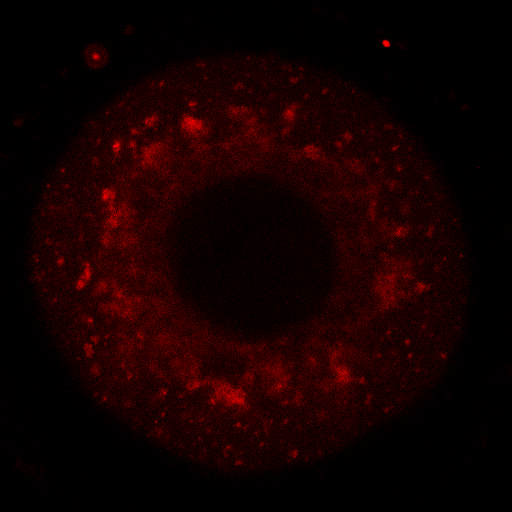

Supplement: Supplementary file 10 — Source data Fig. 6 [file 44319_2025_537_MOESM10_ESM.zip › 6D/WT_mRNA/WT_mRNA_MitoTracker.tif]

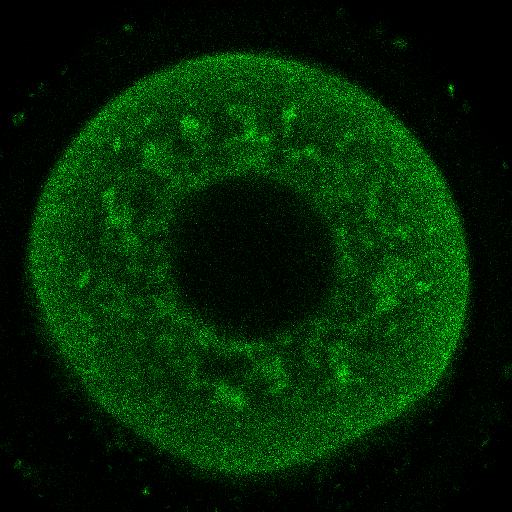

Supplement: Supplementary file 10 — Source data Fig. 6 [file 44319_2025_537_MOESM10_ESM.zip › 6D/WT_mRNA/WT_mRNA_ZAR1.tif]

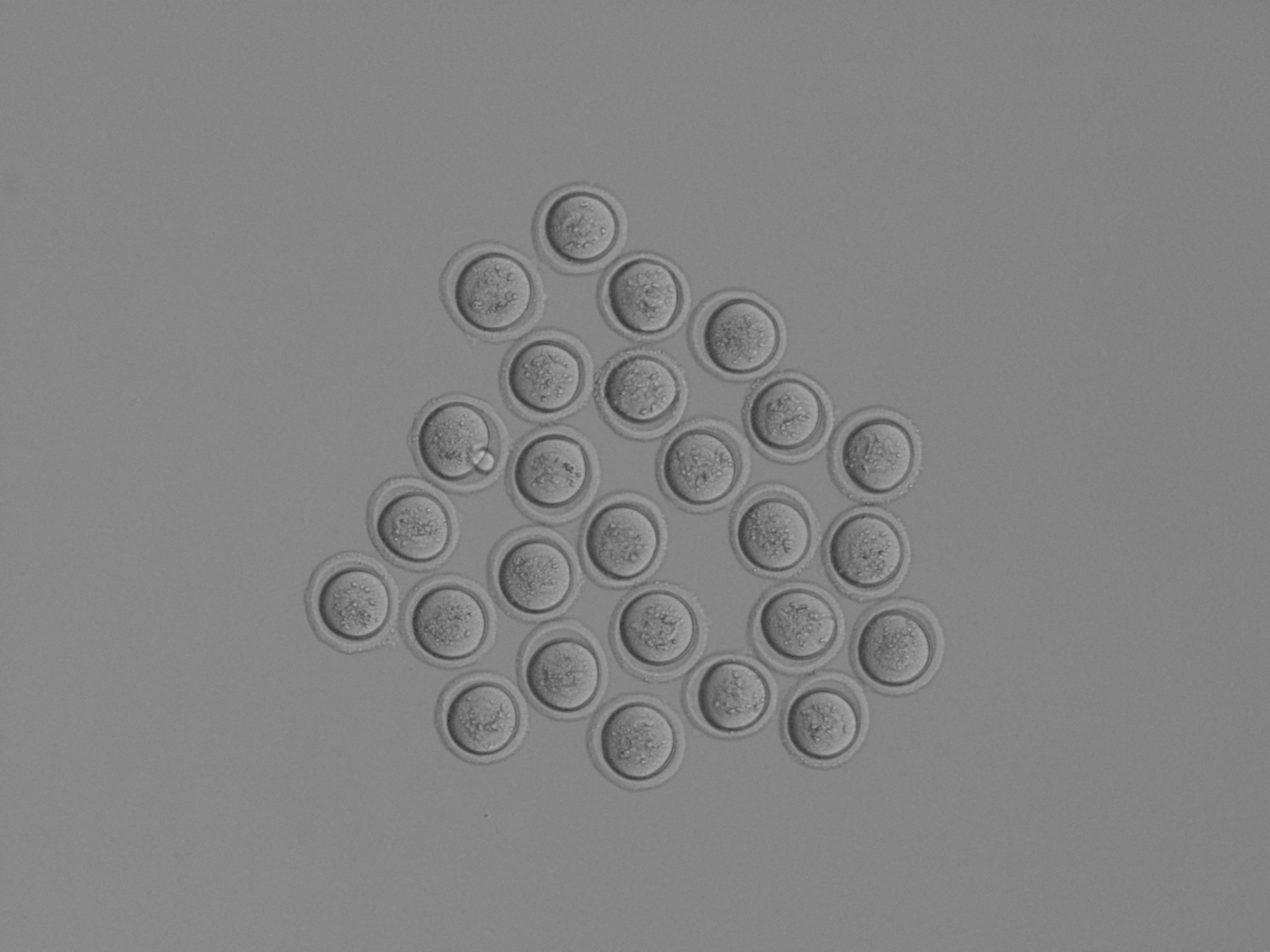

Supplement: Supplementary file 10 — Source data Fig. 6 [file 44319_2025_537_MOESM10_ESM.zip › 6G/Klhl8oo--.tif]

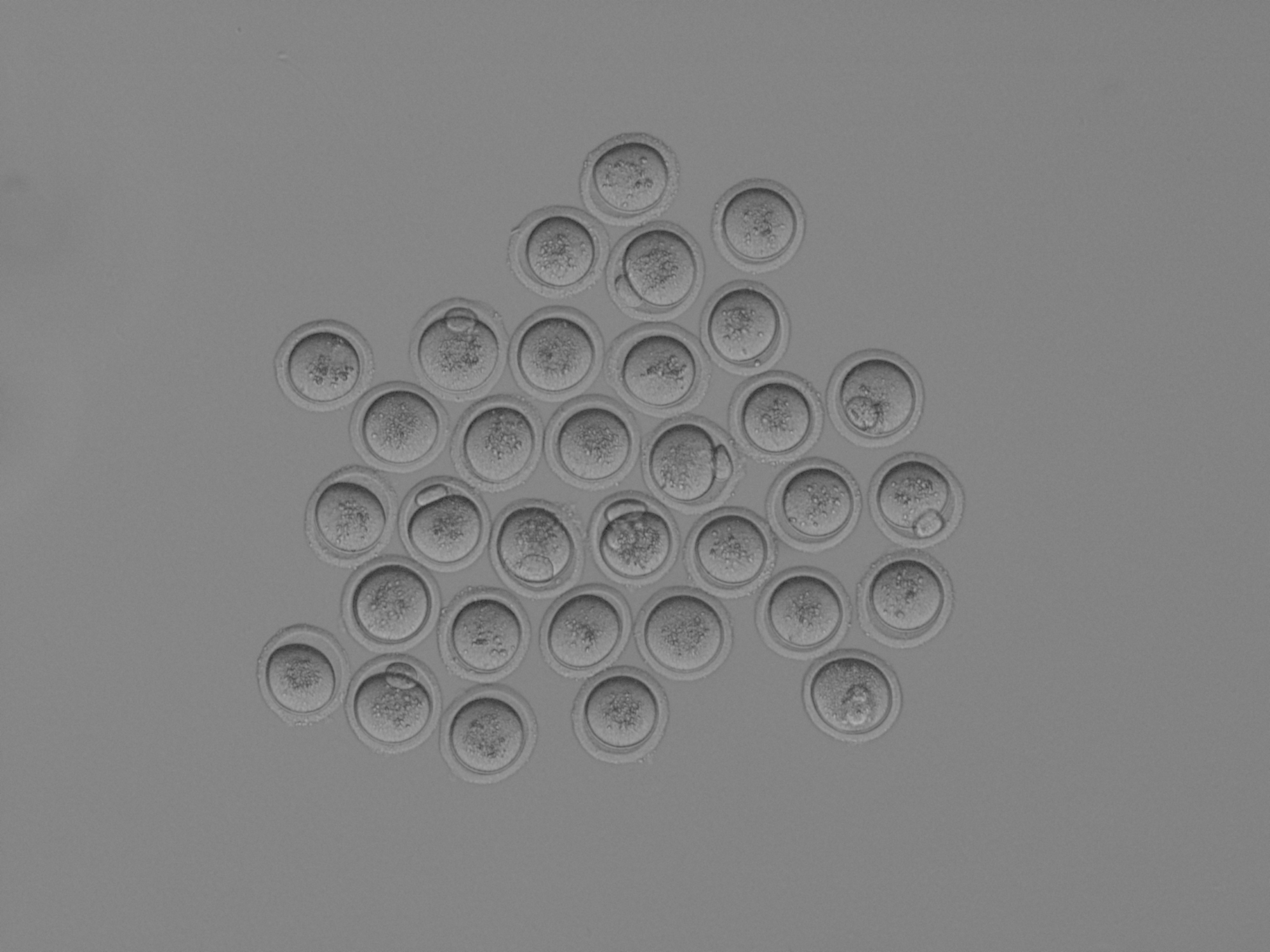

Supplement: Supplementary file 10 — Source data Fig. 6 [file 44319_2025_537_MOESM10_ESM.zip › 6G/Klhl8oo--_mRNA.tif]

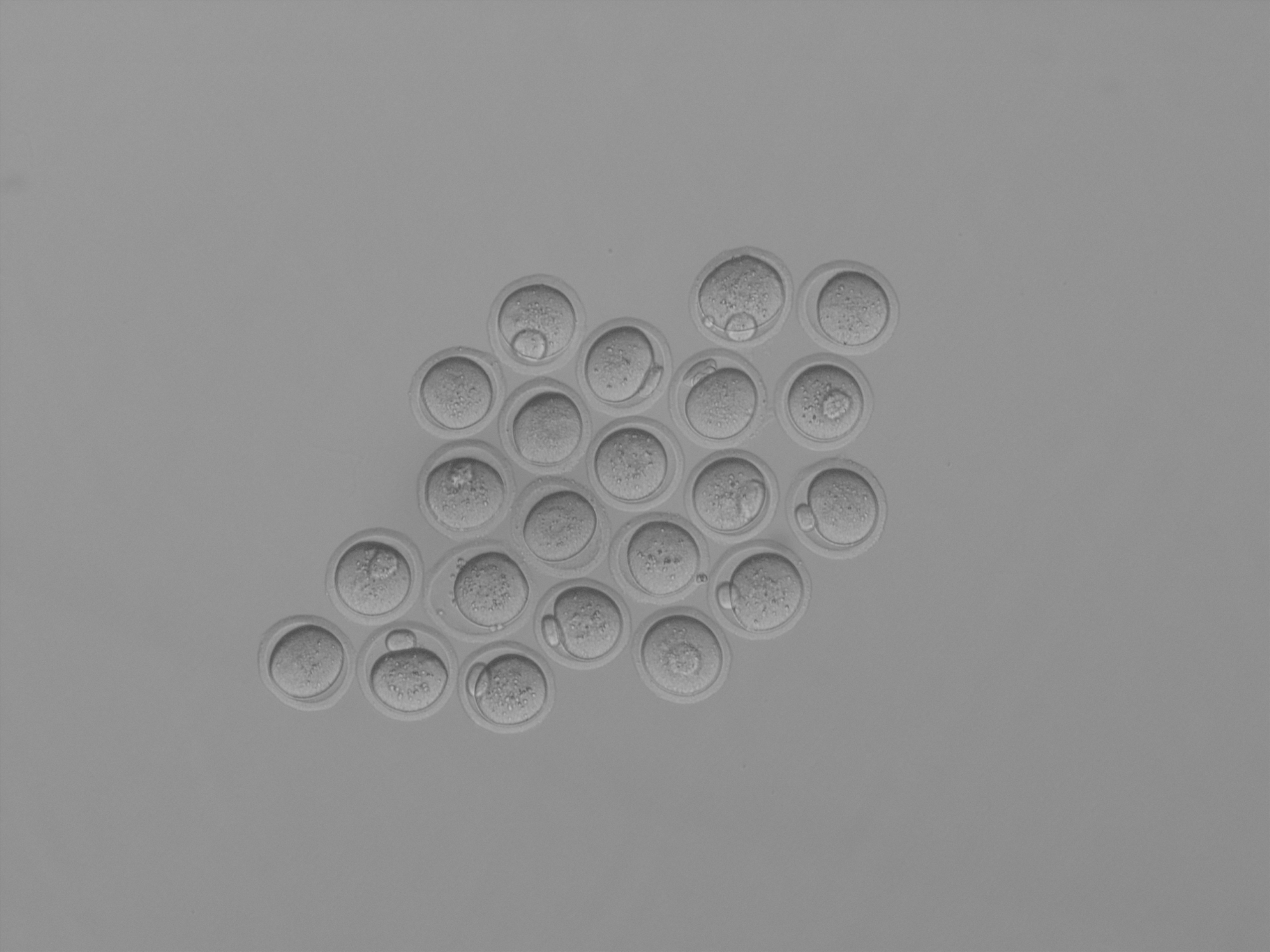

Supplement: Supplementary file 10 — Source data Fig. 6 [file 44319_2025_537_MOESM10_ESM.zip › 6G/WT.tif]

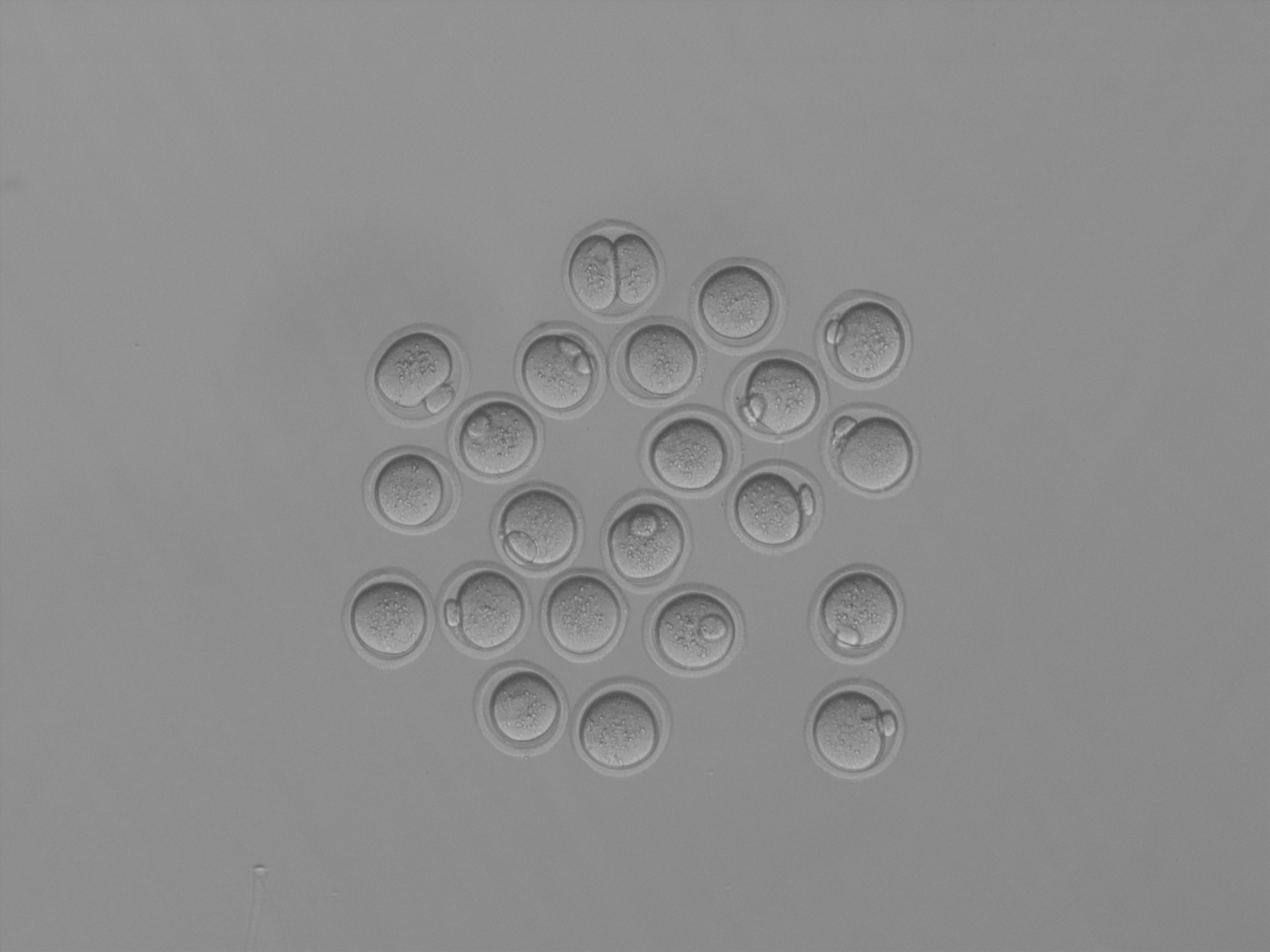

Supplement: Supplementary file 10 — Source data Fig. 6 [file 44319_2025_537_MOESM10_ESM.zip › 6G/WT_mRNA.tif]

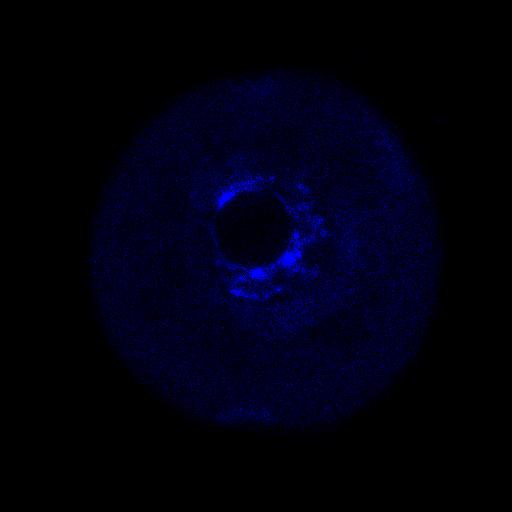

Supplement: Supplementary file 11 — Source data Fig. 7 [file 44319_2025_537_MOESM11_ESM.zip › 7D/Klhl8oo-- GV/Klhl8oo-- GV_DNA.tif]

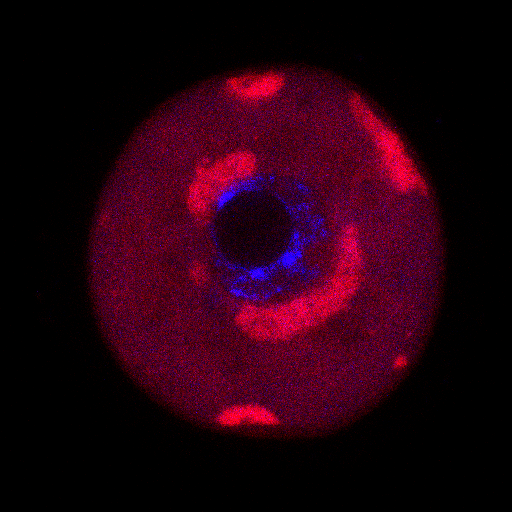

Supplement: Supplementary file 11 — Source data Fig. 7 [file 44319_2025_537_MOESM11_ESM.zip › 7D/Klhl8oo-- GV/Klhl8oo-- GV_merge.tif]

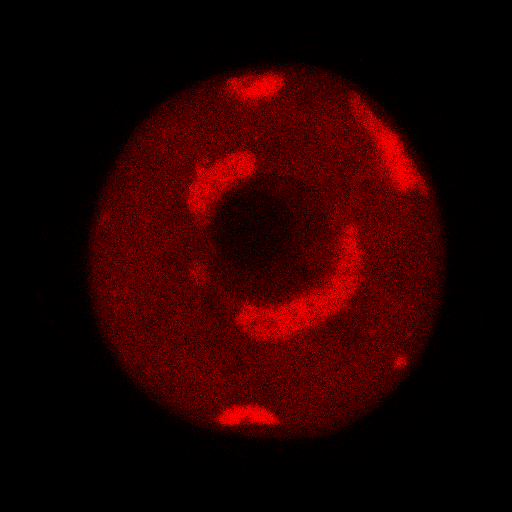

Supplement: Supplementary file 11 — Source data Fig. 7 [file 44319_2025_537_MOESM11_ESM.zip › 7D/Klhl8oo-- GV/Klhl8oo-- GV_oligo dt.tif]

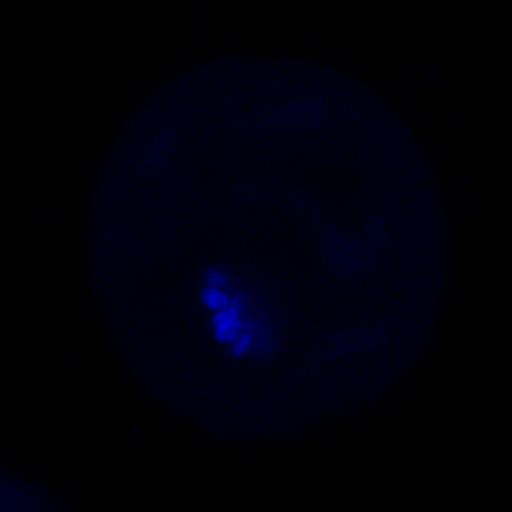

Supplement: Supplementary file 11 — Source data Fig. 7 [file 44319_2025_537_MOESM11_ESM.zip › 7D/Klhl8oo-- MI/Klhl8oo-- MI_DNA.tif]

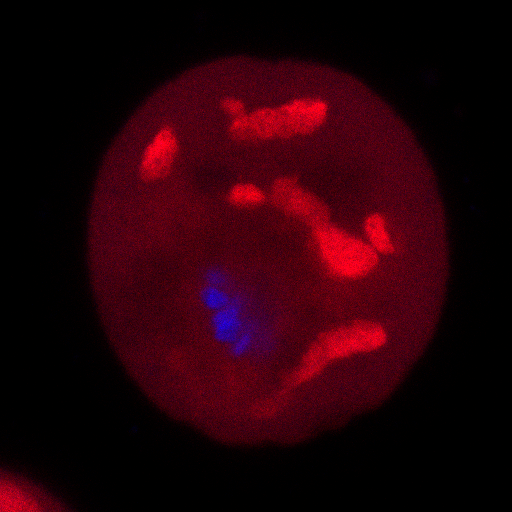

Supplement: Supplementary file 11 — Source data Fig. 7 [file 44319_2025_537_MOESM11_ESM.zip › 7D/Klhl8oo-- MI/Klhl8oo-- MI_merge.tif]

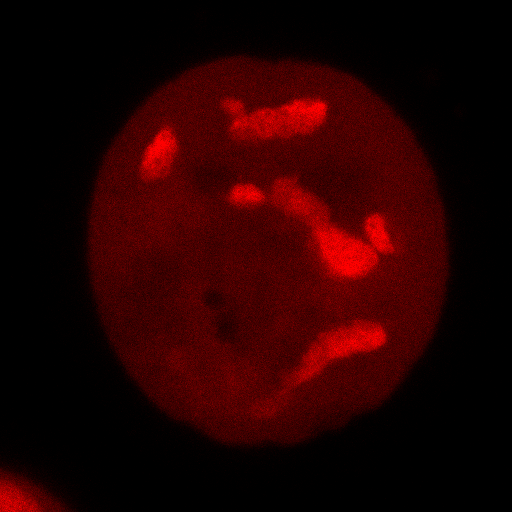

Supplement: Supplementary file 11 — Source data Fig. 7 [file 44319_2025_537_MOESM11_ESM.zip › 7D/Klhl8oo-- MI/Klhl8oo-- MI_oligo dt.tif]

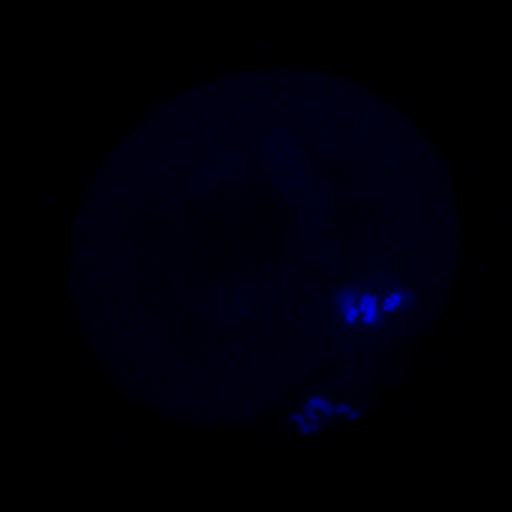

Supplement: Supplementary file 11 — Source data Fig. 7 [file 44319_2025_537_MOESM11_ESM.zip › 7D/Klhl8oo-- MII/Klhl8oo-- MII_DNA.tif]

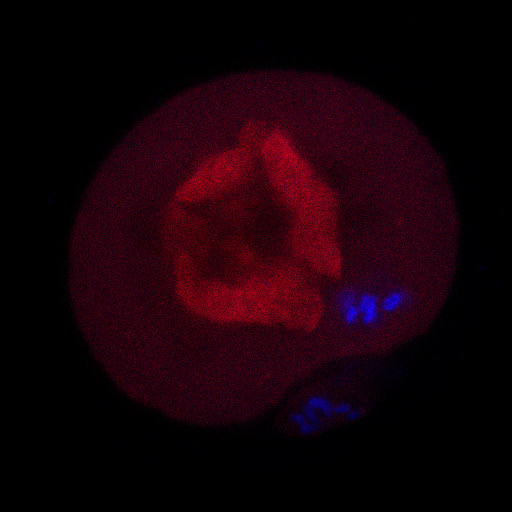

Supplement: Supplementary file 11 — Source data Fig. 7 [file 44319_2025_537_MOESM11_ESM.zip › 7D/Klhl8oo-- MII/Klhl8oo-- MII_merge.tif]

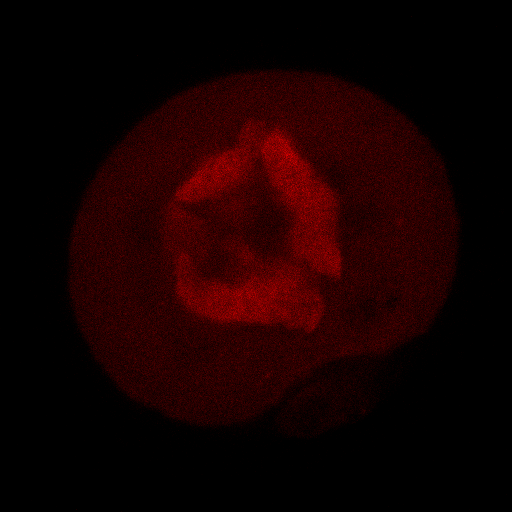

Supplement: Supplementary file 11 — Source data Fig. 7 [file 44319_2025_537_MOESM11_ESM.zip › 7D/Klhl8oo-- MII/Klhl8oo-- MII_oligo dt.tif]

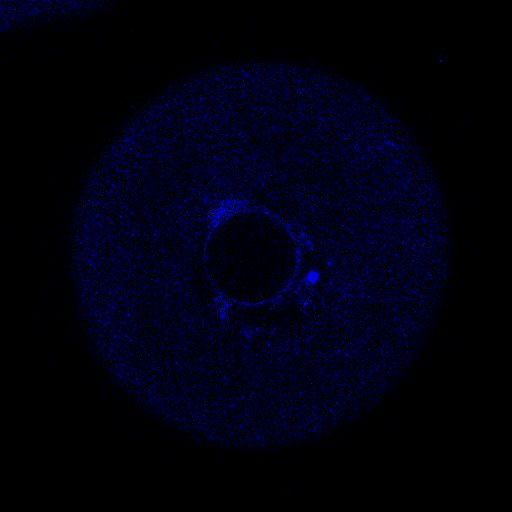

Supplement: Supplementary file 11 — Source data Fig. 7 [file 44319_2025_537_MOESM11_ESM.zip › 7D/WT GV/WT GV_DNA.tif]

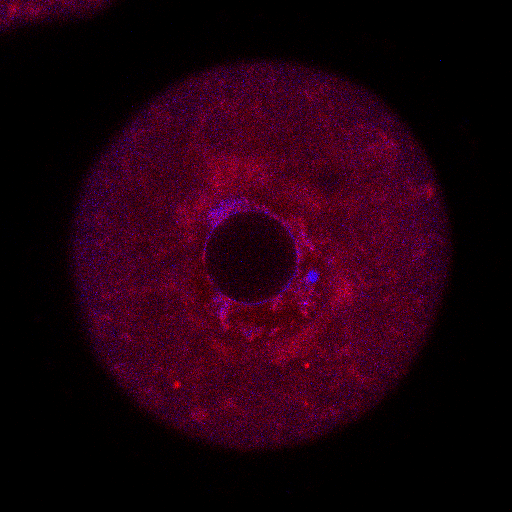

Supplement: Supplementary file 11 — Source data Fig. 7 [file 44319_2025_537_MOESM11_ESM.zip › 7D/WT GV/WT GV_merge.tif]

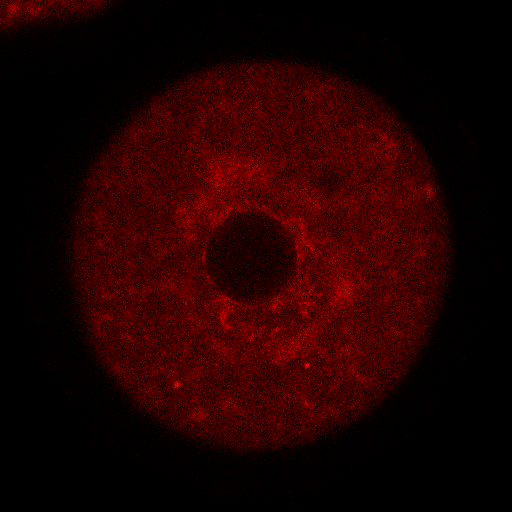

Supplement: Supplementary file 11 — Source data Fig. 7 [file 44319_2025_537_MOESM11_ESM.zip › 7D/WT GV/WT GV_oligo dt.tif]

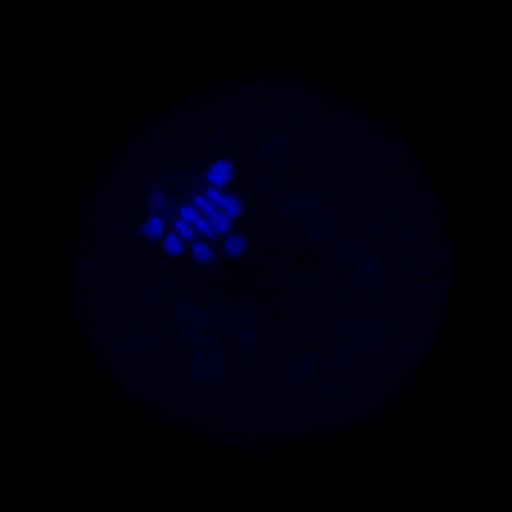

Supplement: Supplementary file 11 — Source data Fig. 7 [file 44319_2025_537_MOESM11_ESM.zip › 7D/WT MI/WT MI_DNA.tif]

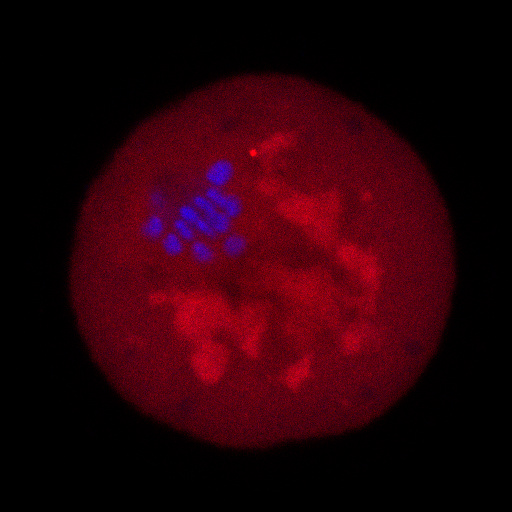

Supplement: Supplementary file 11 — Source data Fig. 7 [file 44319_2025_537_MOESM11_ESM.zip › 7D/WT MI/WT MI_merge.tif]

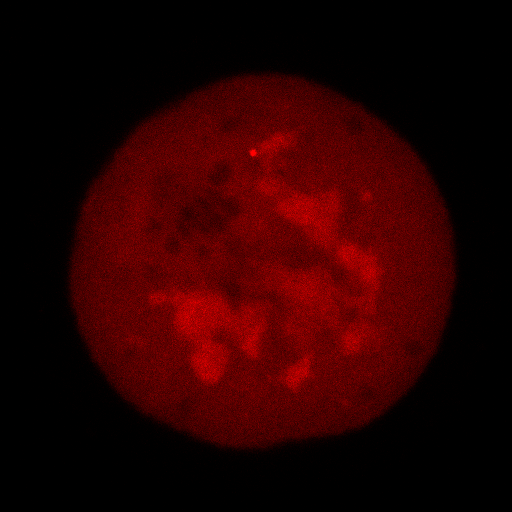

Supplement: Supplementary file 11 — Source data Fig. 7 [file 44319_2025_537_MOESM11_ESM.zip › 7D/WT MI/WT MI_oligo dt.tif]

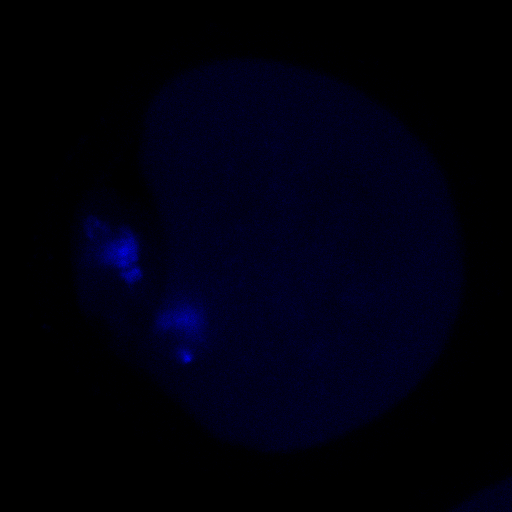

Supplement: Supplementary file 11 — Source data Fig. 7 [file 44319_2025_537_MOESM11_ESM.zip › 7D/WT MII/WT MII_DNA.tif]

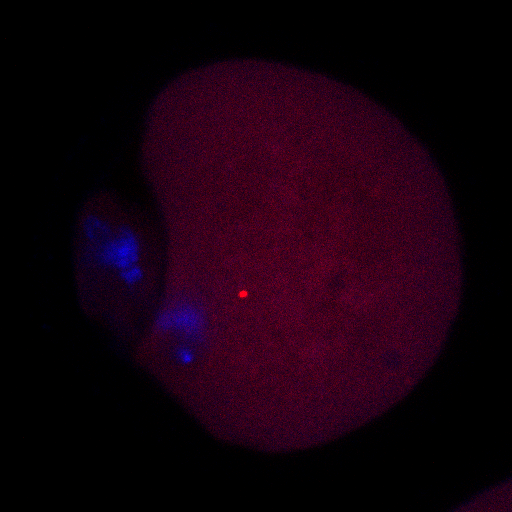

Supplement: Supplementary file 11 — Source data Fig. 7 [file 44319_2025_537_MOESM11_ESM.zip › 7D/WT MII/WT MII_merge.tif]

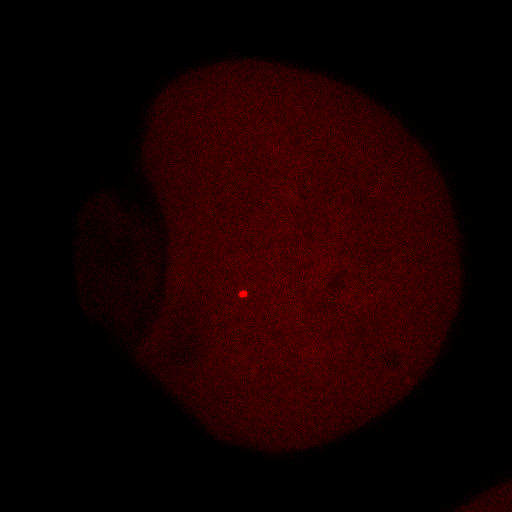

Supplement: Supplementary file 11 — Source data Fig. 7 [file 44319_2025_537_MOESM11_ESM.zip › 7D/WT MII/WT MII_oligo dt.tif]

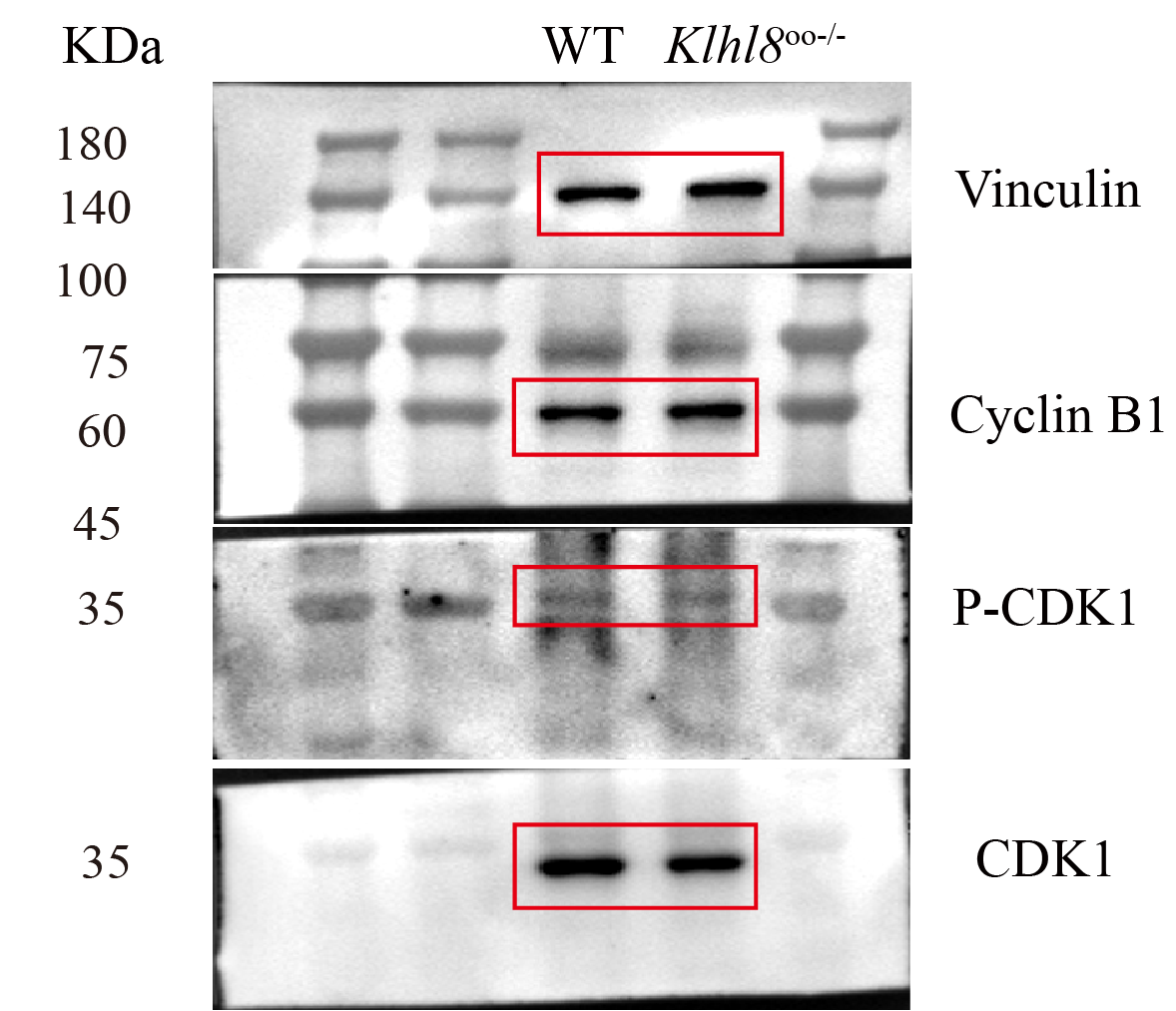

Supplement: Supplementary file 12 — Appendix Figure S3B Source Data [file 44319_2025_537_MOESM12_ESM.zip › S3B.tif]
