# Supplementary material for: The E3 ubiquitin ligase adaptor KLHL8 targets ZAR1 to regulate maternal mRNA degradation in oocytes
Source: EMBO Rep. 2025 Jul 28;26(17):4364–87. doi: 10.1038/s44319-025-00537-y (PMC12420792; doi:10.1038/s44319-025-00537-y)
Supplement: Supplementary file 13 — Expanded View Figures [file 44319_2025_537_MOESM13_ESM.pdf]

Expanded View Figures

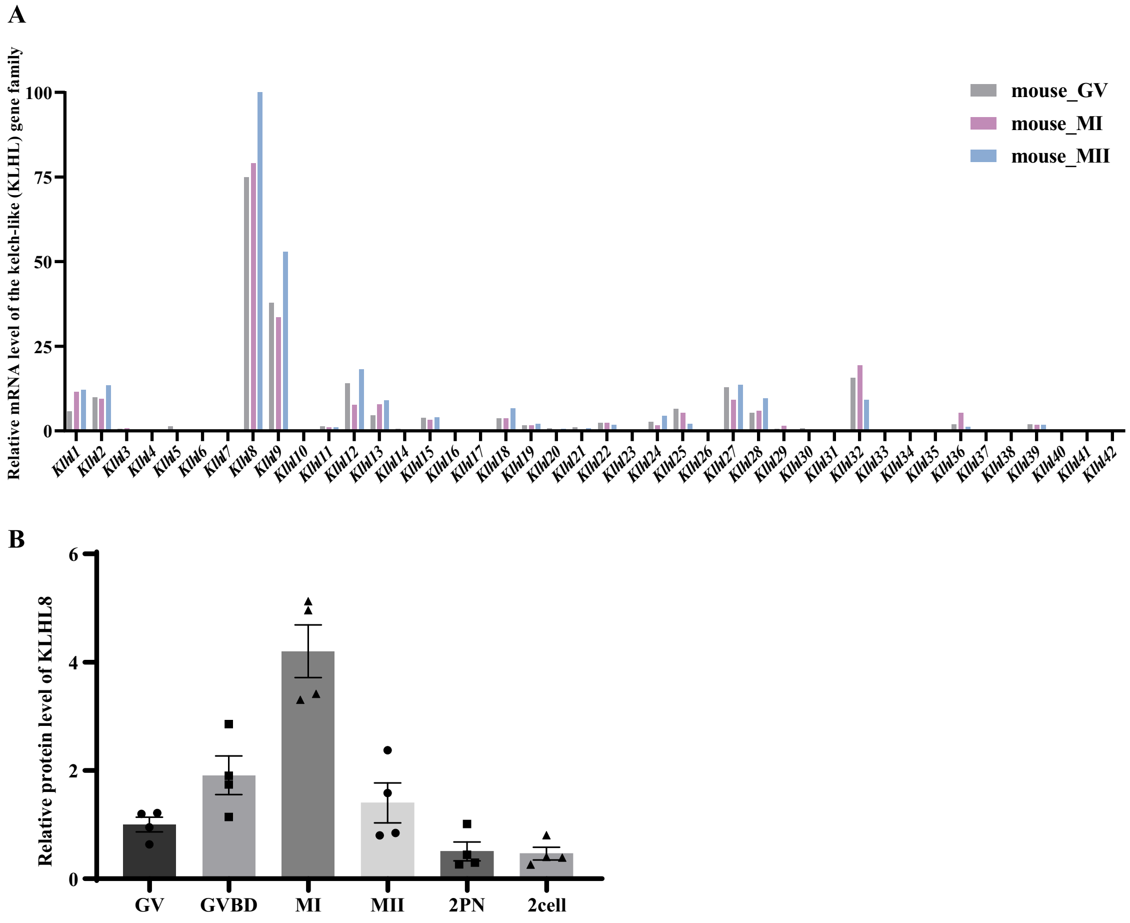

Figure EV1. The transcription level and protein abundance of KLHL8 during oocyte maturation.

(**A**) In-house database qRT-PCR results for KLHL family mRNA expression in GV, MI, and MII oocytes. (**B**) Quantification of KLHL8 protein abundance during oocyte maturation. Data are presented as mean ± SEM ($n = 4$).

© The Author(s)

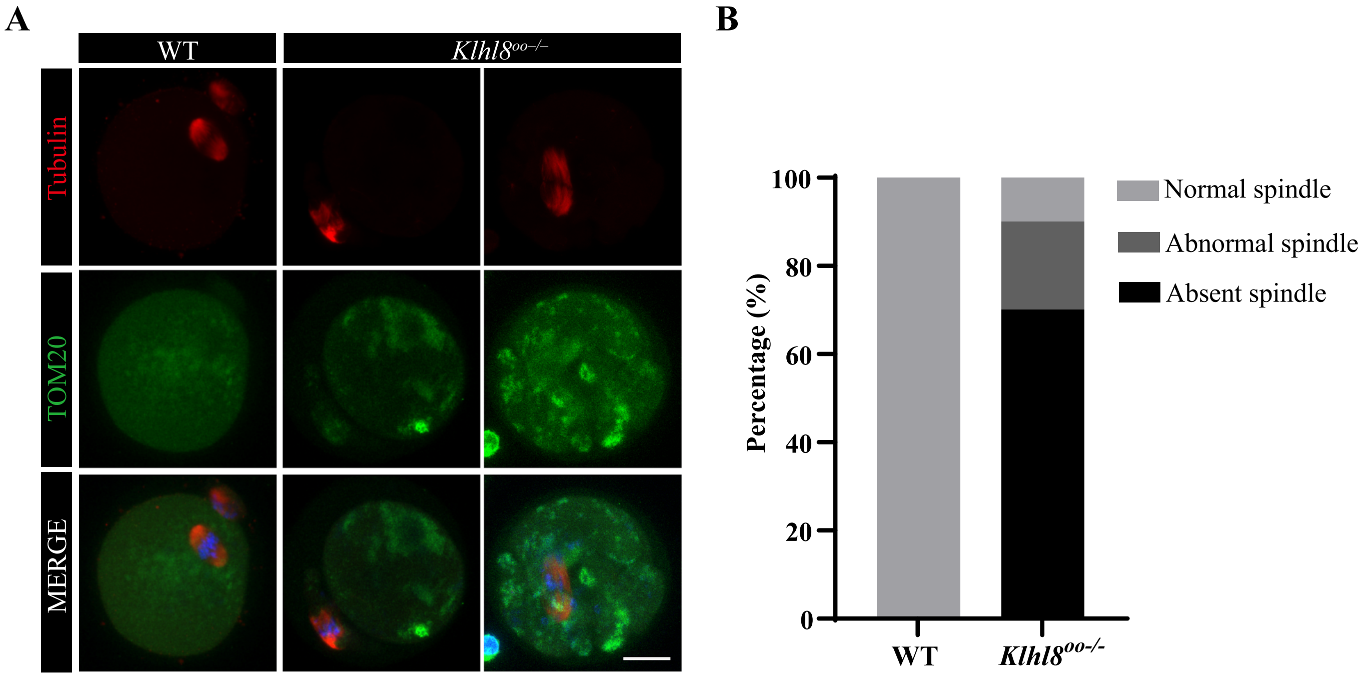

Figure EV2. *Klhl8* deletion causes abnormal spindle in MII oocytes.

(**A**) The spindle morphology in few oocytes that manage to extrude PB1 in vivo. Red, β-Tubulin; Green, mitochondria (TOM20). Scale bar, 20 μm. (**B**) The percentage of oocytes with different spindle morphologies in WT and *Klhl8*$^{oo-/-}$ MII oocytes (WT, $n = 8$; *Klhl8*$^{oo-/-}$, $n = 10$).

 © The Author(s)

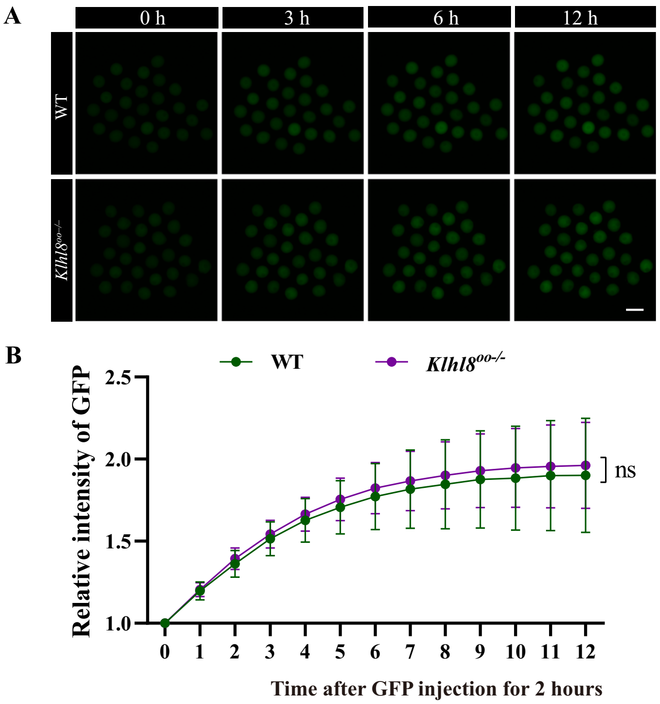

Figure EV3. Translation efficiency in WT and *Klhl8*$^{oo-/-}$ oocytes.

(**A**) GFP expression in WT and *Klhl8*$^{oo-/-}$ GV oocytes after mRNA injection for 2 h. Scale bar, 100 μm. (**B**) The quantification of GFP intensity in WT and *Klhl8*$^{oo-/-}$ GV oocytes (WT, *n* = 28; *Klhl8*$^{oo-/-}$, *n* = 29). Data are presented as mean ± SD. Statistical significance was determined using Multiple unpaired *t* test: ns not significant.

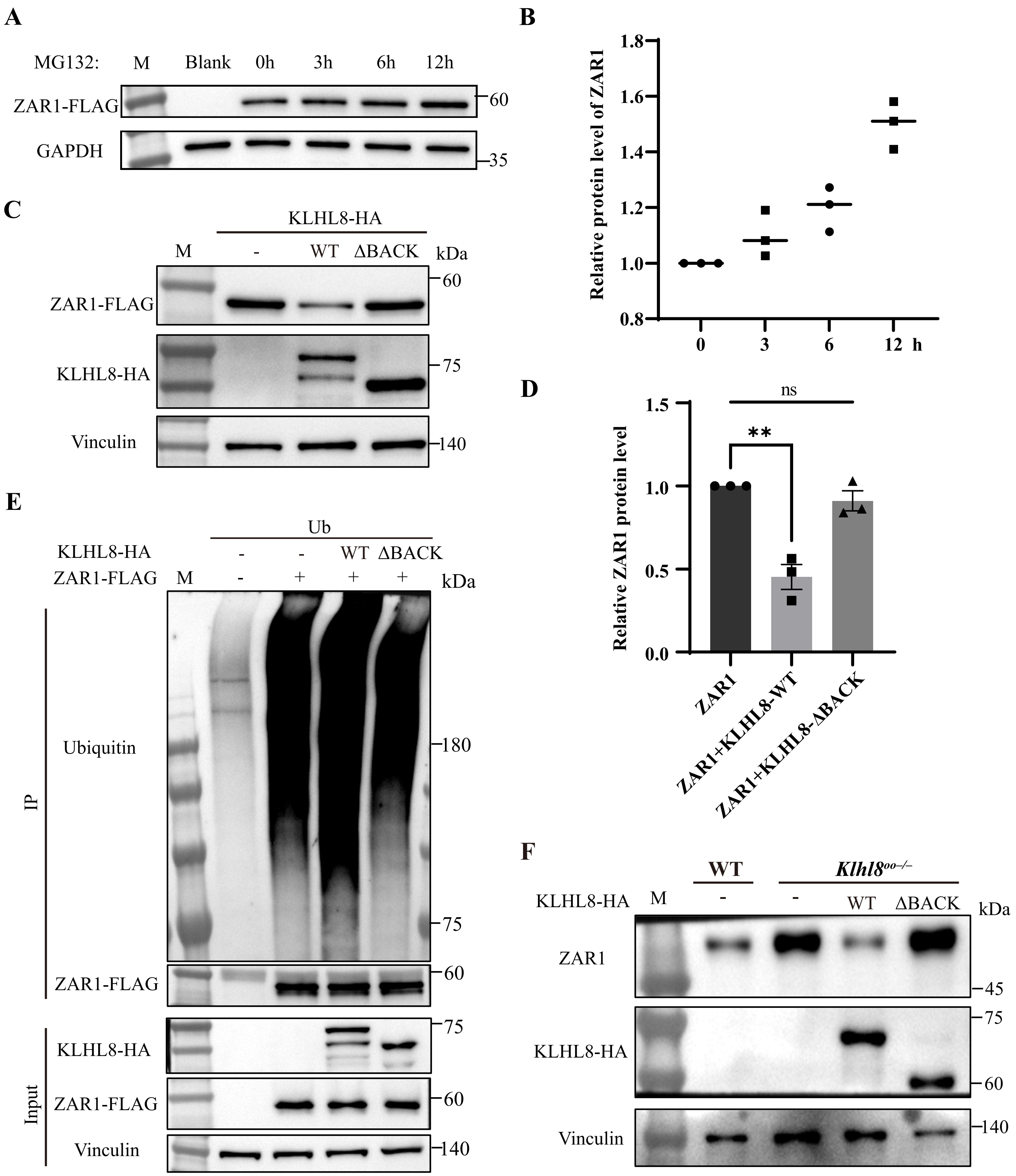
A
MG132: M Blank 0h 3h 6h 12h
ZAR1-FLAG
GAPDH
60
35
B
Relative protein level of ZAR1
1.8
1.6
1.4
1.2
1.0
0.8
0 3 6 12 h
C
KLHL8-HA
M - WT ΔBACK kDa
ZAR1-FLAG
KLHL8-HA
Vinculin
60
75
140
D
Relative ZAR1 protein level
ns
**
1.5
1.0
0.5
0.0
ZAR1
ZAR1+KLHL8-WT
ZAR1+KLHL8-ΔBACK
E
Ub
KLHL8-HA - - WT ΔBACK
ZAR1-FLAG M - + + + kDa
IP
Ubiquitin
ZAR1-FLAG
Input
KLHL8-HA
ZAR1-FLAG
Vinculin
180
75
60
75
60
140
F
WT
Klhl8oo−/−
KLHL8-HA M - - WT ΔBACK kDa
ZAR1
KLHL8-HA
Vinculin
45
75
60
140

 © The Author(s)

Figure EV4. BACK domain of KLHL8 is necessary for ZAR1 ubiquitination and proteasome degradation.

(**A**) Western blot result showing ZAR1 protein level after adding MG132. (**B**) Quantification of the ZAR1 protein level after adding MG132 for different duration. Data are presented as mean ± SEM ($n = 3$). (**C**) Immunoblotting for ZAR1 with *Klhl8*-WT or *Klhl8*-ΔBACK overexpression in cultured HeLa cells. (**D**) Quantitative analysis of ZAR1 protein level with *Klhl8*-WT or *Klhl8*-ΔBACK overexpression in cultured HeLa cells. Data are presented as mean ± SEM ($n = 3$). Statistical significance was determined using an unpaired two-tailed *t* test: ns not significant, $^{**}P = 0.0018$. (**E**) The ubiquitination of ZAR1 with *Klhl8*-WT or *Klhl8*-ΔBACK overexpression in cultured HeLa cells. (**F**) Immunoblotting for ZAR1 in WT and $Klhl8^{oo-/-}$ GV oocytes with *Klhl8*-WT or *Klhl8*-ΔBACK mRNA injection. Vinculin was used as the protein loading control.

© The Author(s)

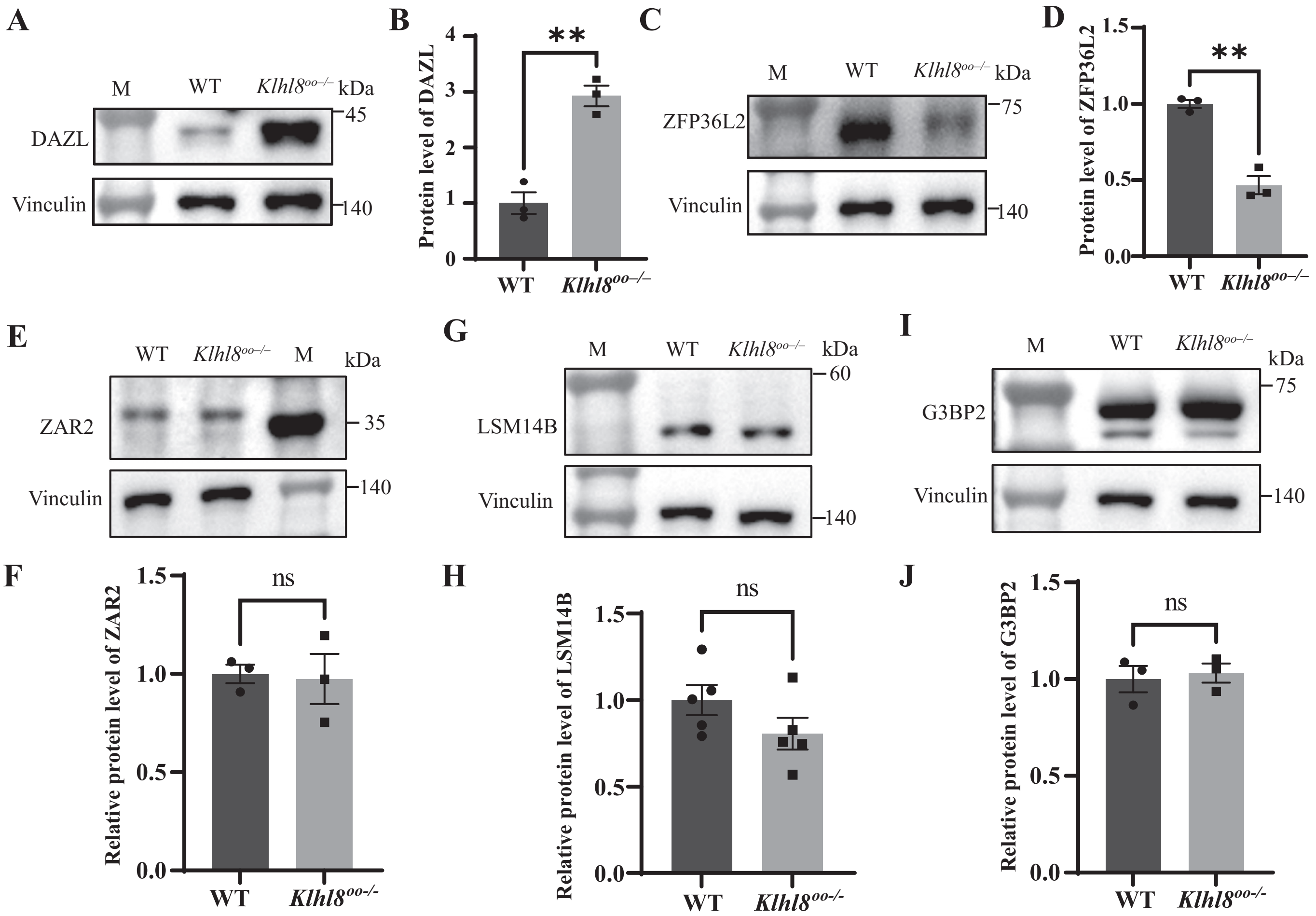

Figure EV5. Effects of *Klhl8* deletion on other RNA binding proteins.

(**A**, **B**) Western blot result and quantitative analysis of DAZL protein level in WT and *Klhl8*$^{oo-/-}$ oocytes. Vinculin was used as the protein loading control. Data are presented as mean ± SEM ($n = 3$). Statistical significance was determined using an unpaired two-tailed *t* test: $^{**}P = 0.002$. (**C**, **D**) Western blot result and quantitative analysis of ZFP36L2 protein level in WT and *Klhl8*$^{oo-/-}$ oocytes. Vinculin was used as the protein loading control. Data are presented as mean ± SEM ($n = 3$). Statistical significance was determined using an unpaired two-tailed *t* test: $^{**}P = 0.0012$. (**E**, **F**) Western blot result and quantitative analysis of ZAR2 protein level in WT and *Klhl8*$^{oo-/-}$ oocytes. Vinculin was used as the protein loading control, $n = 3$. (**G**, **H**) Western blot result and quantitative analysis of LSM14B protein level in WT and *Klhl8*$^{oo-/-}$ oocytes. Vinculin was used as the protein loading control, $n = 5$. (**I**, **J**) Western blot result and quantitative analysis of G3BP2 protein level in WT and *Klhl8*$^{oo-/-}$ oocytes. Vinculin was used as the protein loading control, $n = 3$. In (**F**, **H**, **J**), data are presented as mean ± SEM. Statistical significance was determined using an unpaired two-tailed *t* test: ns not significant.

 © The Author(s)
